# Deep Learning-Based Automatic Duckweed Counting Using StarDist and Its Application on Measuring Growth Inhibition Potential of Rare Earth Elements as Contaminants of Emerging Concerns

**DOI:** 10.3390/toxics11080680

**Published:** 2023-08-08

**Authors:** Kevin Adi Kurnia, Ying-Ting Lin, Ali Farhan, Nemi Malhotra, Cao Thang Luong, Chih-Hsin Hung, Marri Jmelou M. Roldan, Che-Chia Tsao, Tai-Sheng Cheng, Chung-Der Hsiao

**Affiliations:** 1 Department of Chemistry, Chung Yuan Christian University, Chung-Li 32023, Taiwan; kevinadik-adi@hotmail.com (K.A.K.); smalifarhan@gmail.com (A.F.); 2 Department of Bioscience Technology, Chung Yuan Christian University, Chung-Li 32023, Taiwan; nemi.malhotra@gmail.com; 3 Department of Biotechnology, College of Life Science, Kaohsiung Medical University, Kaohsiung City 80708, Taiwan; ytlin@kmu.edu.tw; 4 Drug Development & Value Creation Research Center, Kaohsiung Medical University, Kaohsiung City 80708, Taiwan; 5 Department of Chemical Engineering & Institute of Biotechnology and Chemical Engineering, I-Shou University, Da-Shu, Kaohsiung City 84001, Taiwan; thang.luongcao@gmail.com (C.T.L.); chhung@isu.edu.tw (C.-H.H.); 6 Faculty of Pharmacy, The Graduate School, University of Santo Tomas, Manila 1008, Philippines; mmroldan@ust.edu.ph; 7 Department of Biological Sciences and Technology, National University of Tainan, Tainan 70005, Taiwan; ctsao@mail.nutn.edu.tw; 8 Center for Nanotechnology, Chung Yuan Christian University, Chung-Li 32023, Taiwan; 9 Research Center for Aquatic Toxicology and Pharmacology, Chung Yuan Christian University, Chung-Li 32023, Taiwan

**Keywords:** duckweed, StarDist, deep learning, cell counting, image segmentation, rare earth element

## Abstract

In recent years, there have been efforts to utilize surface water as a power source, material, and food. However, these efforts are impeded due to the vast amounts of contaminants and emerging contaminants introduced by anthropogenic activities. Herbicides such as Glyphosate and Glufosinate are commonly known to contaminate surface water through agricultural industries. In contrast, some emerging contaminants, such as rare earth elements, have started to enter the surface water from the production and waste of electronic products. Duckweeds are angiosperms from the *Lemnaceae* family and have been used for toxicity tests in aquatic environments, mainly those from the genus *Lemna*, and have been approved by OECD. In this study, we used duckweed from the genus *Wolffia*, which is smaller and considered a good indicator of metal pollutants in the aquatic environment. The growth rate of duckweed is the most common endpoint in observing pollutant toxicity. In order to observe and mark the fronds automatically, we used StarDist, a machine learning-based tool. StarDist is available as a plugin in ImageJ, simplifying and assisting the counting process. Python also helps arrange, manage, and calculate the inhibition percentage after duckweeds are exposed to contaminants. The toxicity test results showed Dysprosium to be the most toxic, with an IC_50_ value of 14.6 ppm, and Samarium as the least toxic, with an IC_50_ value of 279.4 ppm. In summary, we can provide a workflow for automatic frond counting using StarDist integrated with ImageJ and Python to simplify the detection, counting, data management, and calculation process.

## 1. Introduction

In recent years there have been efforts to use oceans and seas as a source of energy, raw materials, and food. However, these areas are prone to contaminants exposure which attracted public attention. The most common source of these contaminants comes from anthropogenic activities. It can be introduced through rivers, direct discharges, or atmospheric deposition [1]. One of the most common pollutants is herbicides. Herbicides target duckweed species that have detrimental effects on crops specifically. In contrast, some of them are even used illegally to control algae in aquatic environments [2]. The reliance on herbicides induces the emergence of herbicide-resistant biotypes while polluting the environment and threatening the health of aquatic organisms [3]. The result of previous studies showed that exposure to both herbicides to zebrafish showed an increase in ROS levels, morphological alteration, and behavioral changes in both larvae and adult stages of zebrafish [4,5]. Recent studies classified rare earth elements (REE) as emerging contaminants (EC). They are metals found in group IIIB in the periodic table. It has similar physical and chemical properties; therefore, it is widely used in industrial products, mainly electricals (such as television, phone, and laptop). They have been introduced to marine systems but are often overlooked due to a poor understanding of their toxic effects and low detection limit. While the toxicity of REEs has been previously shown on zebrafish larvae, a limited understanding of each element was still present [6].

*Lemnaceae* comprises the world’s smallest angiosperms, duckweeds [7], and monocotyledonous water plants [8,9]. *Lemnaceae* includes five aquatic genera, *Spirodela*, *Wolffia*, *Wolffiela*, *Lemna*, and *Landottia*, primarily used for toxicity testing in aquatic waterbodies. The scientific literature reveals that most of all, i.e., 95% of toxicity tests are performed on species from the genus *Lemna* [10]. Duckweeds are the earth’s tiniest angiosperms [11].

The small size, rapid reproduction, fast growth [12], and high metal and nutrient accumulation have triggered metal tolerance for duckweed species. Furthermore, it has also been reported that metal tolerance varies with water environmental conditions (i.e., temperature, pH, metal concentration, and electrical conductivity) [7,13]. Duckweeds are regarded as ideal organisms for experimentation in many different disciplines of plant sciences, i.e., toxicology, physiology, and ecology [14]. Almost all genera have been stated to have phytoremediation potential [15]. The properties of small size, fast growth rate, rapid reproduction, easy maintenance in the lab environment, and visual observation make duckweed potential source for research in several different disciplines such as wastewater treatment, ecotoxicity analysis, generating clean, renewable, ecofriendly energy [13,16]. Duckweeds are used as test organisms in ecotoxicological testing for detecting metals in wastewater/surface waters because of their hyperaccumulation potential, fast vegetative propagation, and short lifespan [17]. In aquatic environments such as rivers, lakes, and groundwater, duckweed toxicity tests are highly versatile as they help detect complex effluents and single chemical compounds from industrial or community waste in the form of inorganic or organic compounds [18,19,20,21]. In addition, *Lemna* species are also used in large-scale experiments worldwide due to their relevance as primary producers and major food sources for water/aquatic species such as small invertebrates and fish and a swarm for other small organisms [10].

The *Wolffia* species has been used as a food source in Asian countries. *Wolffia globosa* is rootless duckweed and a good indicator of metal pollution in water bodies [22]. It has been recorded as the world’s fastest-growing plant, capable of doubling its number as early as 1–2 days in optimal conditions. These tiny plants can grow optimally at 20–30 °C while also growing in cold conditions at lower temperatures of 1–3 °C; however, serious implications have been observed at high temperature ranges from 35–40 °C. Moreover, duckweeds have been shown to withstand a varied range of pH 3.0–10.0 with optimum growth in medium with pH 5.0–7.0.

The current method for duckweed frond quantification mainly revolves around manual counting [23,24], the implementation of ImageJ [25], and paid software [26,27]. ImageJ helps with the quantification process as it is possible to measure the area covered by duckweed and the number of duckweeds. However, processing many images might be challenging without the use of macro or automation, and the result might differ from time to time, depending on person to person (Table 1). Additionally, the detection using ImageJ by itself might also be limited due to overlapping and tightly packed objects [28], which will happen in the case of duckweed as the fronds are close along with overlapping. Currently, there is also an available commercial platform called LemnaTec. This platform uses preprogrammed image segmentation methods to detect and measure duckweed sizes [26]. Haffner et al. proposed a visual system that includes a camera holder, a light holder, a camera, a light, a voltage source, and a computer for image acquisition. They developed an algorithm implemented in NI Vision Assistant software to count and measure the area of each duckweed [27]. However, as mentioned earlier, these programs are commercial, as there is a need to purchase/subscribe to their respective company; therefore, researchers who just started doing duckweed study might lean toward using ImageJ as free software.

There have been studies that used artificial intelligence/machine learning methods to overcome contaminated water through machine learning applications [33] and quantify cell growth for toxicity studies in other species/cell lines [34,35]. The use of a machine learning-based method might be a solution to the limitation of ImageJ limitation as free software. Therefore, we employed StarDist as a plugin in ImageJ automatically detect each duckweed frond and count those fronds to simplify the counting process. StarDist uses a neural network that predicts objects based on predetermined shapes, which is a star-convex polygon [36,37]. StarDist was initially developed for cell nuclei. However, applying this network to detect and measure duckweed might be possible due to having a similar circular shape to cell nuclei in 2D images. In this study, we applied the automated deep learning system StarDist to provide a new automatic counting workflow to observe duckweed growth.

Additionally, the duckweed was exposed to herbicide and REE as emerging contaminants to prove the compatibility of the toxicity test by measuring duckweed growth in response to the toxic effect of organic/inorganic chemical effluent present in the provided media. Currently, there are minimal studies on the effect of REE on duckweed, and most of them only observe the effect of one element. Therefore, we will test the toxicity effect of 14 different REEs on duckweed to provide a more comprehensive report on REE toxicity. In this study, we expect LREE to be more toxic than HREE, as reported by the previous study performed in a zebrafish model system [38].

## 2. Material and Methods

### 2.1. Duckweed Culture

The common watermeal (*Wolffia globosa*) stock was a gift from Lemnaceae Fermentation, Inc. (Taoyuan City, Taiwan). The stock was kept at the laboratory and cultured in Hyponex No.4 fertilizer (Hyponex Japan Co., Ltd., Osaka, Japan) with 1:10,000 dilution in water. The light illumination was kept at 400 lux. Common watermeal was transferred to 6-well microplates using plastic straw before toxic compounds exposure. The initial density of common watermeal was maintained at low coverage. Duckweeds were acclimated to laboratory conditions for at least four days before the toxicity test.

### 2.2. Preparation of Stock Solution

Two kinds of Glyphosate and Glufosinate herbicides were used as known plant growth inhibitors. These herbicides were diluted to a stock concentration of 10,000 ppm. Additionally, duckweeds were also exposed to several different rare earth elements (REE), including Lanthanum (La), Cerium (Ce), Praseodymium (Pr), Neodymium (Nd), Samarium (Sm), Europium (Eu), Gadolinium (Gd), Terbium (Tb), Dysprosium (Dy), Holmium (Ho), Erbium (Er), Thulium (Tm), Ytterbium (Yb), and Lutetium (Lu). REEs are purchased in chloride salt form from Aladdin (Shanghai, China). The stock solutions of REE were prepared by diluting REE solids in ddH_2_O to 10,000 ppm. These stock solutions are diluted to several different concentrations for exposure.

### 2.3. Toxicity Test and Image Acquisition

Ten (10) mL of herbicides at 100 ppb, one ppm, ten ppm, 25 ppm, 50 ppm, and 100 ppm, 200 ppm were added to an empty well of a 6-well microplate. These concentrations were based on previous studies in other duckweed species (*Lemna* sp.) [39,40]. Meanwhile, preliminary IC_50_ tests were performed on REE groups at 100 ppb, one ppm, ten ppm, 100 ppm, and 1000 ppm. Afterward, several concentrations were added to supplement the data at three different concentrations depending on the preliminary test result. A total of 30–40 duckweed fronds were added to each well for a toxicity test. The number of duckweed was kept low to avoid overcrowding the wells and limiting duckweed growth. Duckweed images were taken using Zebrabox (ViewPoint 3.22.3.85, Viewpoint Life Sciences, Inc., Civrieux, France) at HD quality (1024 × 768 pixels), starting 0 h after exposure and seven days after exposure. IC_50_ value was calculated using GraphPad Prism 8 version 8.0.2 (GraphPad Software, Inc., San Diego, CA, USA).

### 2.4. Image Training by StarDist

Five different images of duckweed cultured on a 6-well microplate were taken in addition to daily recording. A small part of these recordings was cropped with a minimum of 256 × 256 pixels for StarDist neural network training using FIJI build of ImageJ (Available for download at: https://imagej.net/software/fiji/, accessed on 18 February 2022). Afterward, each duckweed frond was manually annotated using QuPath v0.3.0 (Available for download at: https://qupath.github.io/, accessed on 18 February 2022). Annotated images were exported to a new folder, and the StarDist neural network was trained in Anaconda (Available for download at: https://www.anaconda.com/products/individual, accessed on 18 February 2022) based on the guidelines provided in StarDist’s GitHub page (https://github.com/stardist/stardist, accessed on 18 February 2022). The trained neural network will be exported into a zip file for image prediction in ImageJ (Supplementary Materials).

### 2.5. Duckweed Detection and Counting

Firstly, we convert the video recording we have using VirtualDub2 software (build 44282) to .avi format due to ImageJ video format limitation (Available for download at: http://virtualdub2.com/, accessed on 18 February 2023). Afterward, we can open the videos in ImageJ and take five images from each video for counting. Then, we can apply a trained neural network using the StarDist plugin, which can be installed according to the StarDist page on the ImageJ website (https://imagej.net/plugins/stardist, accessed on 18 February 2022). Based on the training result, we set the Probability Threshold at 0.82. StarDist will detect and mark all objects recognized as duckweed and add them to ImageJ ROI Manager. Finally, we can run the measure command in the ROI Manager to obtain each image’s total duckweed count according to StarDist selection, then export the result to individual Microsoft Excel files depending on their treatments. We packaged the workflow for duckweed detection and counting in an ImageJ macro script (Supplementary Materials).

### 2.6. Mass Data Processing Using Python-Based Scripts

In order to calculate the *W. globosa* growth rate, we compared the number of counted fronds of each time points to the first recording (day 0), which will show how much the fronds multiply by using this equation:

$$DXfm=DayX\;fronds\;multiplication=\frac{DayX\;fronds\;count}{Day0\;fronds\;count}$$

Then the result of each treatment group was compared to the control group to obtain the inhibition percentage of each treatment by using the equation:

$$\%inhibition=\frac{Control\;DXfm-Treatment\;DXfm\;}{Control\;DXfm}\times 100\%$$

We provided a more extensive and detailed procedure of StarDist operation in Supplementary Materials (also see on-line tutorial at https://www.youtube.com/watch?v=l5ed5yfAmlU&list=PLRmQXf_nHOvP1YUV1xBtQHkDEcLt16rDP, accessed on 2 August 2023).

### 2.7. Statistical Tests

Statistical tests were performed using Graphpad Prism 8 (Graphpad Holdings, LCC, San Diego, CA, USA). The data were transformed to log^10^ values (10 to 1, 100 to 2, 1000 to 3, etc.) in order to stabilize the variance between concentration values. The data were then subjected to nonlinear curve fitting for log(inhibition) vs. normalized response—variable slope to calculate and present IC_50_ concentrations at different time points.

## 3. Results

### 3.1. Overview of Experimental Workflow

In this study, we proposed a deep-learning-based method to develop a faster and more precise tool to assist researchers in counting duckweed fronds, mainly for toxicity tests. The deep learning method we used in this study is StarDist, a neural network initially made to detect star-convex polygons which should be suitable to duckweed fronds shape as they are mostly circular or oval shaped. The Duckweed species we used to test this method is *Wolffia globosa*, commonly known as common watermeal. This species was used as a model organism due to its exceptional growth rate and ease of maintenance.

**Figure 1:**
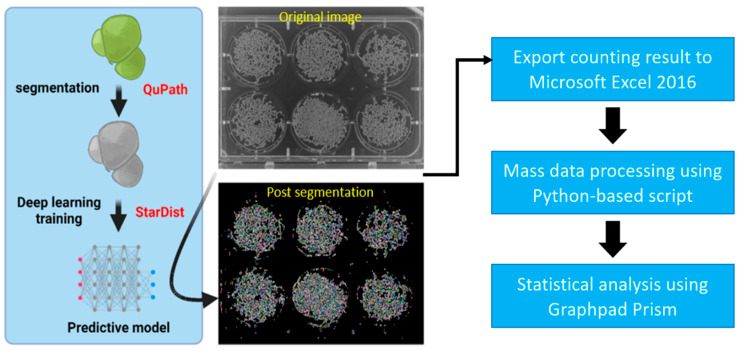
Experimental workflow of duckweed quantification by using the StarDist method. *Wolffia globosa* StarDist model was trained using pre-annotated *Wolffia* images in QuPath. After the model was trained, it can be applied to similar *Wolffia* images using StarDist plugin in ImageJ, to be used for counting duckweed fronds. The counting result was then exported to Microsoft Excel 2016 and processed using a Python-based script to reduce the overall workload. Finally, the data were statistically analyzed using GraphPad Prism.

First, we prepared a training dataset for the predictive model. This process started by manually segmenting prerecorded common watermeal images in QuPath. The manual segmentation result was exported as masked images through a macro provided by the StarDist developer team. Then, the images were used to train the neural network using the StarDist framework in Anaconda. The result of the training was exported in a compressed file for future prediction. The StarDist plugin in ImageJ applies the predictive model to one 6-well plate of *W. globosa* to count their fronds and export the result into an Excel file. The process was repeated until all the videos had their fronds counted. Afterward, we applied a Python-based script to merge and manage all the Excel files, classifying the data based on their treatment and time points. Then, the inhibition percentage of each treatment group was calculated by comparing the treatment group’s fronds growth to the control. Finally, we used a nonlinear regression tool in GraphPad Prism to observe the inhibition effects of compounds of interest on common watermeal (analysis pipeline summarized in Figure 1).

### 3.2. Segmentation Performance for Common Watermeal (Wolffia globosa)

**Figure 2:**
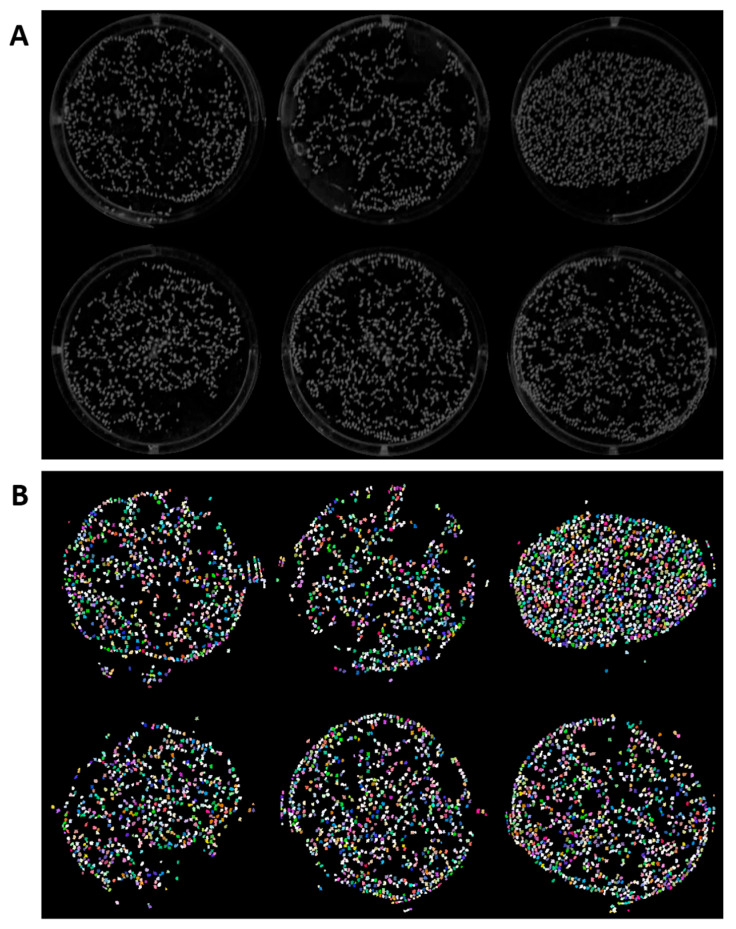
Original (**A**) and segmentation result (**B**) of common watermeal using ImageJ build of StarDist. StarDist plugin in ImageJ is able to detect the *Wolffia globosa* fronds in 6-well plates. However, there are several misdetections caused by fronds reflection on the sides of the wells. The different colors in the image marks individual *Wolffia globosa* fronds.

The segmentation result of duckweed fronds using a trained predictive model and StarDist recommended threshold setting (overlap/NMS threshold = 0.4) is presented in Figure 2. The result showed proper segmentation of duckweed fronds. However, there are several parts showing misdetections. The light reflection on plastic microplates mostly causes these misdetections. In order to reduce this misdetection, wells were individually cropped, reducing misdetection incidence compared to using full microplate images.

### 3.3. Determining the Optimal Overlap/Non-Maximum Suppression (NMS) Threshold for Wolffia globosa Detection Using StarDist 2D

In order to improve StarDist’s performance for detecting *W. globosa*, we experimented with the variables available in StarDist. StarDist used two different kinds of threshold variables to segment objects, probability threshold and overlap/NMS threshold. Probability thresholds are used to classify the objects by scoring them based on their pixel value, while overlap/NMS thresholds are used to prune redundant objects by considering the object intersection area, ensuring the accuracy of the detected object to be ideally the same as the true object. Overlap/NMS threshold is important for *W. globosa* detection. The fronds are located close to each other, which means there is a high chance of overlapping, and new budding fronds might be detected as overlapping with their mother fronds because they are connected. Therefore, we tested the overlap value ranging from 0.1–0.9 to find the most suitable overlap/NMS threshold for duckweed detection.

**Figure 3:**
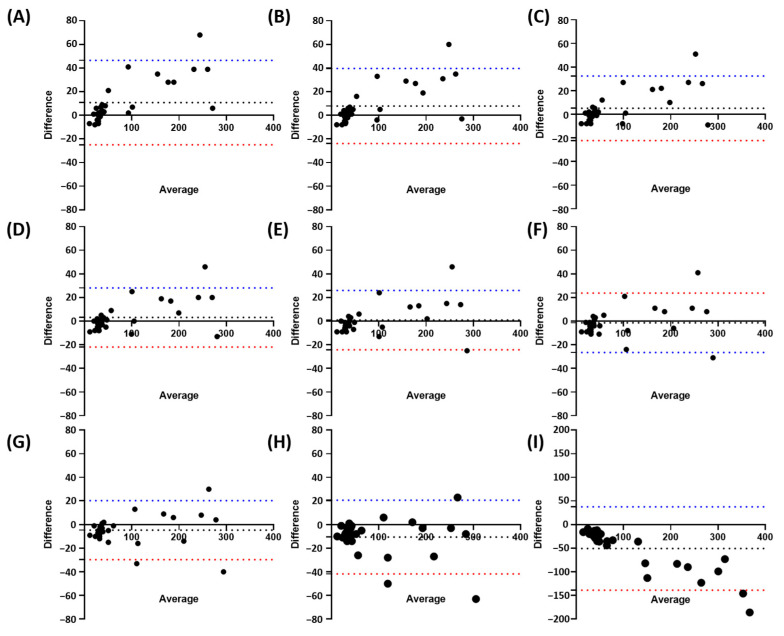
Bland–Altman plots of manual counting compared to different overlap/NMS threshold values; 0.1 (**A**), 0.2 (**B**), 0.3 (**C**), 0.4 (**D**), 0.5 (**E**), 0.6 (**F**), 0.7 (**G**), 0.8 (**H**), and 0.9 (**I**). The black dotted line represents Mean Difference between manual counting and StarDist at different thresholds, while the blue/red dotted line represents the upper/lower limit of (*n* = 30).

In order to test it, we randomly selected 30 *W. globosa* images from different time points. We manually counted those images while applying the StarDist model we previously trained with different overlap/NMS threshold values. From the Bland–Altman plots of the nine different overlap/NMS threshold values, the mean difference was 10.83 for 0.1, 7.90 for 0.2, 5.07 for 0.3, 3.13 for 0.4, 0.90 for 0.5, −1.37 for 0.6, −4.67 for 0.7, −10.57 for 0.8, and −50.57 for 0.9 (Figure 3). These values showed the mean counting difference between the manual counting method used as a standard and the StarDist counting method with different overlap/NMS threshold values. Therefore, the result suggests 0.5 as the most suitable threshold value due to having the lowest mean differences, thus showing the closest counting performance to the manual counting method. However, the Bland–Altman plots result of Figure 3 suggests that higher fronds count leads to lower accuracy, as most of the dots are located further from the mean difference line on a higher frond count.

### 3.4. Optimized StarDist Model for Duckweed Growth Inhibition Assay

The performance of StarDist was tested on duckweed growth inhibition assay using two different herbicides, namely Glyphosate and Glufosinate, along with 14 kinds of REE. We used overlap threshold = 0.5 as it showed the most similar result to manual counting. The result of StarDist was exported into Excel (.xlsx) files. Each Excel file contains the result of one plate and a one-time point. Then, we employed a Python-based script to merge the data from the control and different treatments group into one Excel file to calculate the growth as described above. The script will produce a final Excel file showing each treatment’s growth inhibition rate for each time point. Finally, the data were inputted into GraphPad Prism to generate IC_50_ graphs and calculate IC_50_ values.

**Figure 4:**
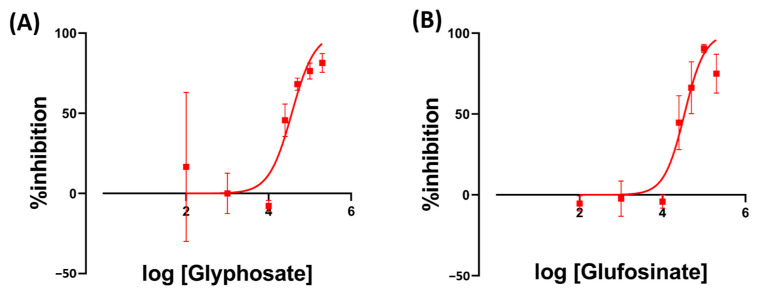
The inhibition rate of *W. globosa* at day seven after exposure to different kinds of herbicides; Glyphosate (**A**) and Glufosinate (**B**) to determine the median inhibitory concentration (IC_50_), data are presented as Mean ± 95% CI (*n* = 3).

**Figure 5:**
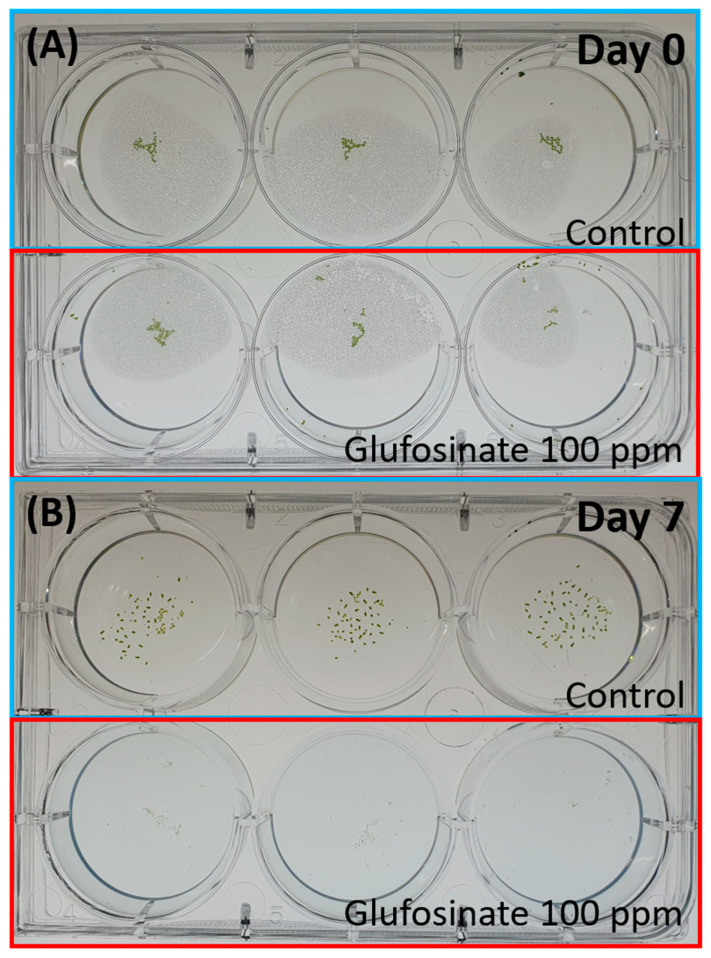
*Wolffia globosa* growth at Day 0 (**A**) and Day 7 (**B**), Top rows are the control group, and the bottom rows are exposed to Herbicides (Glufosinate) at 100 ppm. Bleaching of Wolffia fronds was observed after exposure to Herbicides (Glufosinate).

The result of Glyphosate exposure had IC_50_ values of 36.4 ppm on Day 7. Meanwhile, Glufosinate had an IC_50_ of 33.9 ppm on Day 7 (Figure 4). We also observed the bleaching of *Wolffia* fronds after exposure to Glyphosate and Glufosinate (Figure 5). The bleaching happened as soon as the first day after exposure to the high herbicide concentration.

### 3.5. Exposure to Rare Earth Elements Alters Wolffia globosa Growth Rate

**Figure 6:**
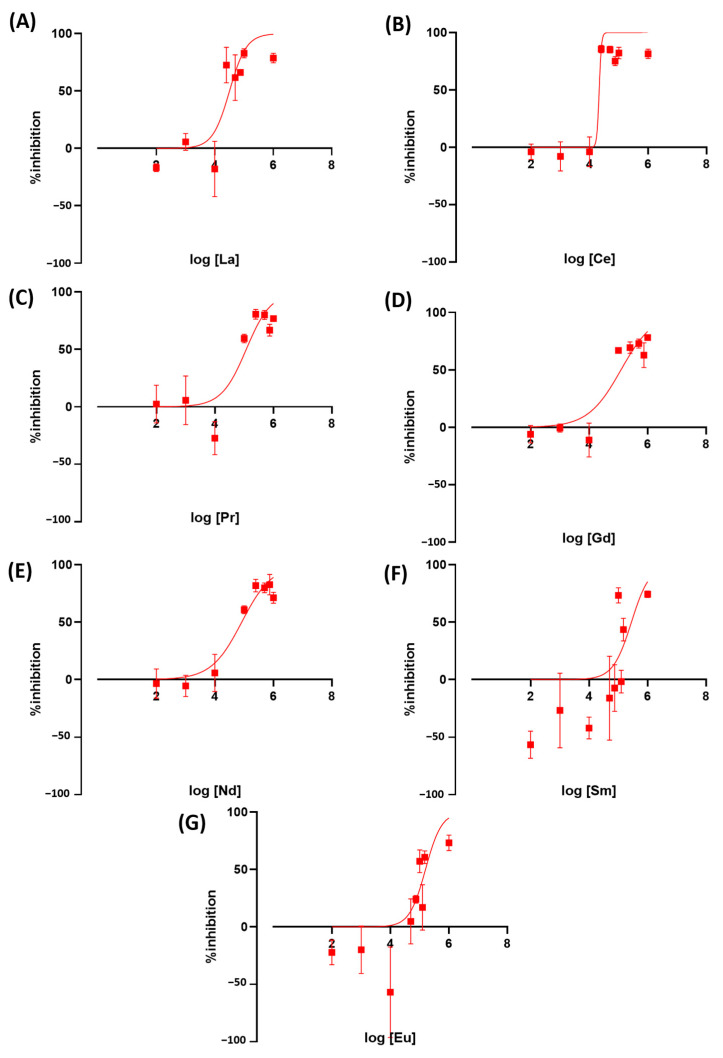
The inhibition rate of *W. globosa* at day seven after exposure to different kinds of LREE; Lanthanum (**A**), Cerium (**B**), Praseodymium (**C**), Gadolinium (**D**), Neodymium (**E**), Samarium (**F**) and Europium (**G**) to determine the median inhibitory concentration (IC_50_), data are presented as Mean ± 95% CI (*n* = 3).

**Figure 7:**
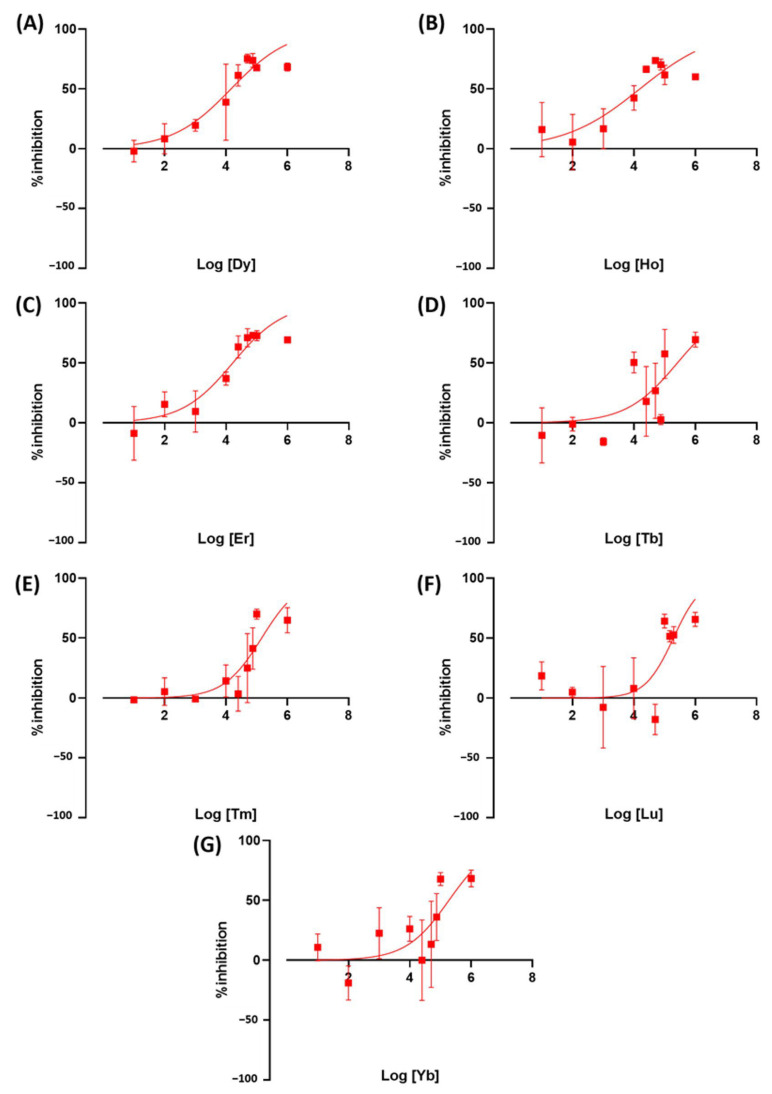
The inhibition rate of *W. globosa* at day seven after exposure to different kinds of HREE; Dysprosium (**A**), Holmium (**B**), Erbium (**C**), Terbium (**D**), Thulium (**E**), Lutetium (**F**) and Ytterbium (**G**) to determine the median inhibitory concentration (IC_50_), data are presented as Mean ± 95% CI (*n* = 3).

We tested the toxicity of rare earth elements on duckweed as a newly emerging contaminant. Exposure to REE resulted in an altered *Wolffia* growth rate. Ce was the most toxic LREE at IC_50_ = 21.3 ppm, followed by La at 31.9 ppm. Both metals are comparable to or more toxic than the herbicides we used as a positive control in this study. The other metals, Pr, Gd, Nd, Sm, and Eu, showed to be less toxic with IC_50_ values at 117.8, 122.4, 82.68, 279.4, and 158.2 ppm, respectively (Figure 6). These results showed that there is a very high variation in toxicity between LREE. HREE results showed Dy, Ho, and Er to be more toxic than other REEs and herbicides used in this study. The IC_50_ value of these REEs is 14.6, 15.5, and 16.5, respectively. The other HREE showed similar toxicity to less toxic LREEs. Tb, Tm, Lu, and Yb have IC_50_ values of 260.3, 146.3, 195.7, and 187.9 (Figure 7).

**Figure 8:**
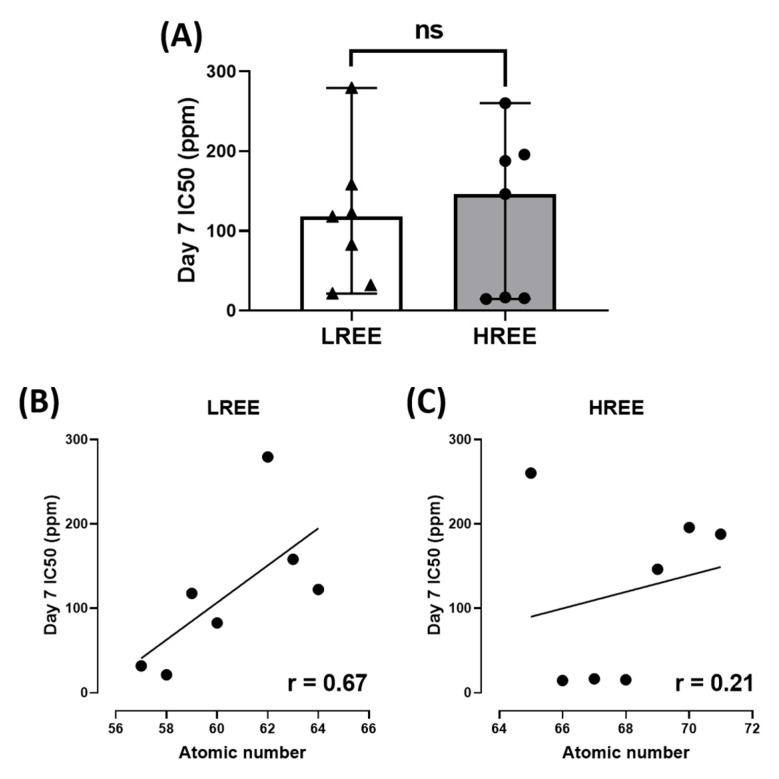
Comparison of overall *W. globosa* Day 7 IC_50_ between LREE and HREE, data are calculated using unpaired *t*-test and presented as Median ± 95% CI (ns = not significant difference) (**A**). Linear regression analysis showing the relation between atomic numbers to their Day 7 IC_50_ for LREE with r = 0.67 (**B**) and HREE with r = 0.21 (**C**).

**Figure 9:**
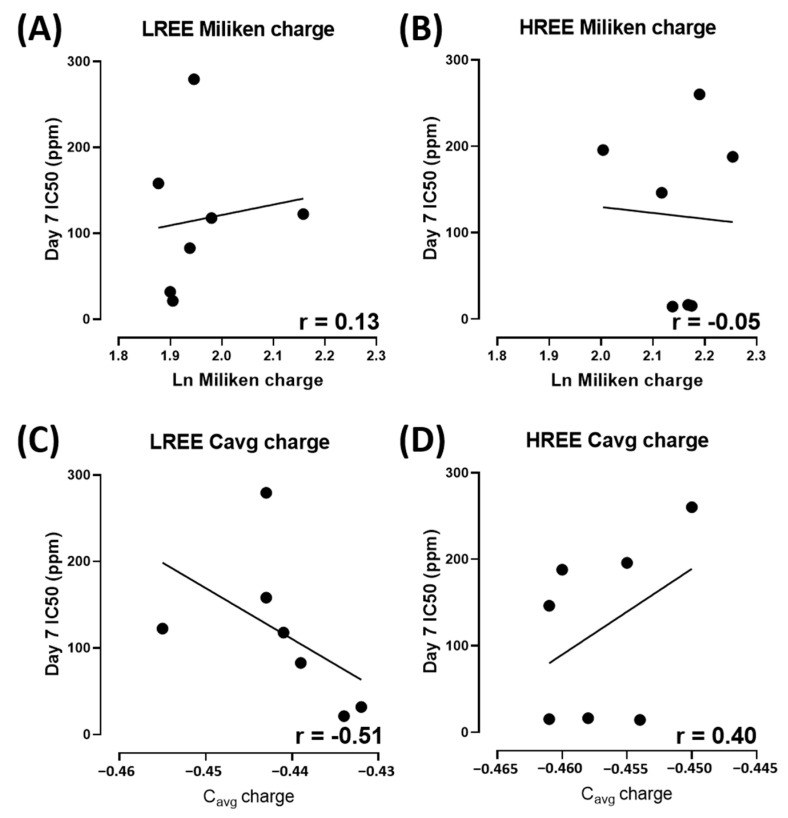
Linear regression analysis on the relation between Ln Milliken charge to Day 7 IC_50_ for LREE (**A**) and HREE (**B**) and Linear regression analysis on the relation between C_avg_ charge to Day 7 IC_50_ for LREE (**C**) and HREE (**D**) Overall Day 7 IC_50_ comparison between LREE and HREE, data are calculated using unpaired *t*-test and presented as Median ± 95% CI.

In addition, we also observed the overall toxicity between groups and their IC_50_ with their respective atomic number. The overall toxicity between LREE and HREE is similar (Figure 8A). The linear regression analysis suggests that Day 7 IC_50_ of LREE has a moderate correlation to their atomic number with r = 0.67 compared to HREE, which has a very weak correlation with r = 0.21. We also tested the relation between Day 7 IC_50_ of LREE and HREE to Lanthanides (Ln) Milliken as well as Aromatic C_avg_ charges. The Ln Milliken and Aromatic C_avg_ charge values were obtained from the previous study (Table 2) [38]. The result showed no correlation between the Ln Milliken charge to the Day 7 IC_50_ value for both LREE (r = 0.13) and HREE (r = −0.05). On the other hand, the Aromatic C_avg_ charge result showed a moderate negative correlation for LREE (r = −0.51) and a weak positive correlation for HREE (r = 0.40) (Figure 9).

## 4. Discussion

In this study, we utilized StarDist for *Wolffia globosa* frond counting. The idea of using StarDist came from our previous study to count *Tetrahymena* cells with a circular shape [35]. The result of *Wolffia* detection using StarDist showed promising results, as presented in Figure 2, with several misdetections due to light reflections on some parts of the microplate and reflection of *Wolffia*. However, the number of misdetections is small compared to the number of *Wolffia* fronds, which means the counting result might not be affected significantly. Therefore, the counting result can be used reliably. We also implemented a Python-based script in our workflow to automate the Excel calculation process, which reduces the workload by around ~50% compared to manual operation.

In order to further test the performance of the proposed *Wolffia* counting method using StarDist, herbicides such as Glyphosate and Glufosinate were used. Glyphosate is an herbicide commonly used in aquatic environments. Its active compound, glyphosate, inhibits 3-enolpyruvylshikimic acid 5-phosphate synthase (EPSPS), an essential enzyme in Aromatic amino acids and compounds biosynthesis [41]. Glufosinate is a broad-spectrum herbicide used to target glyphosate-resistant weeds. Exposure to Glufosinate increases reactive oxygen species levels in plants due to the disruption of photorespiration and light reactions of photosynthesis. In addition, it also inhibits glutamine synthetase (GS), which has a vital role in ammonia detoxification and amino acid metabolism in plants [42]. Exposing *Wolffia* to herbicides resulted in growth inhibition and bleaching of *Wolffia* fronds. A similar result was also observed in *Lemna minor*, another species of duckweed that the OECD has recognized as a plant model for water toxicity tests. The study showed dry weight loss and chlorophyll in *L. minor* fronds after seven-day exposure to glyphosate [43]. A similar result was also reported in several other plants [44,45,46]. In order to identify fronds chlorosis, we performed a manual observation of the plants before recording. This is due to the limitation of the recording contraption, which only records the video in black and white.

The result of REE exposure showed varied results. Several REEs showed higher toxicity compared to those of herbicides, namely Dysprosium, Holmium, and Erbium are the most toxic REEs on duckweed, followed by Cerium, then Lanthanum, which has comparable toxicity to the herbicides (Table 2). Most REE toxicity has not been specified. However, previous studies suggest the toxicity was due to the release of free metal ions [47], which might cause chlorosis [48].

There needs to be more information regarding REEs toxicity in aquatic animal models except for La, Ce, and Gd [49]. Previous studies reported REEs as an analog to calcium. The abundance of REEs or calcium inhibits potassium uptake for a short time, which causes chlorosis. This property causes REEs to replace Ca from many enzymes, which interfere with the physiological functions of Ca. In addition to being a competitor in enzymatic functions, La^3+^, the most common REE to be investigated, was known to be a potent Ca^2+^ transport inhibitor, causing deficiency due to a dysfunctional cytoplasm membrane.

The result of our study also indicates a different toxicity profile compared to a previous comprehensive study of REE on zebrafish embryos. While a previous study on zebrafish embryos suggested LREE to be more toxic than HREE [38], there seems to be no statistical difference between the toxicity of LREE and HREE on *W. globosa* (Figure 8A). There is a moderate correlation between their atomic number to Day 7 IC_50_ for LREE (r = 0.67) and a very weak correlation for HREE (r = 0.21) (Figure 8B,C). However, there is no correlation between the Ln Milliken charge to both LREE (r = 0.13) and HREE (r = −0.05). Aromatic C_avg_ charge showed a moderate negative correlation to Day 7 IC_50_ for LREE (r = −0.51) and a weak correlation for HREE (r = 0.40) (Figure 9). These results further support the dissimilarity between the results of the previous study on zebrafish embryos and this study on *W. globosa*. The order of toxicity from the most toxic to least toxic on *W. globosa* is Dysprosium > Erbium > Holmium > Cerium > Lanthanum > Glufosinate > Glyphosate > Neodymium > Praseodymium > Gadolinium > Thulium > Europium > Ytterbium > Lutetium > Terbium > Samarium. This result showed the potential of *W. globosa* to have a high tolerance to REE, which was also observed in heavy metals and their high potential in absorbing those metals [22].

## 5. Conclusions

In conclusion, we can provide a counting tool for duckweed fronds using StarDist with respectable performance. We also tested the performance of our tool on the inhibitory property of chemicals of emerging concerns which are rare earth elements. The result of rare earth elements exposure showed Dysprosium, Erbium, Holmium, Lanthanum, and Cerium to be the more toxic of the 14 rare earth elements that we tested with IC_50_ values at 14.6, 15.5, 16.5, 21.3, and 31.9 ppm respectively, which showed higher toxicity compared to two kinds of herbicides, Glufosinate and Glyphosate with IC_50_ values of 34.0 and 36.4 ppm respectively. In addition to the IC_50_ test result, there also seems to only be a weak to medium correlation between the atomic number, Ln Milliken charge, and C_avg_ charge to the IC_50_ values that we had obtained. Through this result, we hope to increase the limited knowledge and awareness of REE toxicity. Therefore, through this study, we hope we can provide a free-to-use and automatic alternative tool for future aquatic toxicology studies using *Wolffia globosa* frond-counting while highlighting REE toxicity on aquatic plant models.

## Figures and Tables

**Table 1:** Comparison of available Duckweed fronds counting tool/method.

Plant Species	Endpoints	Manual/Auto	Software and References
*Wolffia globosa*	Duckweed fronds counting based on the size and shape of objects	Automatic	QuPath (pre-training) and StarDist (Training and processing) (This study)
*Wolffia globosa*	EC_50_ determination through biomass weight	Manual	None [29]
*Lemna gibba*	Duckweed frond counting based on image recording and frond area interpretation based on color intensity	Automatic	Nikon ACT-2U (Area measurement) and Assess: Image Analysis Software for Plant Disease Quantification (Frond area interpretation) [24]
*Lemna minor*	Differentiation between live and dead duckweed	Automatic	NI Vision Assistant [27]
*Landoltia punctata*	Manual counting using a magnifying glass	Manual	None [30]
*Lemna minor*	Differentiation between live and dead duckweed and counting	Automatic	ACD-See (pre-processing) and Image Pro-Plus (processing) [31]
*Lemna minor*	Duckweed fronds counting	Manual	None [32]

**Table 2:** Day 7 IC_50_ values of herbicides and rare earth elements, Lanthanide’s charge, and Aromatic C_avg_ charge of all tested rare earth elements.

Compound	Atomic Number	Valence Electron	Group	Day 7 IC_50_ (ppm)	Ln Milliken Charge	Aromatic C_avg_ Charge
Glyphosate	-	*-*	Herbicide	36.4	-	-
Glufosinate	-	-	Herbicide	34.0	-	-
Lanthanum	57	*5d* ^1^ *6s* ^2^	LREE	31.9	1.900	−0.432
Cerium	58	*4f* ^1^ *5d* ^1^ *6s* ^2^	LREE	21.3	1.905	−0.434
Praseodymium	59	*4f* ^3^ *6s* ^2^	LREE	117.8	1.980	−0.441
Neodymium	60	*4f* ^4^ *6s* ^2^	LREE	82.7	1.938	−0.439
Samarium	62	*4f* ^6^ *6s* ^2^	LREE	279.4	1.946	−0.443
Europium	63	*4f* ^7^ *6s* ^2^	LREE	158.2	1.877	−0.443
Gadolinium	64	*4f* ^7^ *5d* ^1^ *6s* ^2^	LREE	122.4	2.158	−0.455
Terbium	65	*4f* ^9^ *6s* ^2^	HREE	260.3	2.190	−0.45
Dysprosium	66	*4f* ^10^ *6s* ^2^	HREE	14.6	2.138	−0.454
Holmium	67	*4f* ^11^ *6s* ^2^	HREE	16.5	2.168	−0.458
Erbium	68	*4f* ^12^ *6s* ^2^	HREE	15.5	2.175	−0.461
Thulium	69	*4f* ^13^ *6s* ^2^	HREE	146.3	2.117	−0.461
Ytterbium	70	*4f* ^14^ *6s* ^2^	HREE	195.8	2.004	−0.455
Lutetium	71	*4f* ^14^ *5d* ^1^ *6s* ^2^	HREE	187.9	2.254	−0.460

LREE = Light rare earth element; HREE = Heavy rare earth element. Ln Milliken charge: the calculated Milliken charge of Lanthanide when Lanthanide forms the model complex [38]. Aromatic C_avg_ charge: The average Milliken charge of the carbons on the Aromatic ring in the Ln complex. “Reprinted/adapted with permission from Ref. [38]. 2022, Lin, YT”.

## Data Availability

Data will be made available on request.

## Supplementary Materials

The following supporting information can be downloaded at: https://www.mdpi.com/article/10.3390/toxics11080680/s1, File S1: InhibitionAnalyzer1.5.zip, File S2: TF_SavedModel_small_duckweed_LoRes.zip, File S3: StarDist SOP.pptx.
