# Supplementary material for: Deep Learning-Based Automatic Duckweed Counting Using StarDist and Its Application on Measuring Growth Inhibition Potential of Rare Earth Elements as Contaminants of Emerging Concerns
Source: Toxics. 2023 Aug 8;11(8):680. doi: 10.3390/toxics11080680 (PMC10457735; doi:10.3390/toxics11080680)
Supplement: Supplementary file 1 [file toxics-11-00680-s001.zip › File S3. StarDist SOP.pptx]

## Slide 1
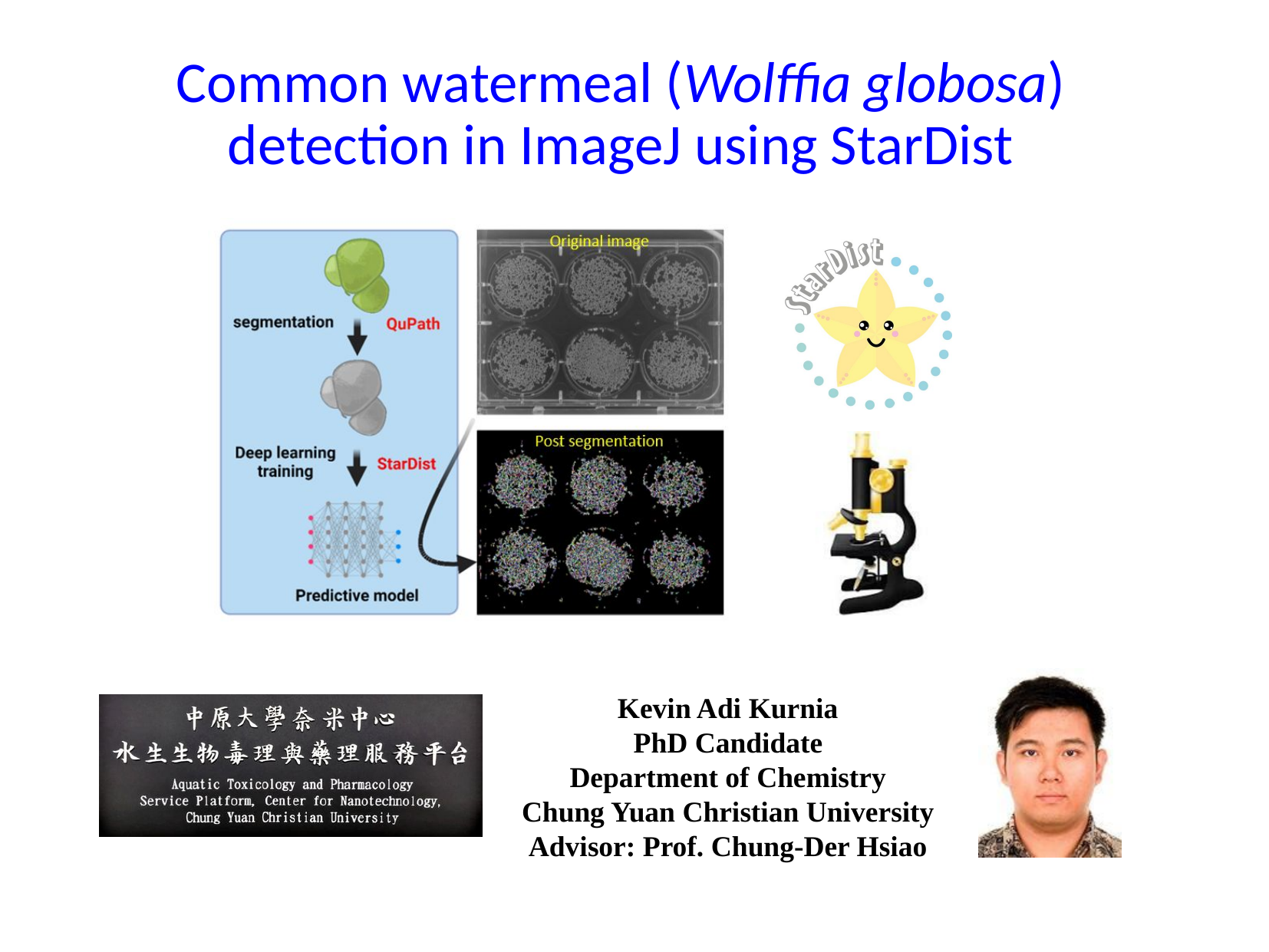

# Common watermeal (Wolffia globosa) detection in ImageJ using StarDist
Kevin Adi Kurnia
PhD Candidate
Department of Chemistry
Chung Yuan Christian University
Advisor: Prof. Chung-Der Hsiao

## Slide 2
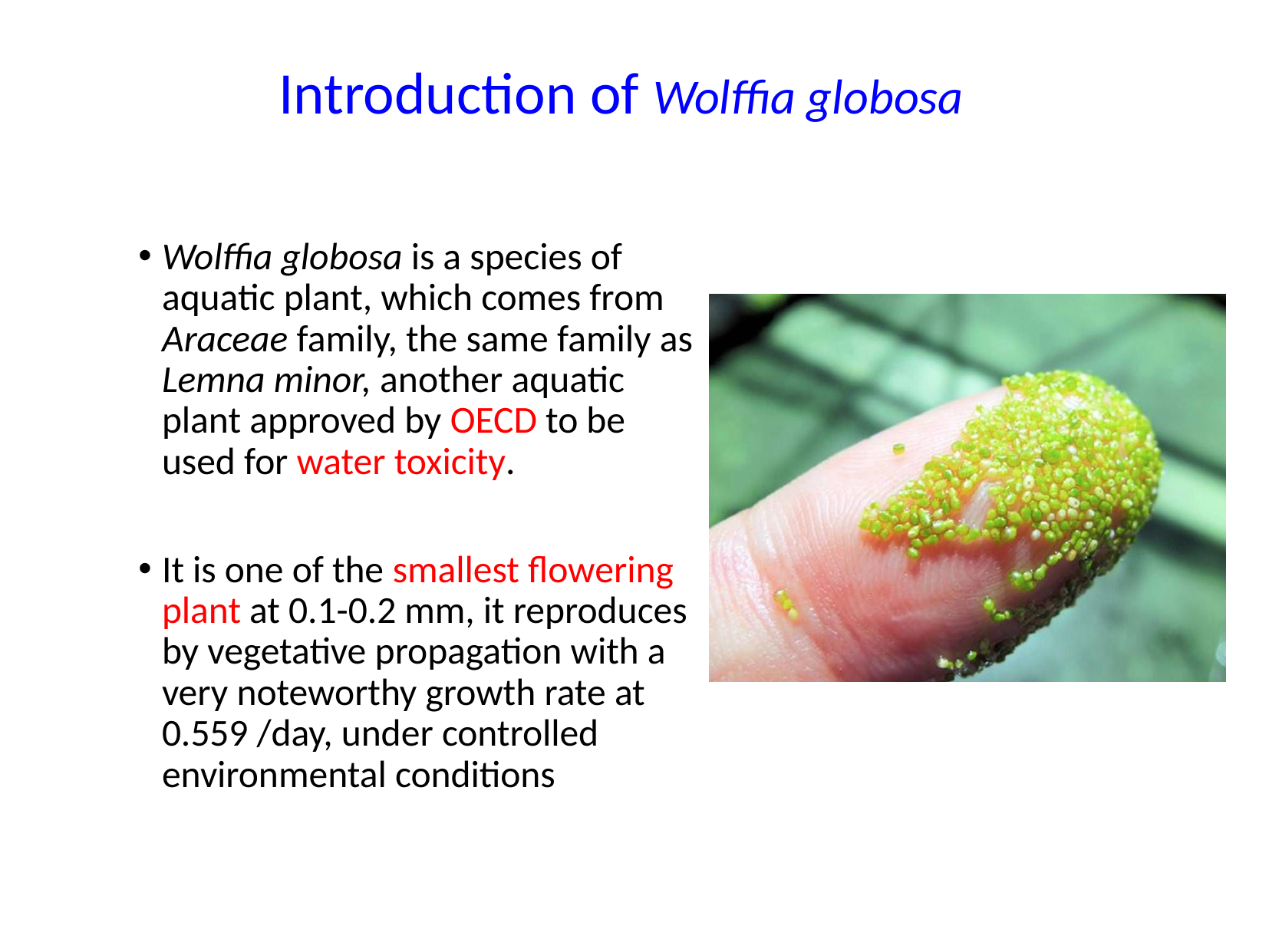

# Introduction of Wolffia globosa
Wolffia globosa is a species of aquatic plant, which comes from Araceae family, the same family as Lemna minor, another aquatic plant approved by OECD to be used for water toxicity.
It is one of the smallest flowering plant at 0.1-0.2 mm, it reproduces by vegetative propagation with a very noteworthy growth rate at 0.559 /day, under controlled environmental conditions

## Slide 3
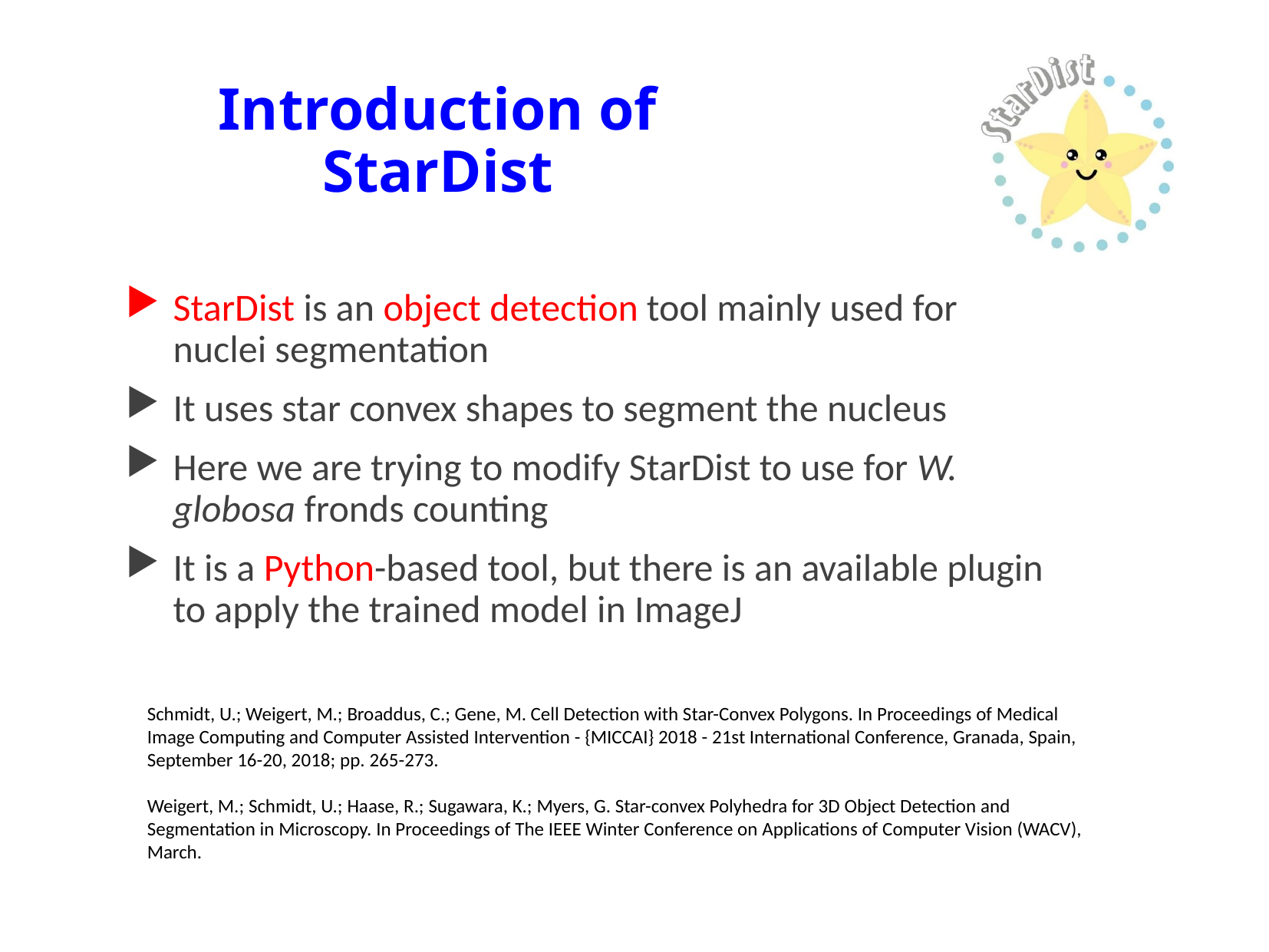

# Introduction of StarDist
StarDist is an object detection tool mainly used for nuclei segmentation
It uses star convex shapes to segment the nucleus
Here we are trying to modify StarDist to use for W. globosa fronds counting
It is a Python-based tool, but there is an available plugin to apply the trained model in ImageJ
Schmidt, U.; Weigert, M.; Broaddus, C.; Gene, M. Cell Detection with Star-Convex Polygons. In Proceedings of Medical Image Computing and Computer Assisted Intervention - {MICCAI} 2018 - 21st International Conference, Granada, Spain, September 16-20, 2018; pp. 265-273.
Weigert, M.; Schmidt, U.; Haase, R.; Sugawara, K.; Myers, G. Star-convex Polyhedra for 3D Object Detection and Segmentation in Microscopy. In Proceedings of The IEEE Winter Conference on Applications of Computer Vision (WACV), March.

## Slide 4
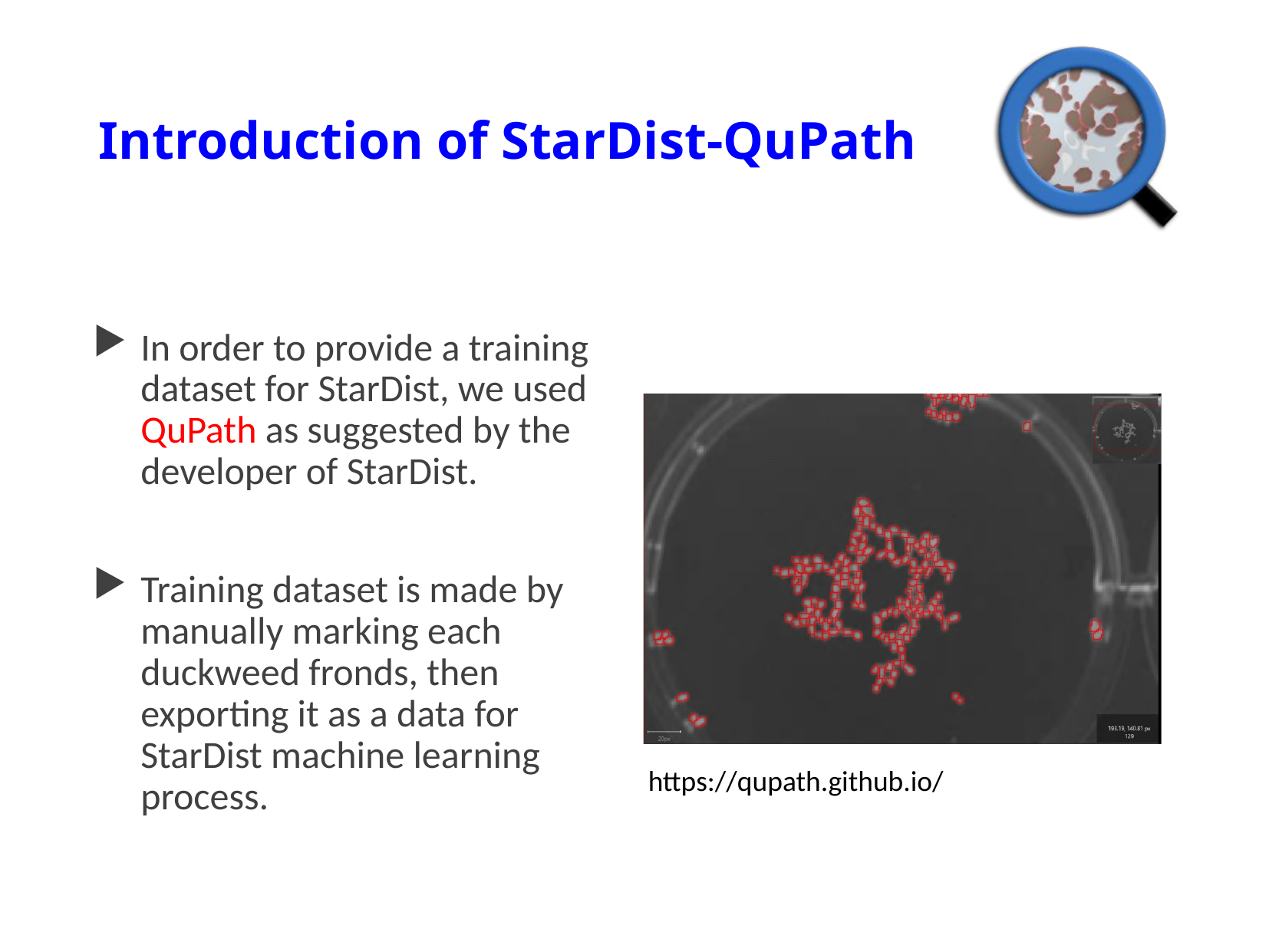

# Introduction of StarDist-QuPath
In order to provide a training dataset for StarDist, we used QuPath as suggested by the developer of StarDist.
Training dataset is made by manually marking each duckweed fronds, then exporting it as a data for StarDist machine learning process.
https://qupath.github.io/

## Slide 5
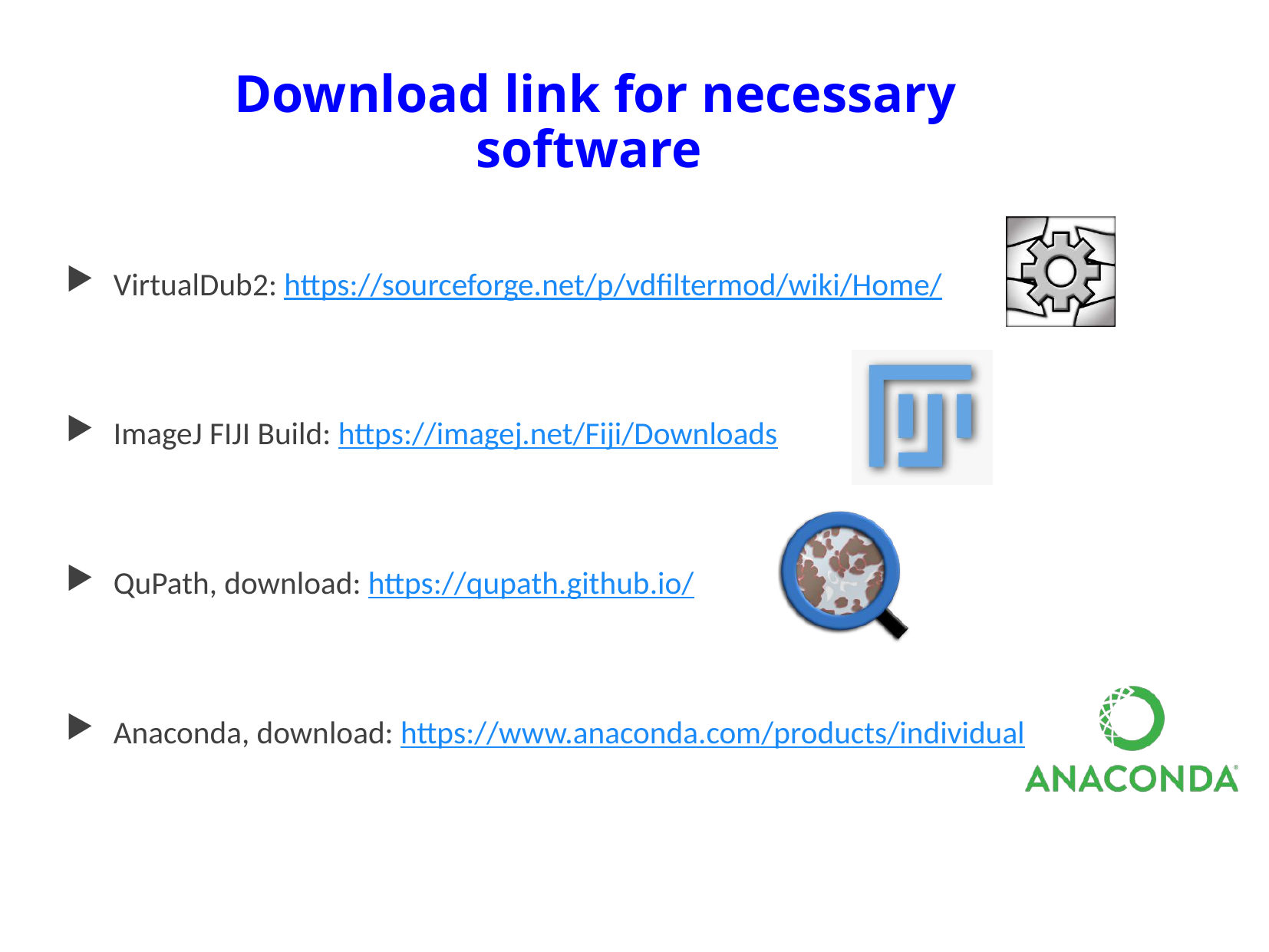

# Download link for necessary software
VirtualDub2: https://sourceforge.net/p/vdfiltermod/wiki/Home/
ImageJ FIJI Build: https://imagej.net/Fiji/Downloads
QuPath, download: https://qupath.github.io/
Anaconda, download: https://www.anaconda.com/products/individual

## Slide 6
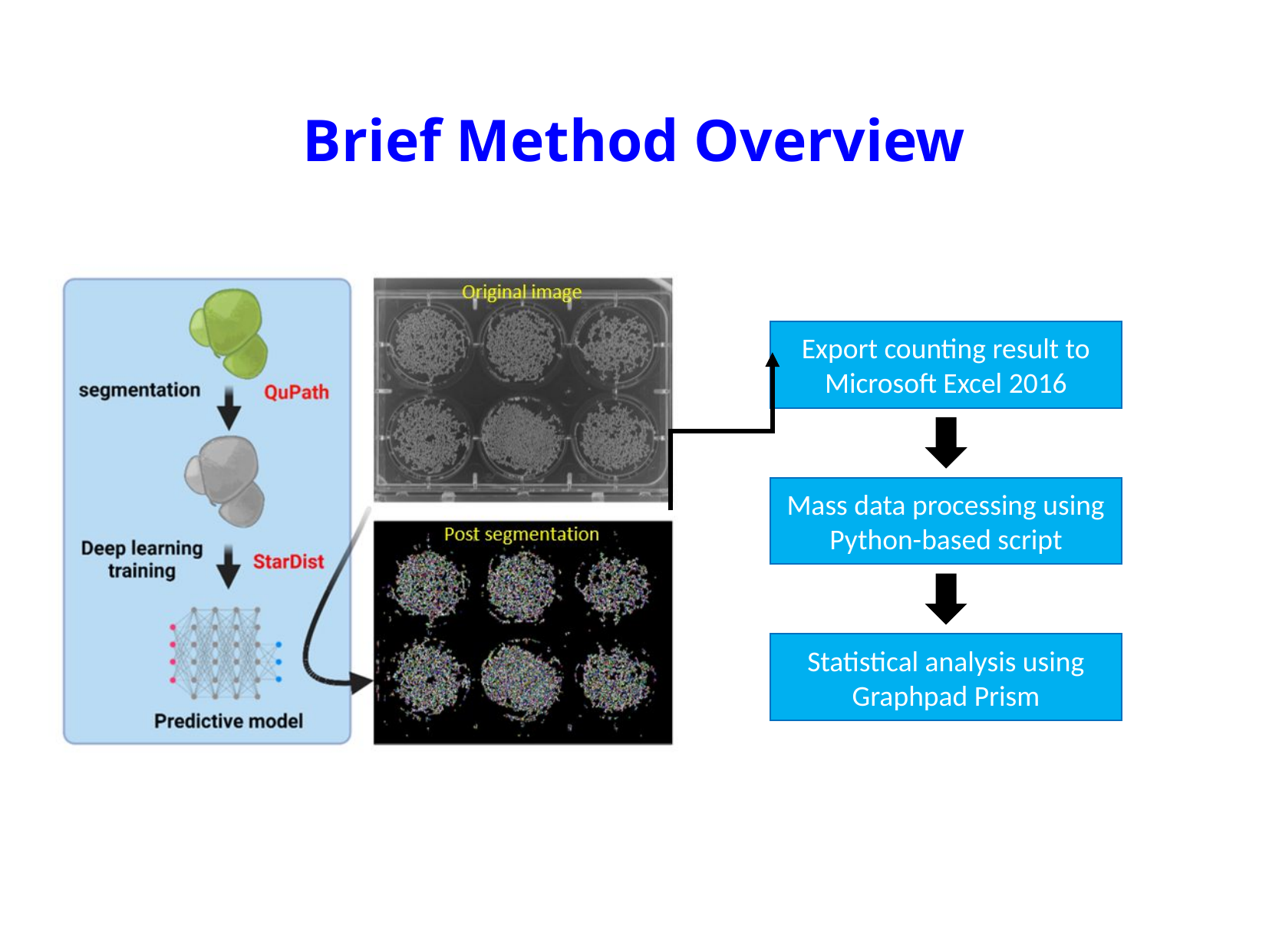

# Brief Method Overview
Export counting result to Microsoft Excel 2016
Mass data processing using Python-based script
Statistical analysis using Graphpad Prism

## Slide 7
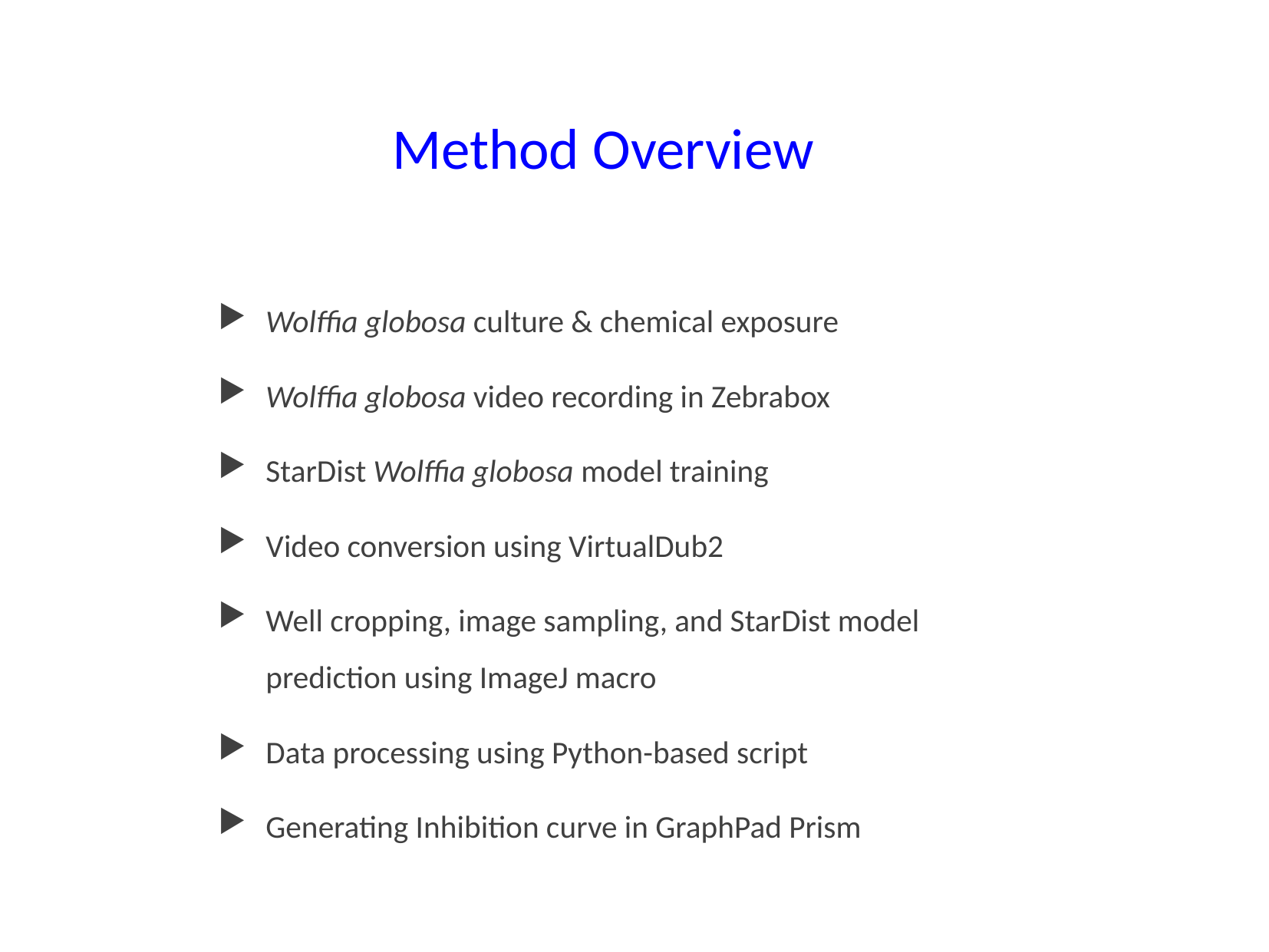

# Method Overview
Wolffia globosa culture & chemical exposure
Wolffia globosa video recording in Zebrabox
StarDist Wolffia globosa model training
Video conversion using VirtualDub2
Well cropping, image sampling, and StarDist model prediction using ImageJ macro
Data processing using Python-based script
Generating Inhibition curve in GraphPad Prism

## Slide 8
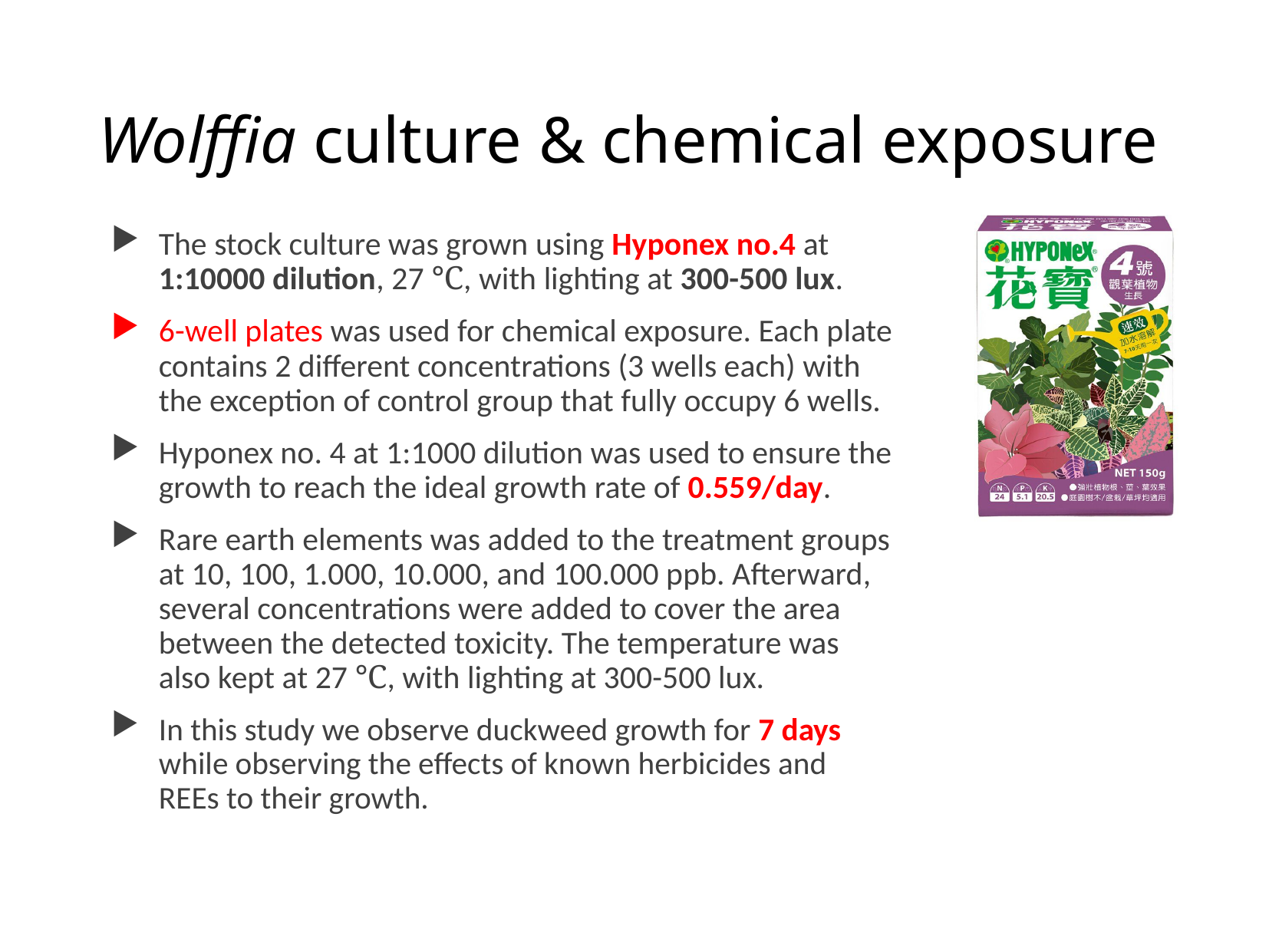

# Wolffia culture & chemical exposure
The stock culture was grown using Hyponex no.4 at 1:10000 dilution, 27 ℃, with lighting at 300-500 lux.
6-well plates was used for chemical exposure. Each plate contains 2 different concentrations (3 wells each) with the exception of control group that fully occupy 6 wells.
Hyponex no. 4 at 1:1000 dilution was used to ensure the growth to reach the ideal growth rate of 0.559/day.
Rare earth elements was added to the treatment groups at 10, 100, 1.000, 10.000, and 100.000 ppb. Afterward, several concentrations were added to cover the area between the detected toxicity. The temperature was also kept at 27 ℃, with lighting at 300-500 lux.
In this study we observe duckweed growth for 7 days while observing the effects of known herbicides and REEs to their growth.

## Slide 9
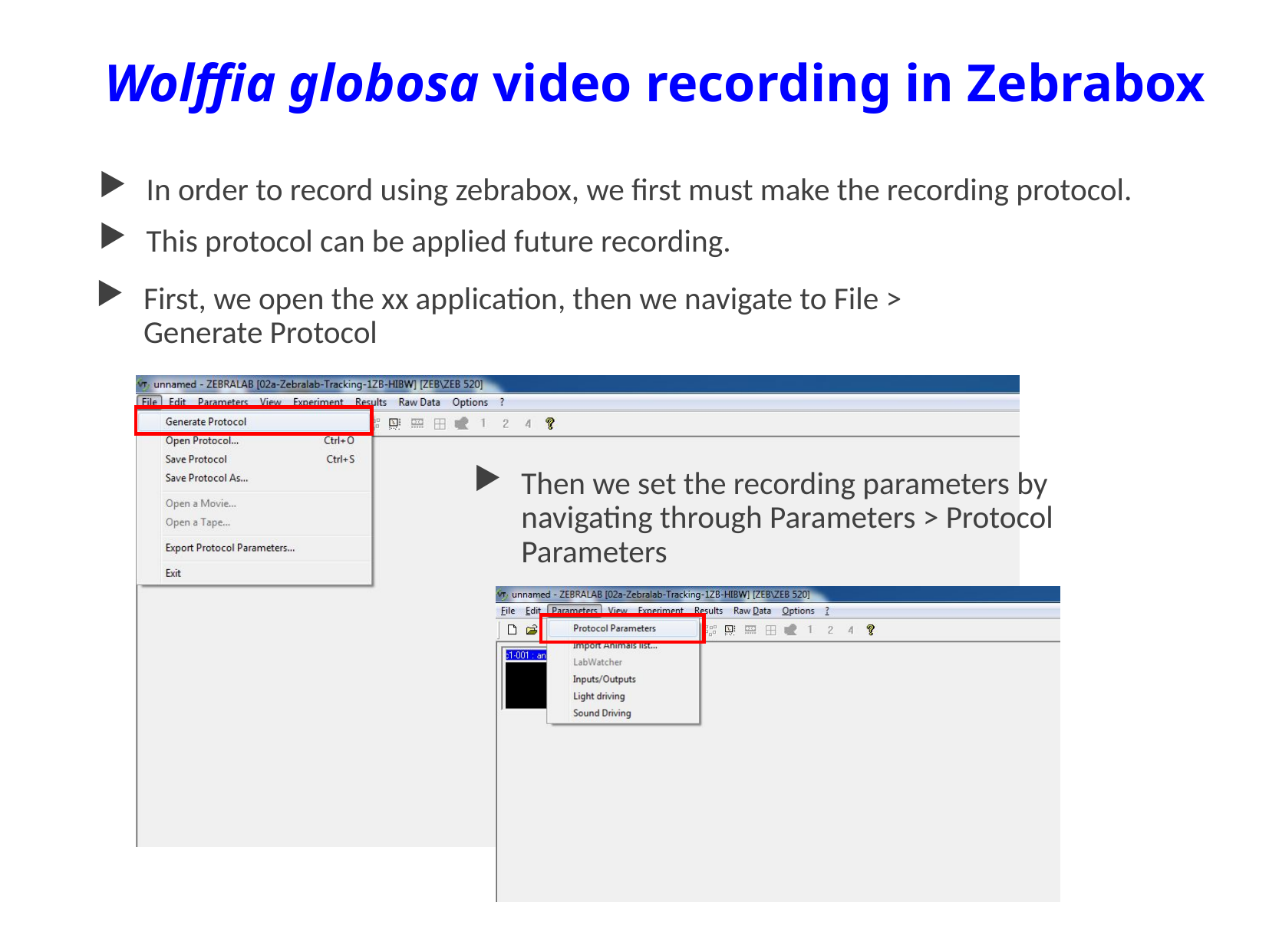

# Wolffia globosa video recording in Zebrabox
In order to record using zebrabox, we first must make the recording protocol.
This protocol can be applied future recording.
First, we open the xx application, then we navigate to File > Generate Protocol
Then we set the recording parameters by navigating through Parameters > Protocol Parameters

## Slide 10
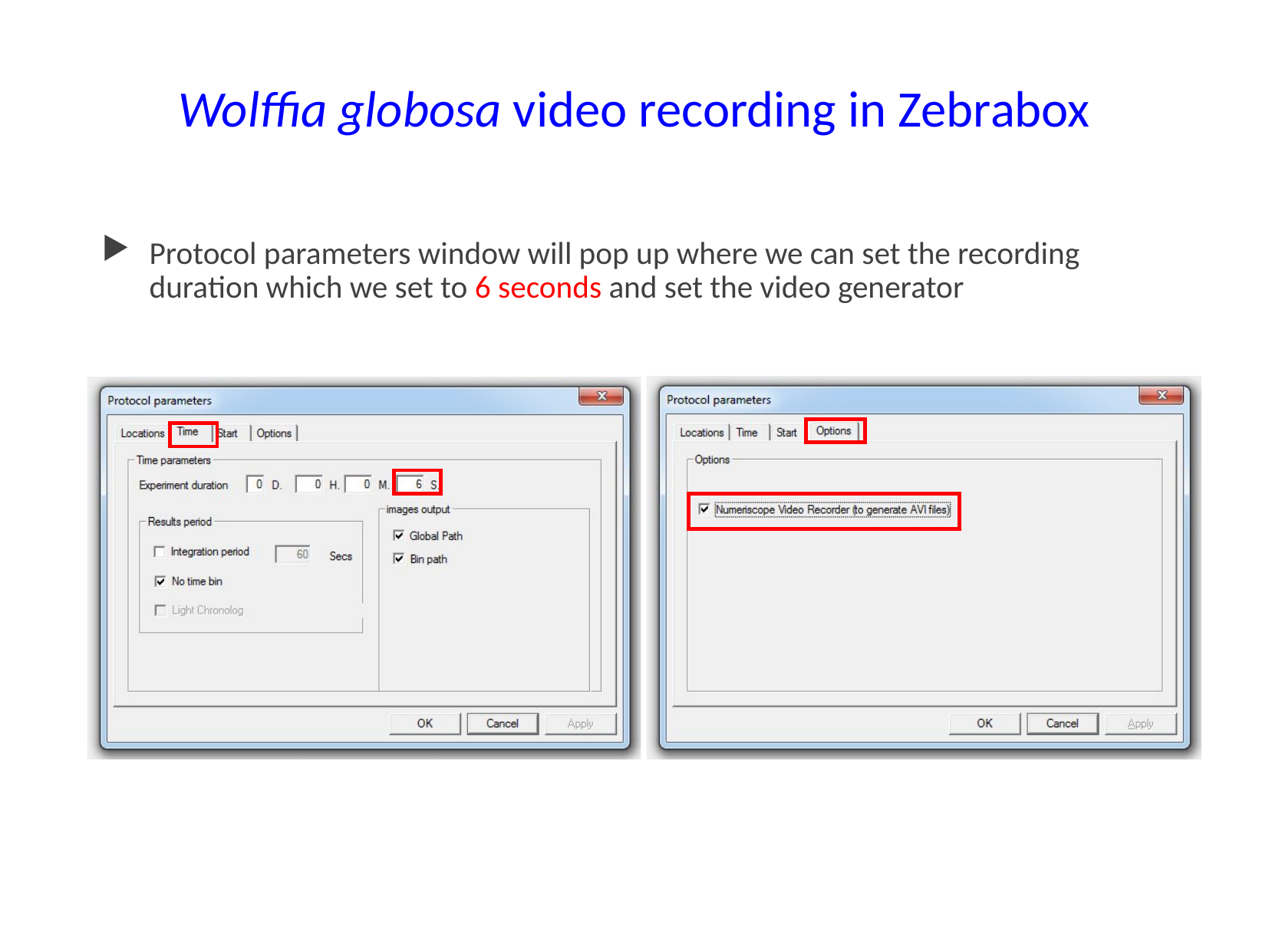

# Wolffia globosa video recording in Zebrabox
Protocol parameters window will pop up where we can set the recording duration which we set to 6 seconds and set the video generator

## Slide 11
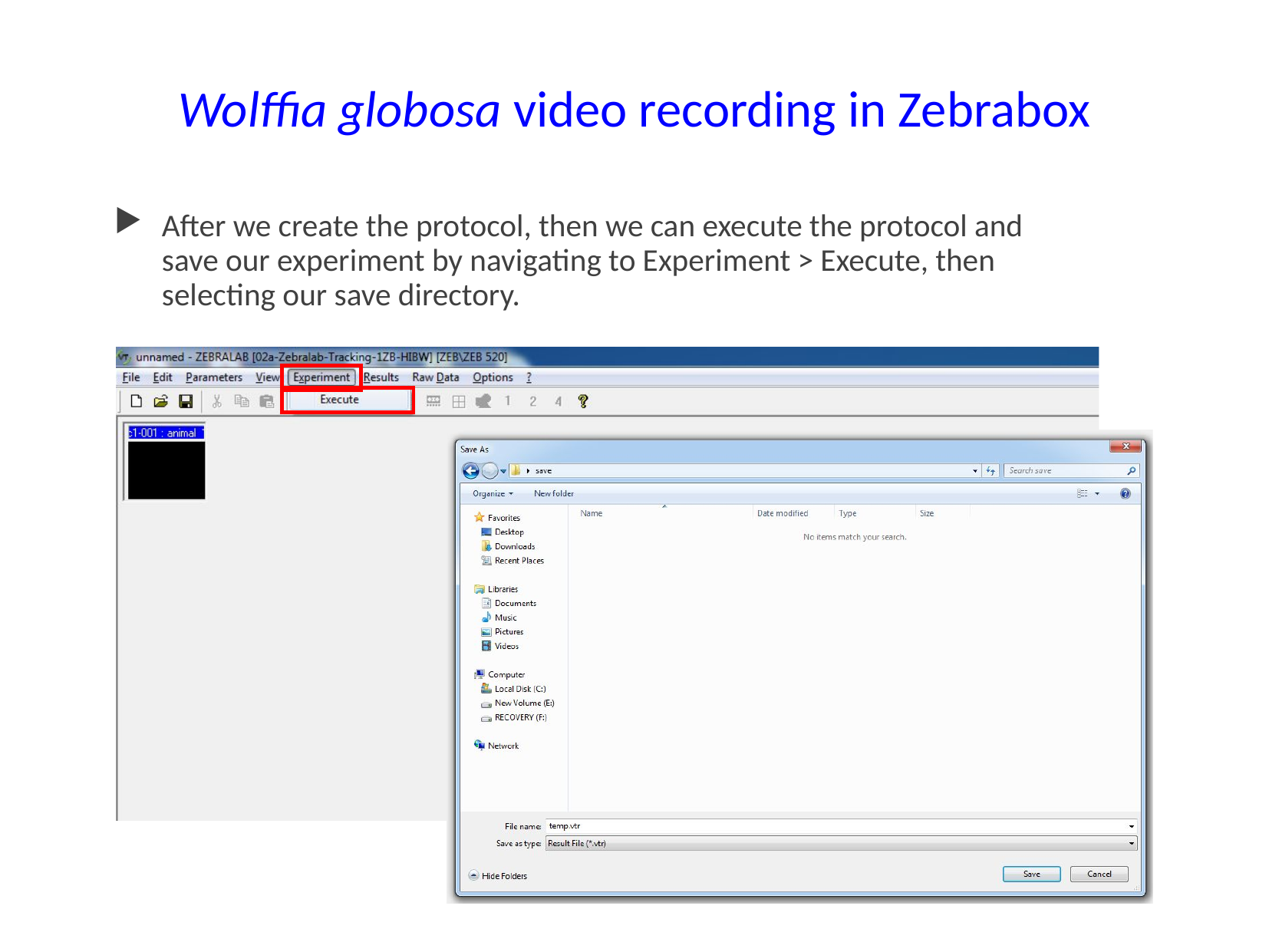

Wolffia globosa video recording in Zebrabox
After we create the protocol, then we can execute the protocol and save our experiment by navigating to Experiment > Execute, then selecting our save directory.

## Slide 12
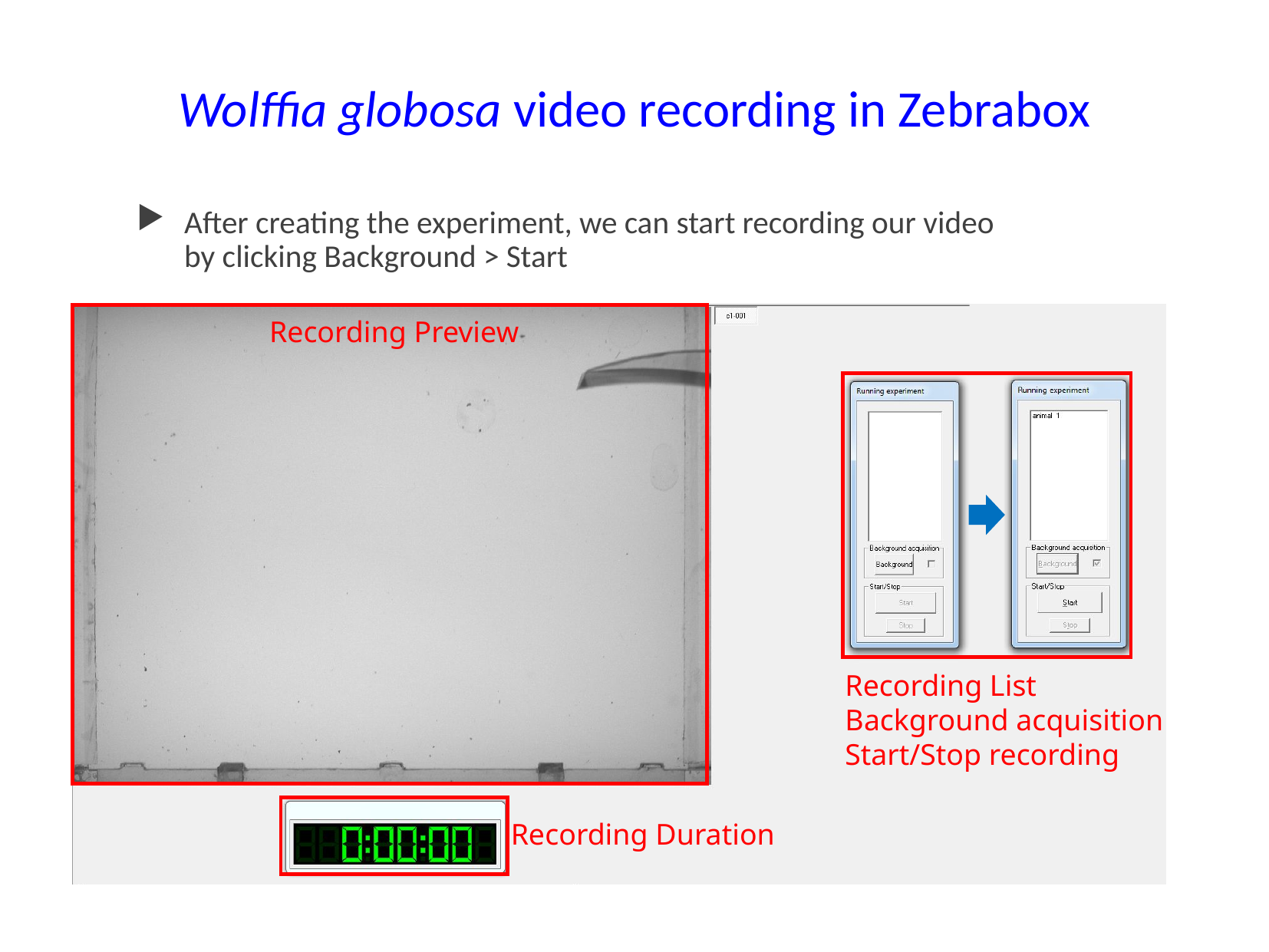

Wolffia globosa video recording in Zebrabox
After creating the experiment, we can start recording our video by clicking Background > Start
Recording Preview
Recording List
Background acquisition
Start/Stop recording
Recording Duration

## Slide 13
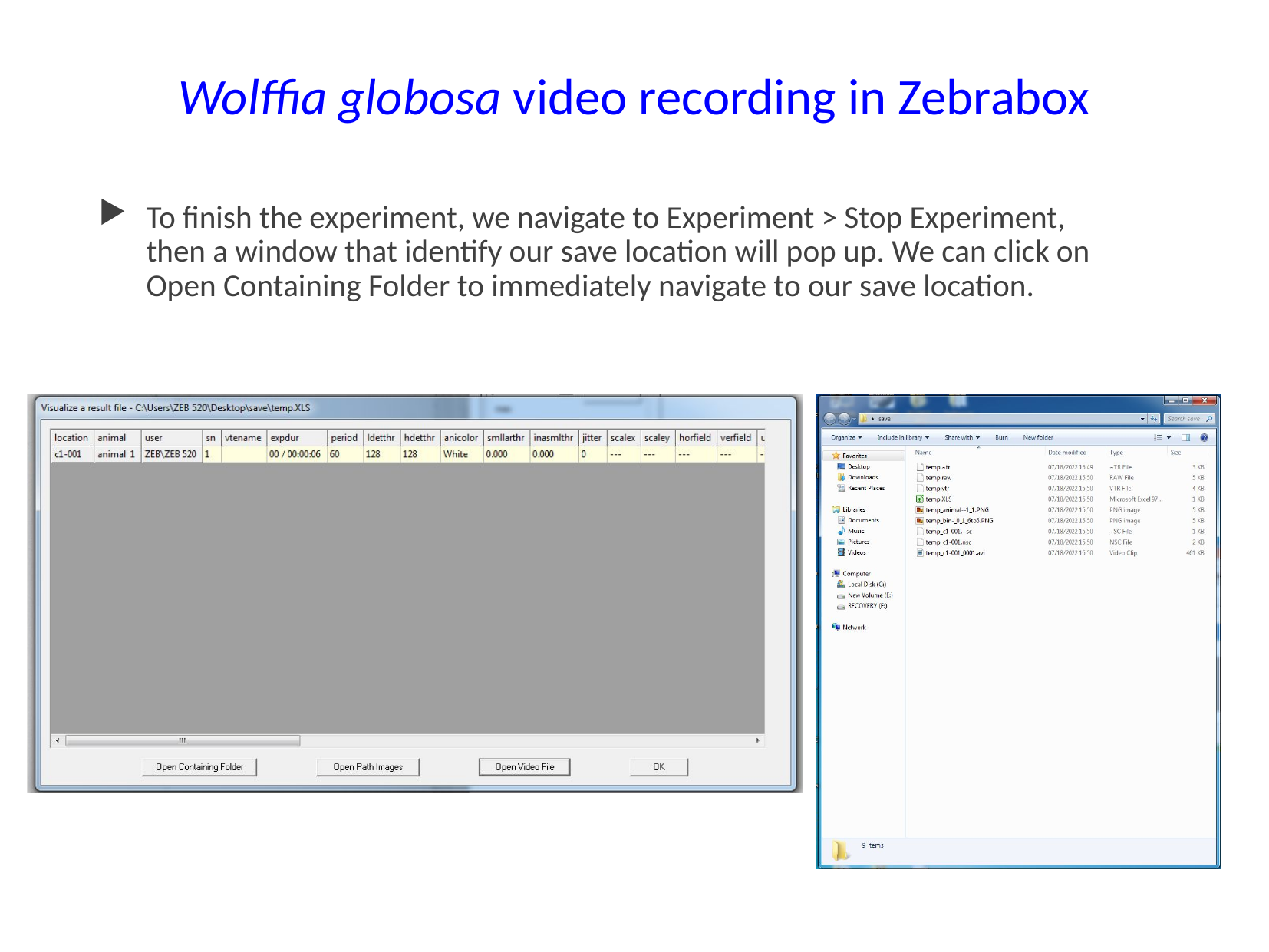

# Wolffia globosa video recording in Zebrabox
To finish the experiment, we navigate to Experiment > Stop Experiment, then a window that identify our save location will pop up. We can click on Open Containing Folder to immediately navigate to our save location.

## Slide 14
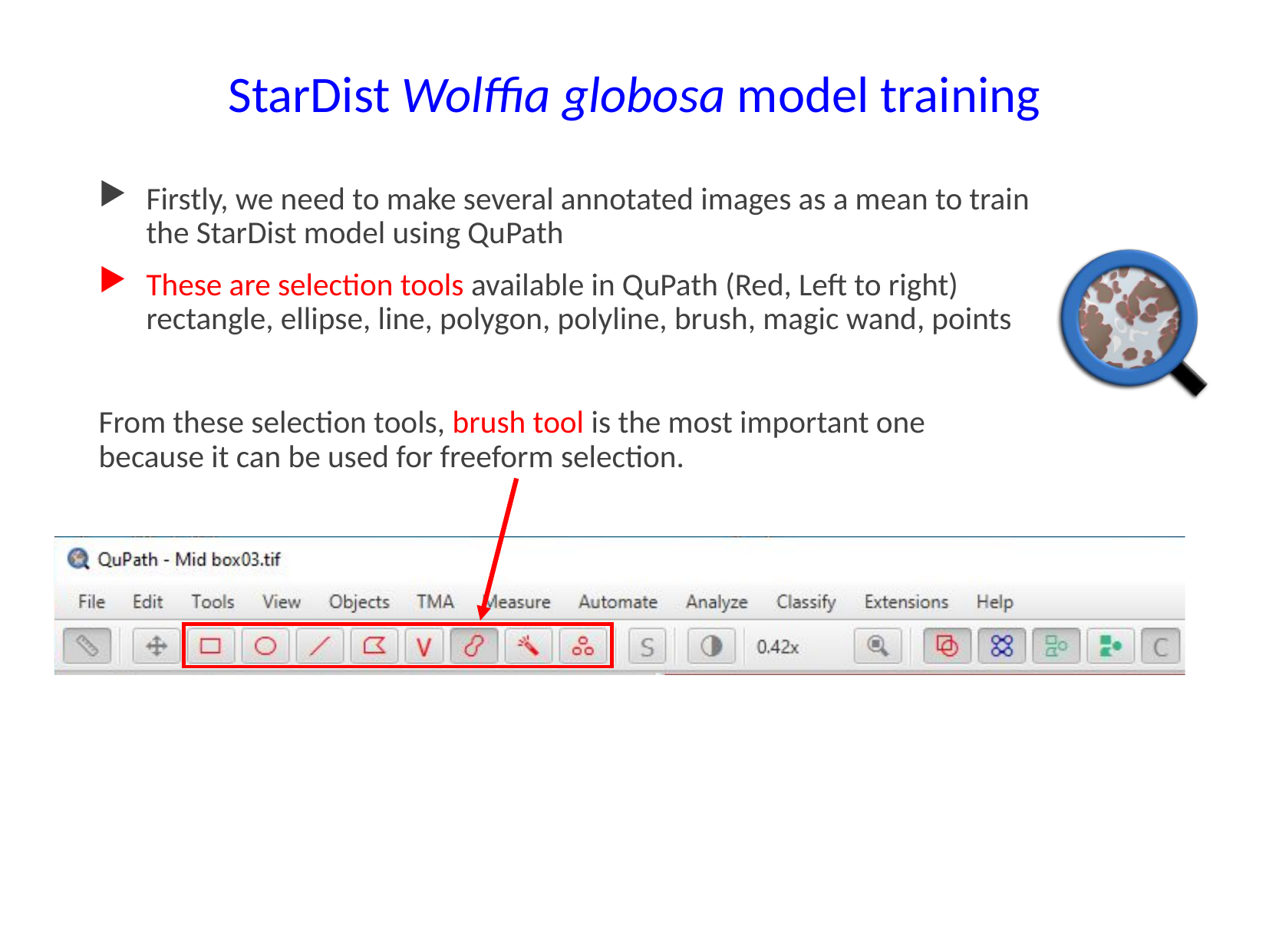

# StarDist Wolffia globosa model training
Firstly, we need to make several annotated images as a mean to train the StarDist model using QuPath
These are selection tools available in QuPath (Red, Left to right) rectangle, ellipse, line, polygon, polyline, brush, magic wand, points
From these selection tools, brush tool is the most important one because it can be used for freeform selection.

## Slide 15
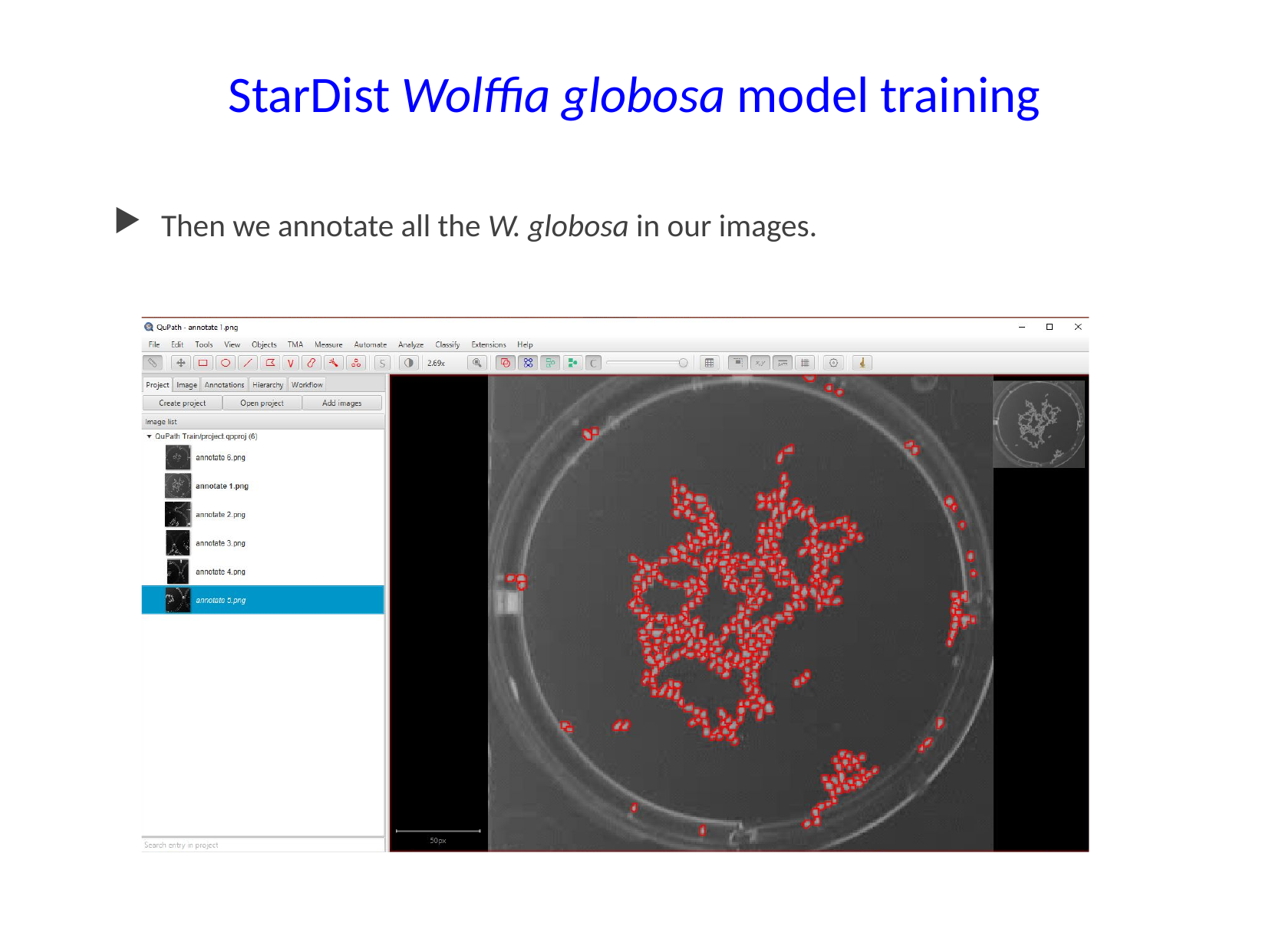

StarDist Wolffia globosa model training
Then we annotate all the W. globosa in our images.

## Slide 16
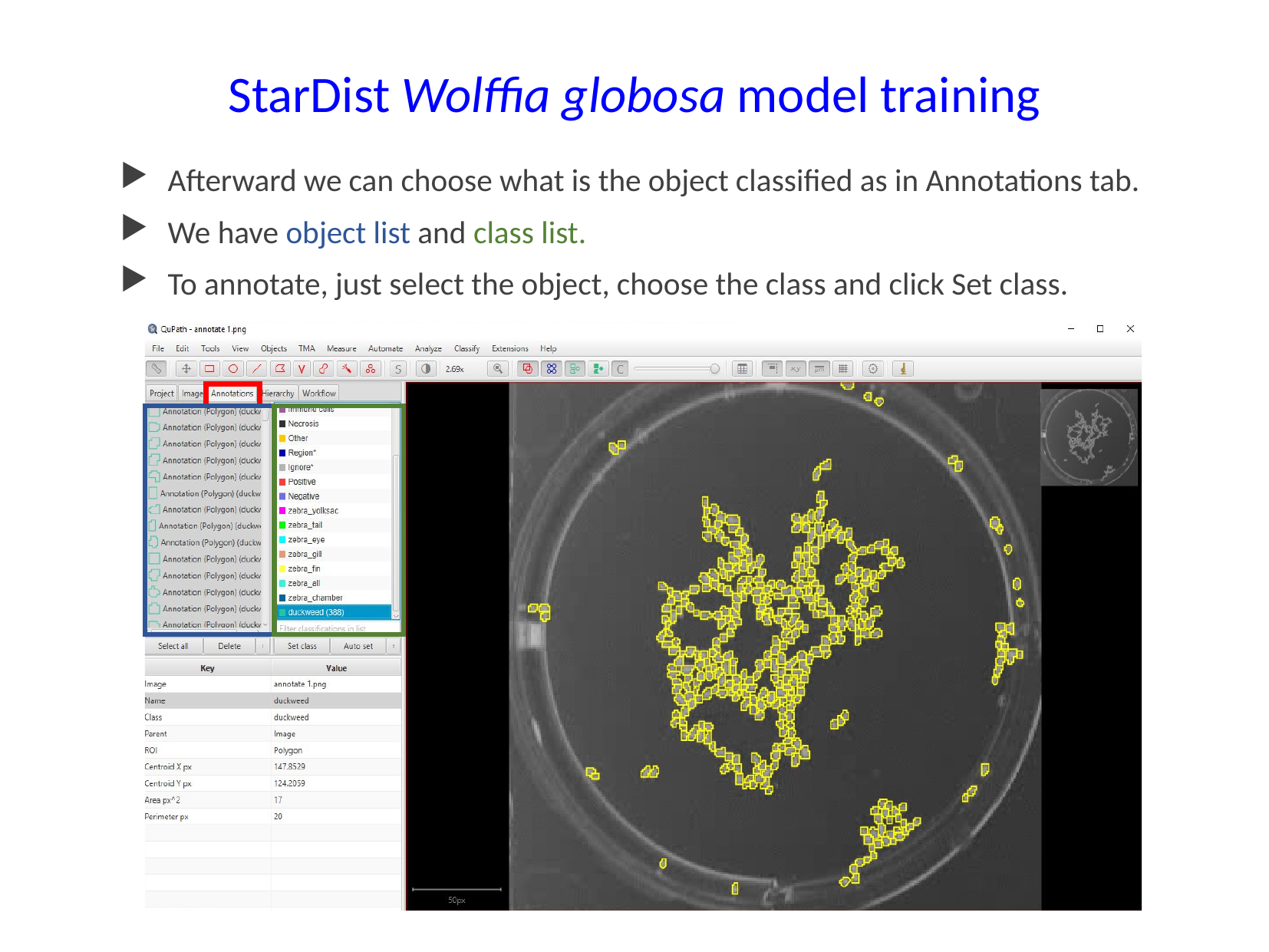

StarDist Wolffia globosa model training
Afterward we can choose what is the object classified as in Annotations tab.
We have object list and class list.
To annotate, just select the object, choose the class and click Set class.

## Slide 17
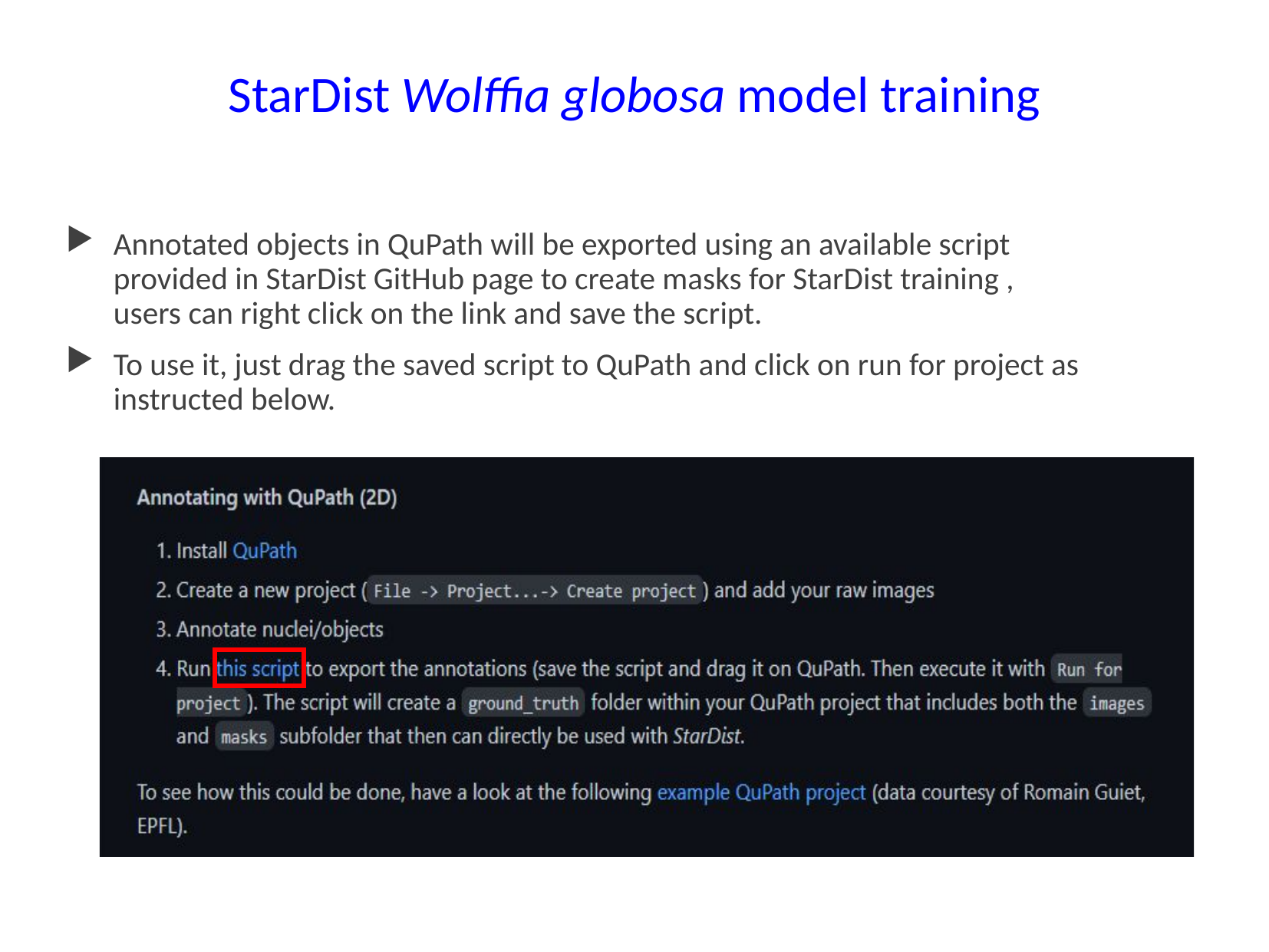

StarDist Wolffia globosa model training
Annotated objects in QuPath will be exported using an available script provided in StarDist GitHub page to create masks for StarDist training , users can right click on the link and save the script.
To use it, just drag the saved script to QuPath and click on run for project as instructed below.

## Slide 18
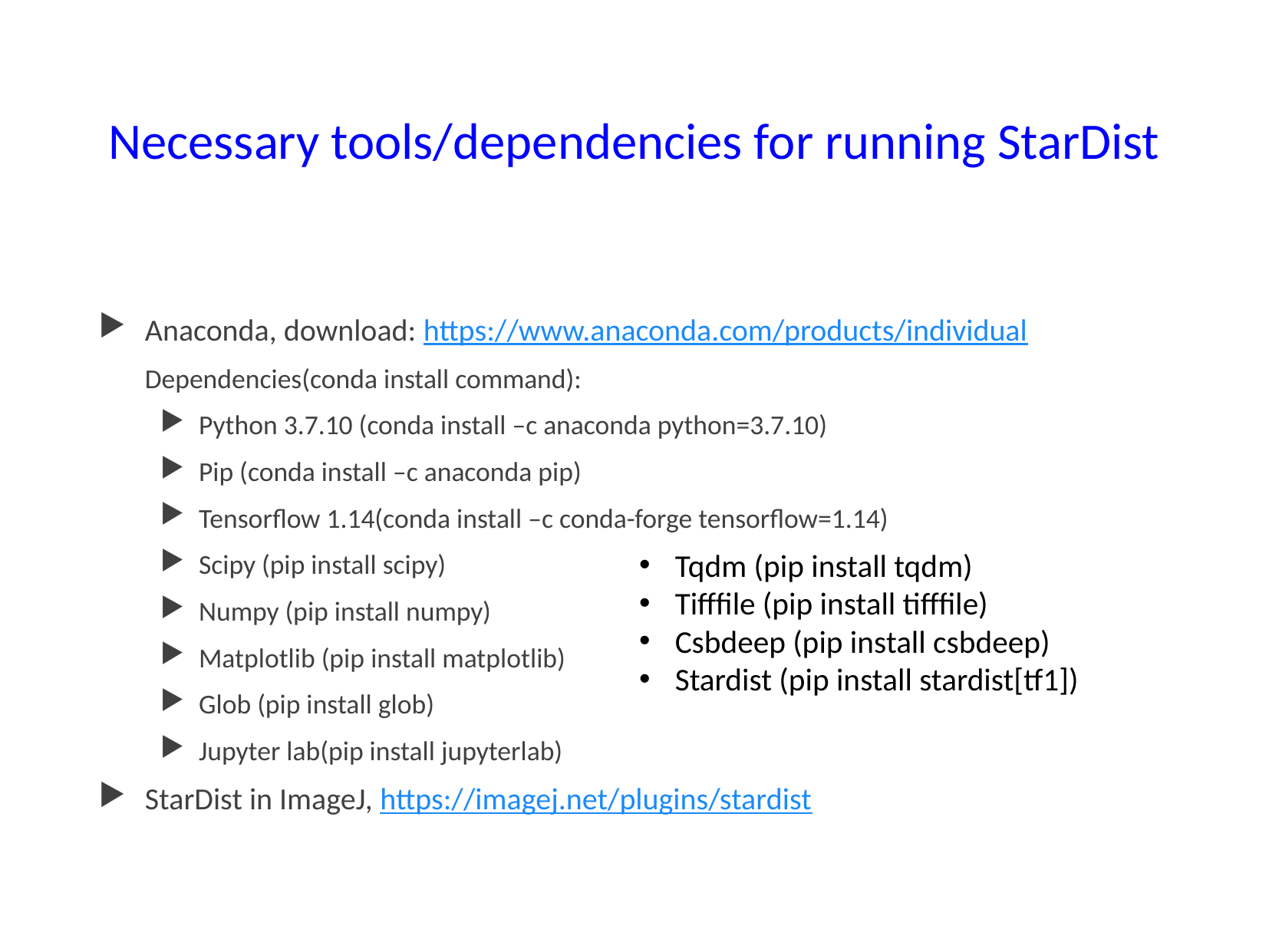

# Necessary tools/dependencies for running StarDist
Anaconda, download: https://www.anaconda.com/products/individual
Dependencies(conda install command):
Python 3.7.10 (conda install –c anaconda python=3.7.10)
Pip (conda install –c anaconda pip)
Tensorflow 1.14(conda install –c conda-forge tensorflow=1.14)
Scipy (pip install scipy)
Numpy (pip install numpy)
Matplotlib (pip install matplotlib)
Glob (pip install glob)
Jupyter lab(pip install jupyterlab)
StarDist in ImageJ, https://imagej.net/plugins/stardist
Tqdm (pip install tqdm)
Tifffile (pip install tifffile)
Csbdeep (pip install csbdeep)
Stardist (pip install stardist[tf1])

## Slide 19
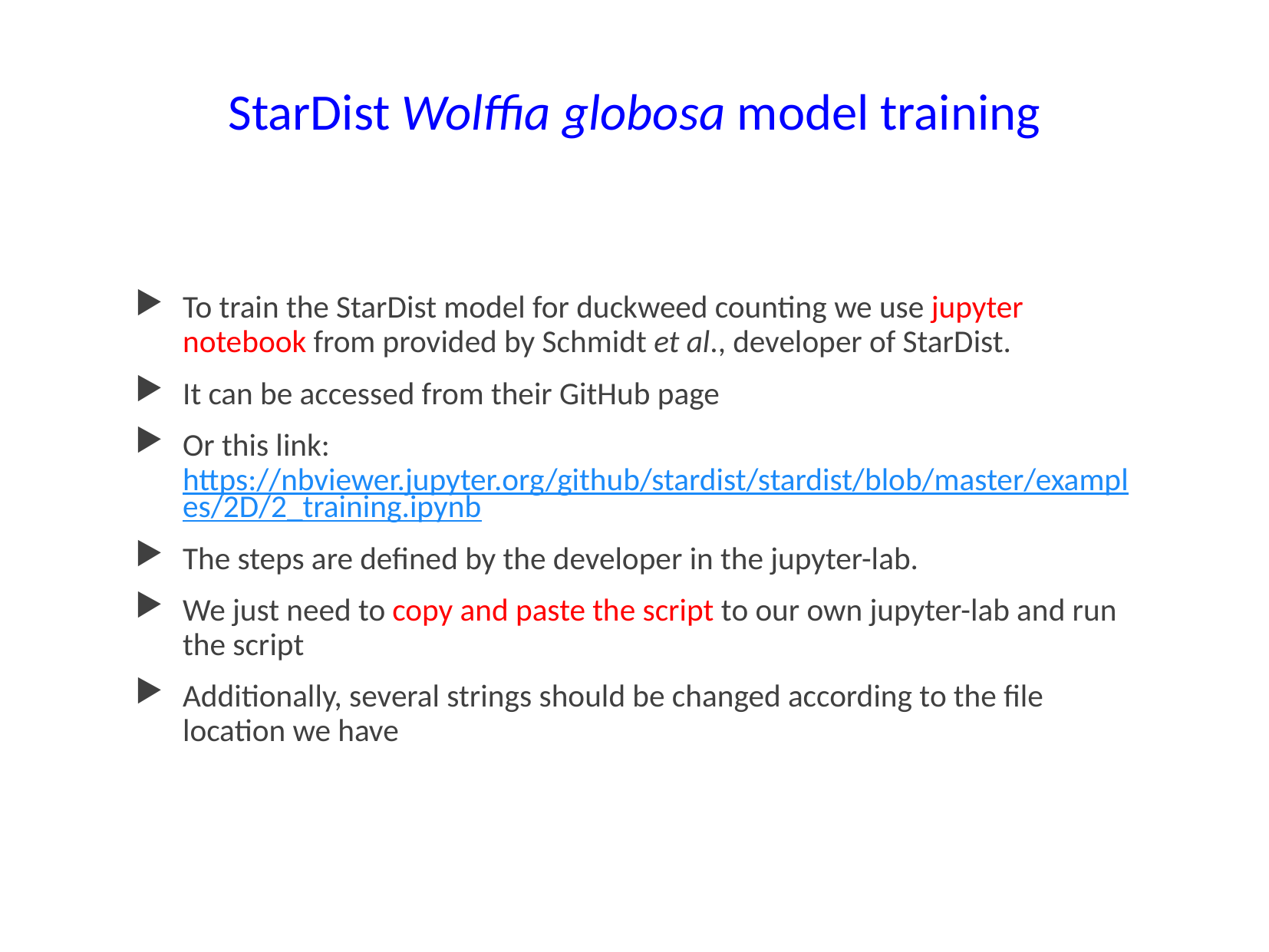

# StarDist Wolffia globosa model training
To train the StarDist model for duckweed counting we use jupyter notebook from provided by Schmidt et al., developer of StarDist.
It can be accessed from their GitHub page
Or this link: https://nbviewer.jupyter.org/github/stardist/stardist/blob/master/examples/2D/2_training.ipynb
The steps are defined by the developer in the jupyter-lab.
We just need to copy and paste the script to our own jupyter-lab and run the script
Additionally, several strings should be changed according to the file location we have

## Slide 20
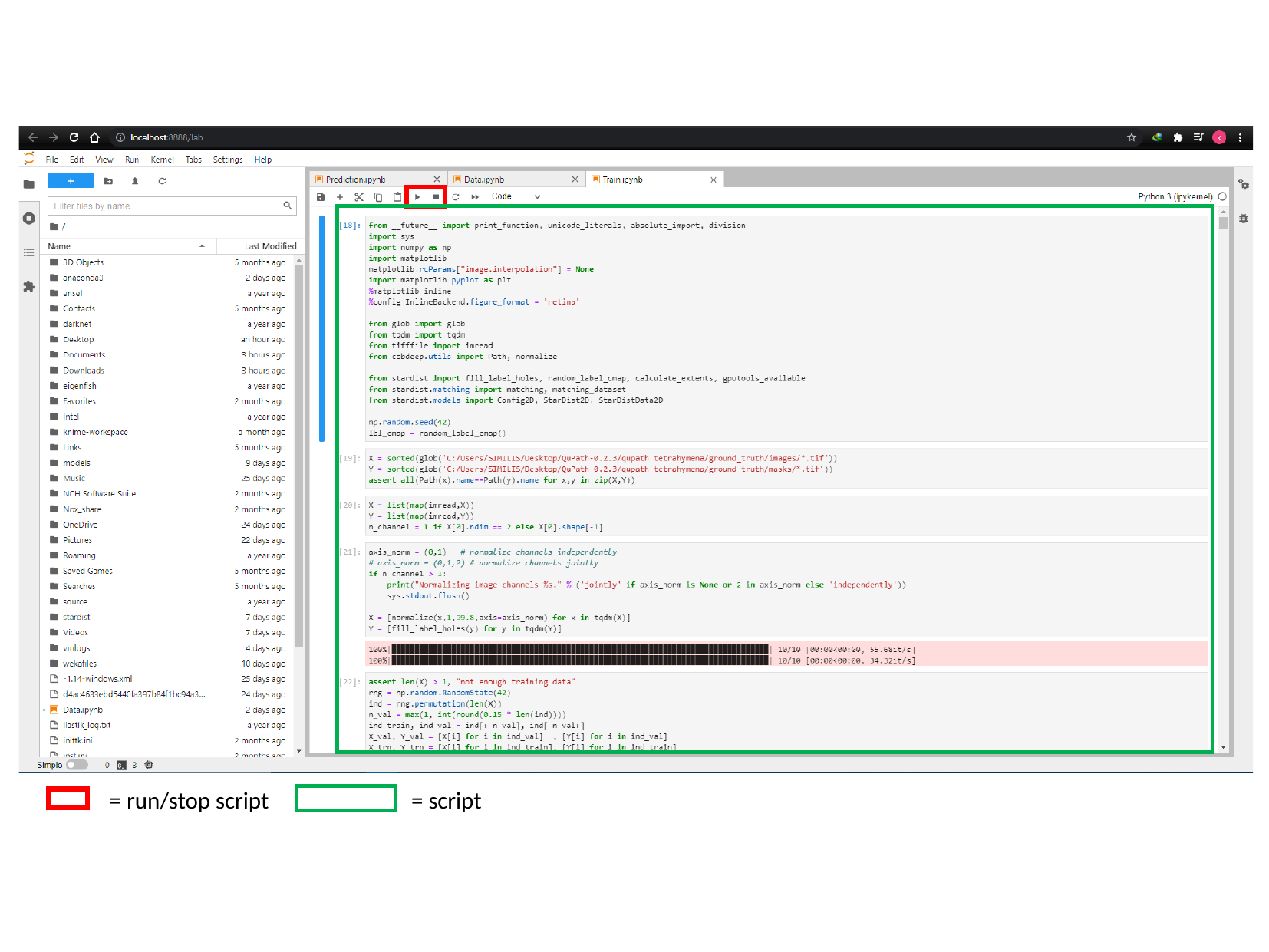

= run/stop script
= script

## Slide 21
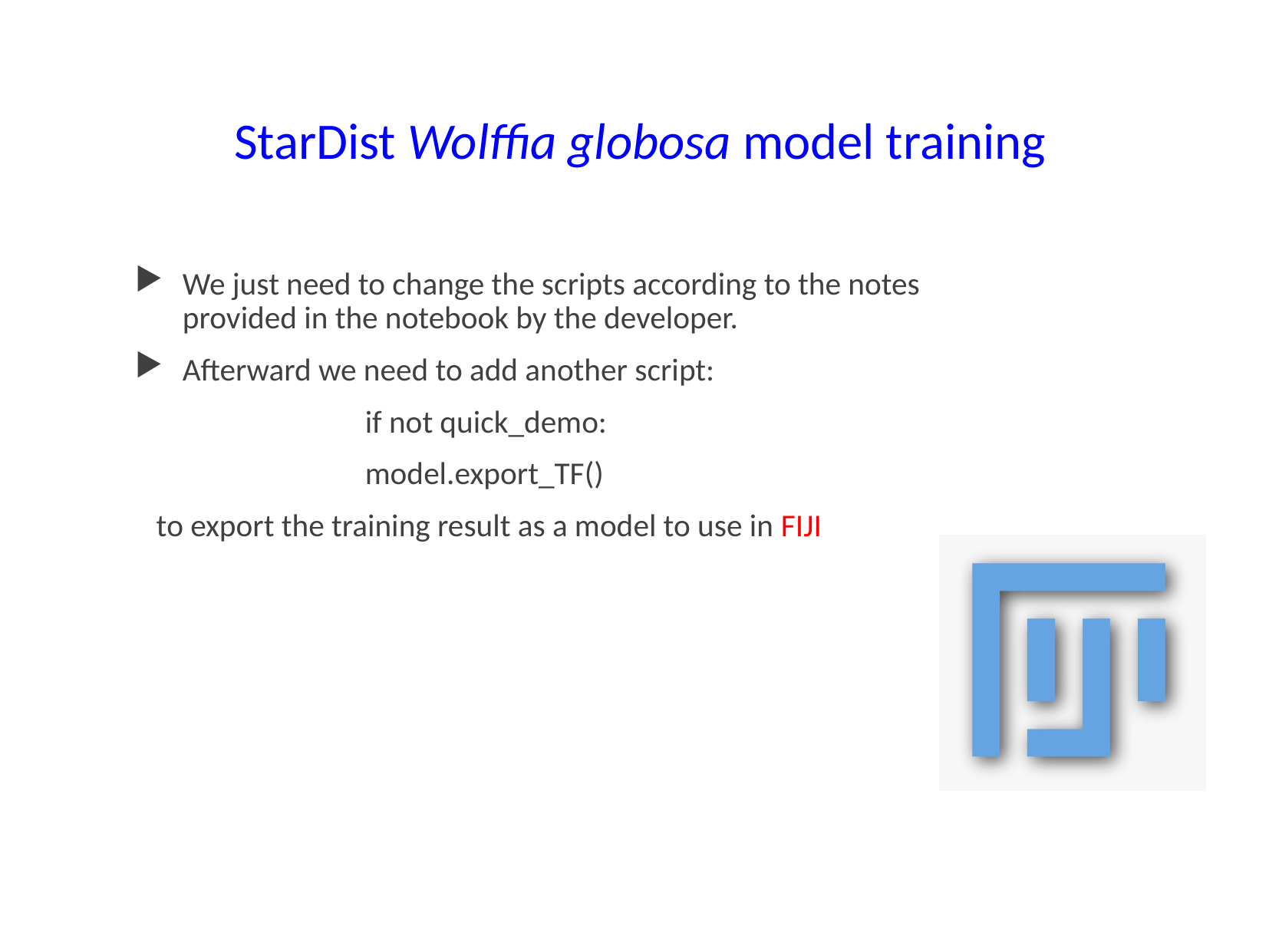

# StarDist Wolffia globosa model training
We just need to change the scripts according to the notes provided in the notebook by the developer.
Afterward we need to add another script:
		if not quick_demo:
 		model.export_TF()
 to export the training result as a model to use in FIJI

## Slide 22
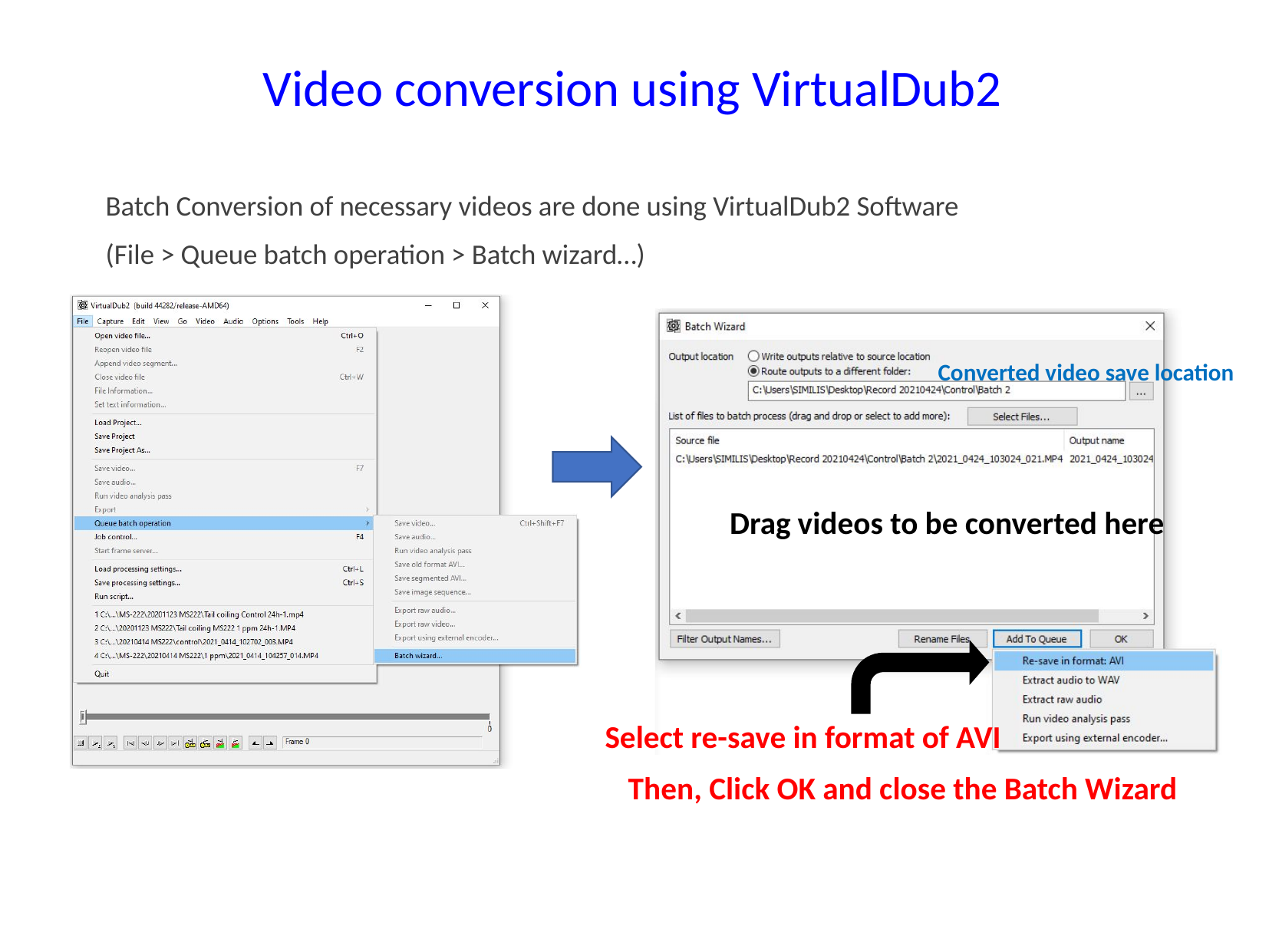

# Video conversion using VirtualDub2
Batch Conversion of necessary videos are done using VirtualDub2 Software
(File > Queue batch operation > Batch wizard…)
Converted video save location
Drag videos to be converted here
Select re-save in format of AVI
Then, Click OK and close the Batch Wizard

## Slide 23
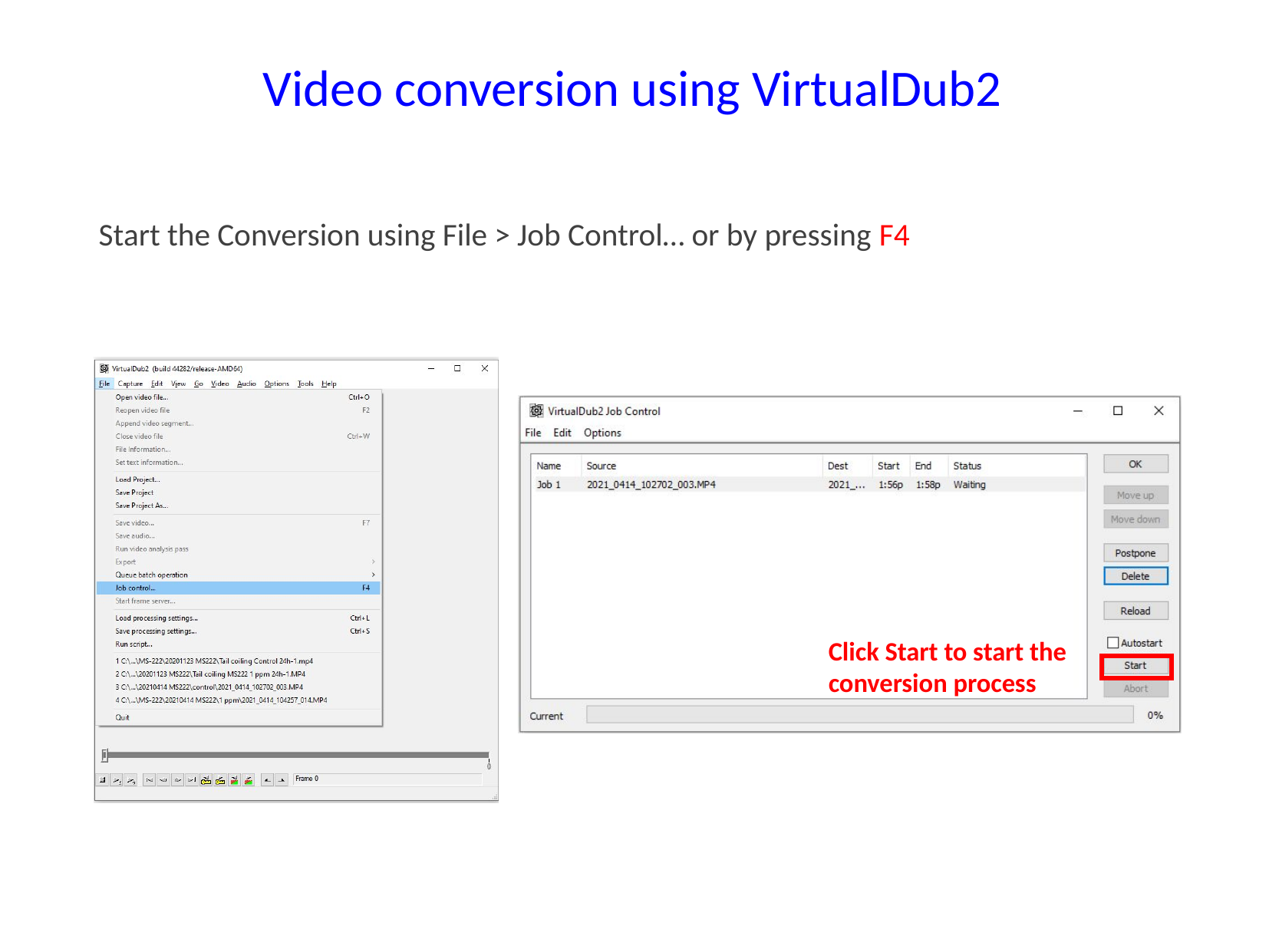

Video conversion using VirtualDub2
Start the Conversion using File > Job Control… or by pressing F4
Click Start to start the conversion process

## Slide 24
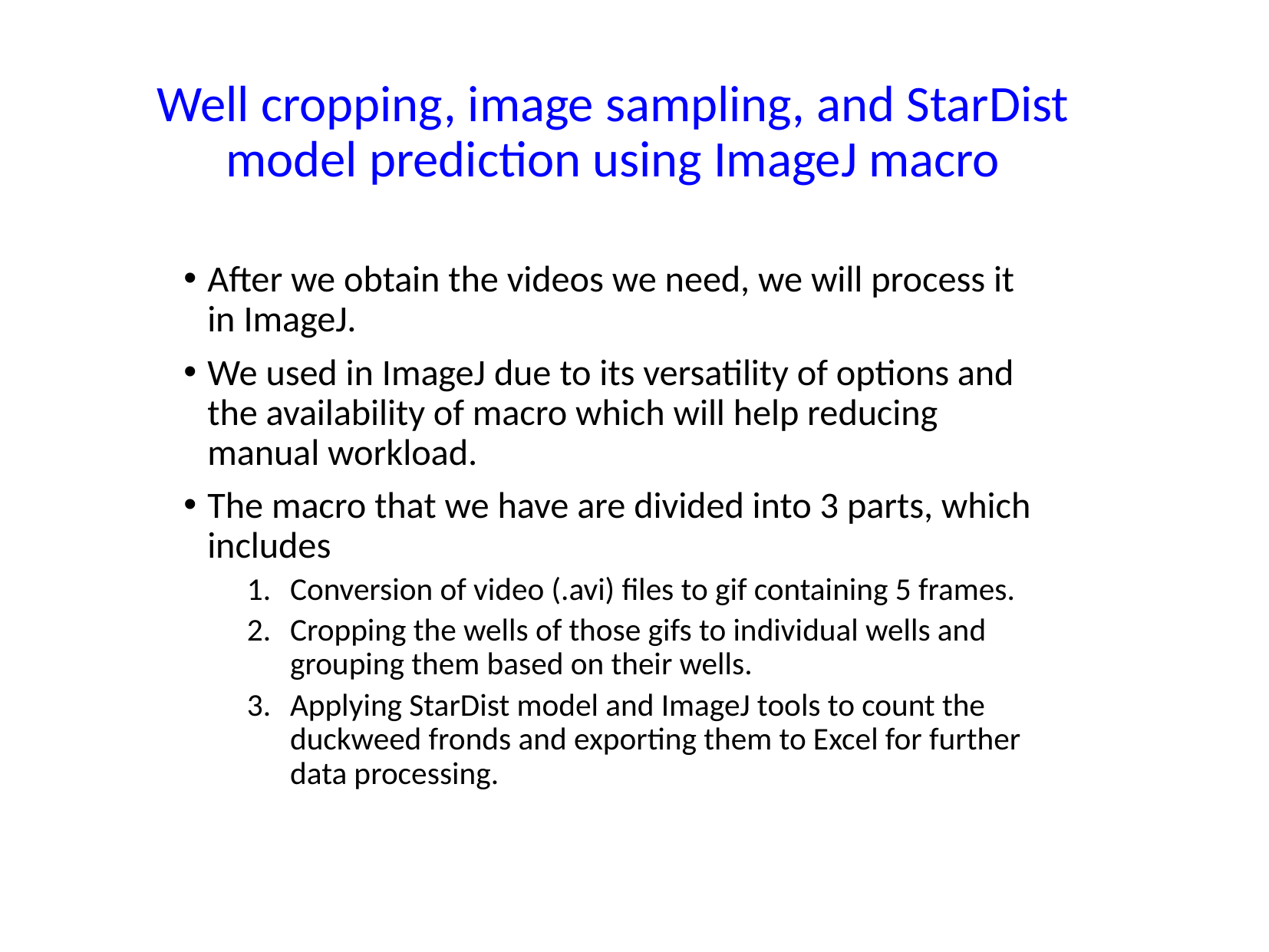

# Well cropping, image sampling, and StarDist model prediction using ImageJ macro
After we obtain the videos we need, we will process it in ImageJ.
We used in ImageJ due to its versatility of options and the availability of macro which will help reducing manual workload.
The macro that we have are divided into 3 parts, which includes
Conversion of video (.avi) files to gif containing 5 frames.
Cropping the wells of those gifs to individual wells and grouping them based on their wells.
Applying StarDist model and ImageJ tools to count the duckweed fronds and exporting them to Excel for further data processing.

## Slide 25
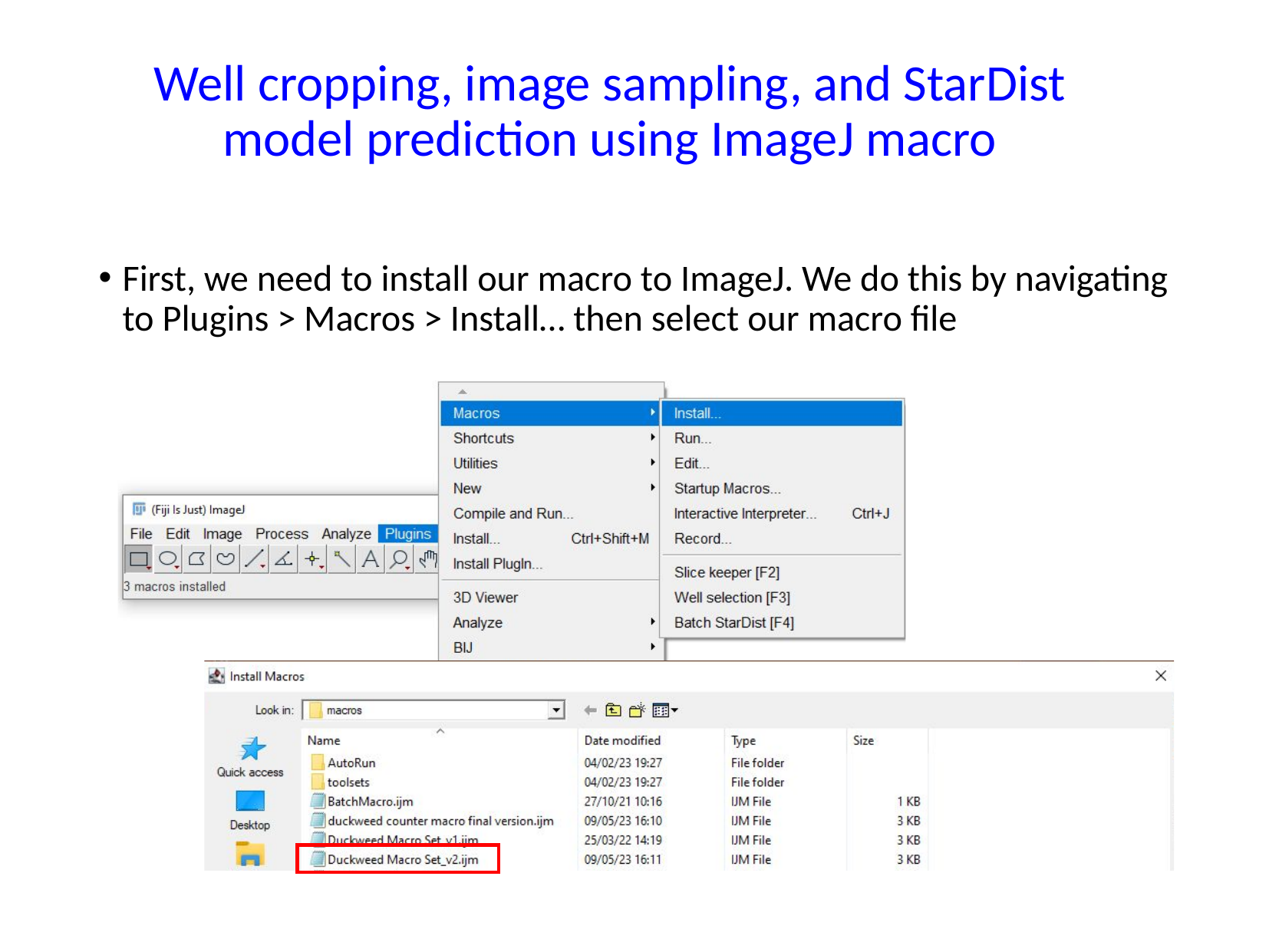

# Well cropping, image sampling, and StarDist model prediction using ImageJ macro
First, we need to install our macro to ImageJ. We do this by navigating to Plugins > Macros > Install… then select our macro file

## Slide 26
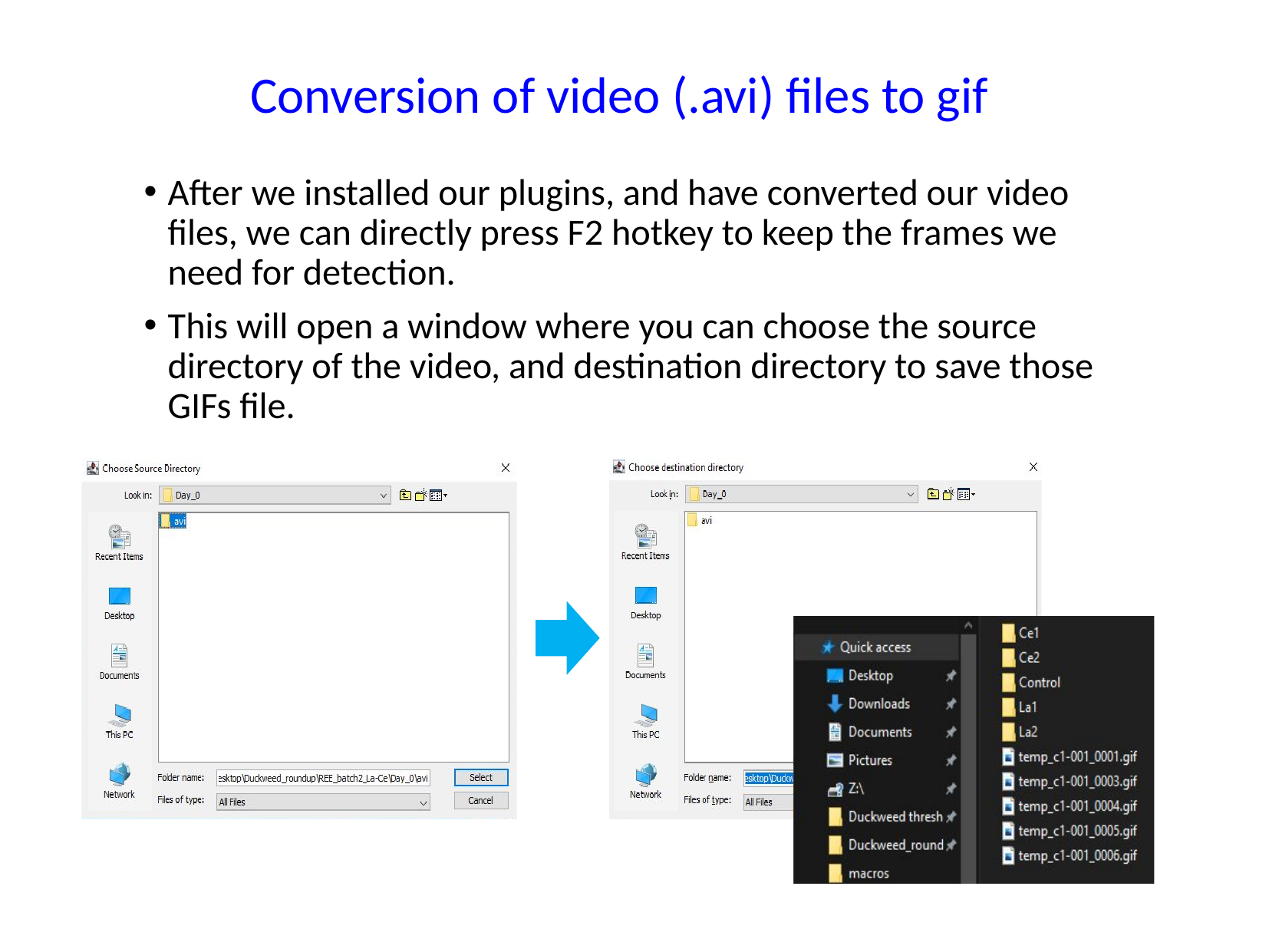

# Conversion of video (.avi) files to gif
After we installed our plugins, and have converted our video files, we can directly press F2 hotkey to keep the frames we need for detection.
This will open a window where you can choose the source directory of the video, and destination directory to save those GIFs file.

## Slide 27
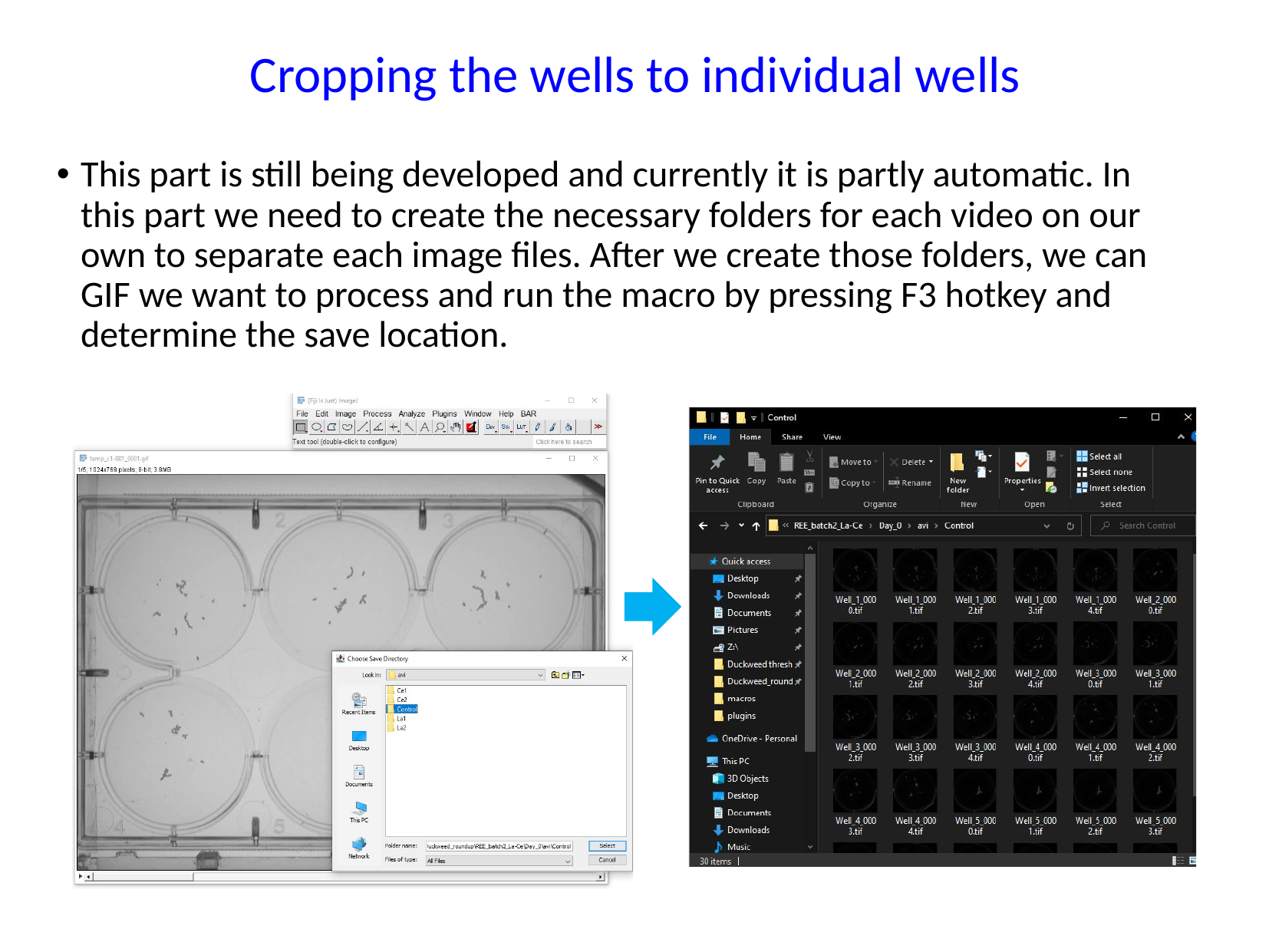

# Cropping the wells to individual wells
This part is still being developed and currently it is partly automatic. In this part we need to create the necessary folders for each video on our own to separate each image files. After we create those folders, we can GIF we want to process and run the macro by pressing F3 hotkey and determine the save location.

## Slide 28
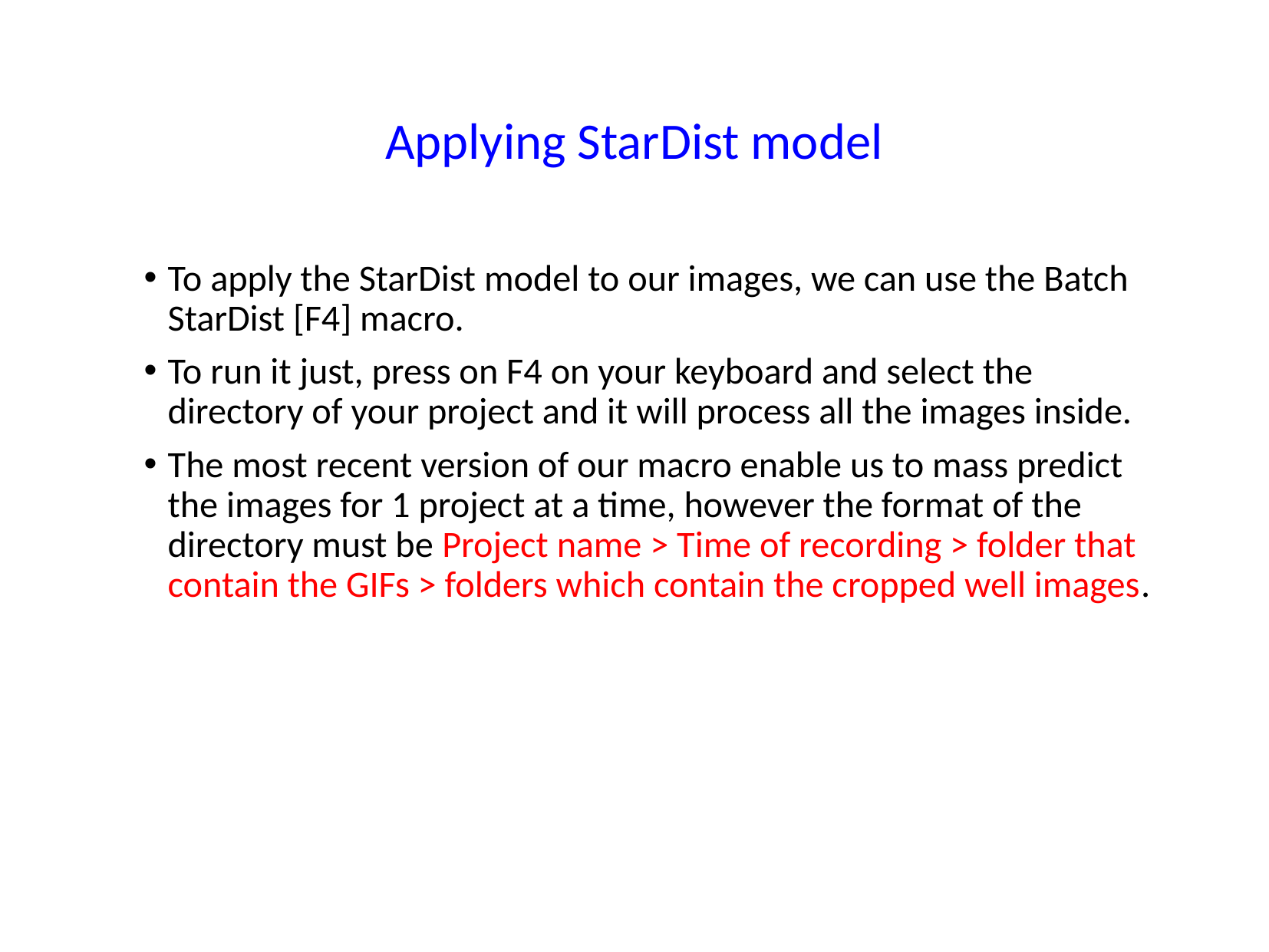

# Applying StarDist model
To apply the StarDist model to our images, we can use the Batch StarDist [F4] macro.
To run it just, press on F4 on your keyboard and select the directory of your project and it will process all the images inside.
The most recent version of our macro enable us to mass predict the images for 1 project at a time, however the format of the directory must be Project name > Time of recording > folder that contain the GIFs > folders which contain the cropped well images.

## Slide 29
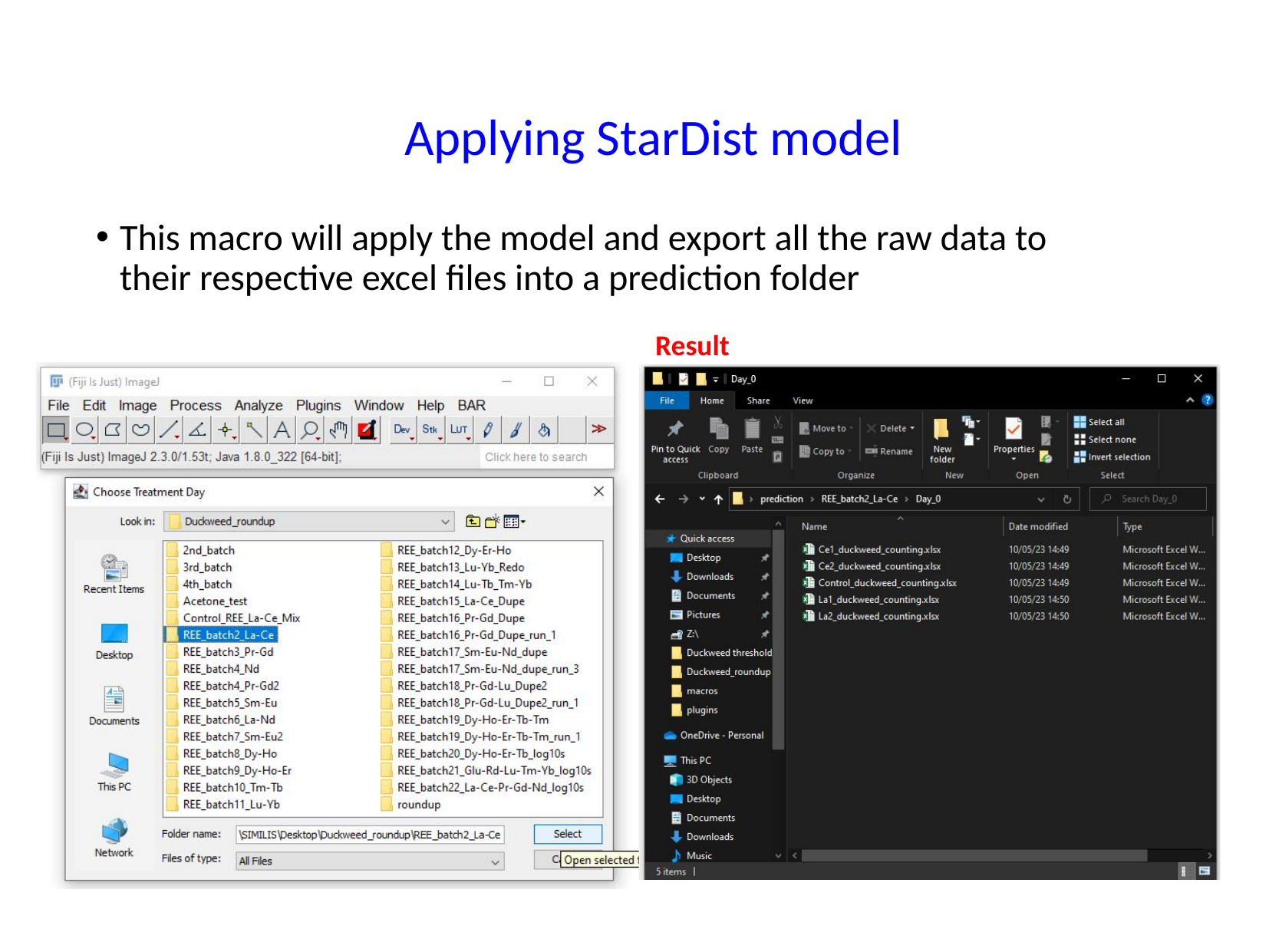

Applying StarDist model
This macro will apply the model and export all the raw data to their respective excel files into a prediction folder
Result

## Slide 30
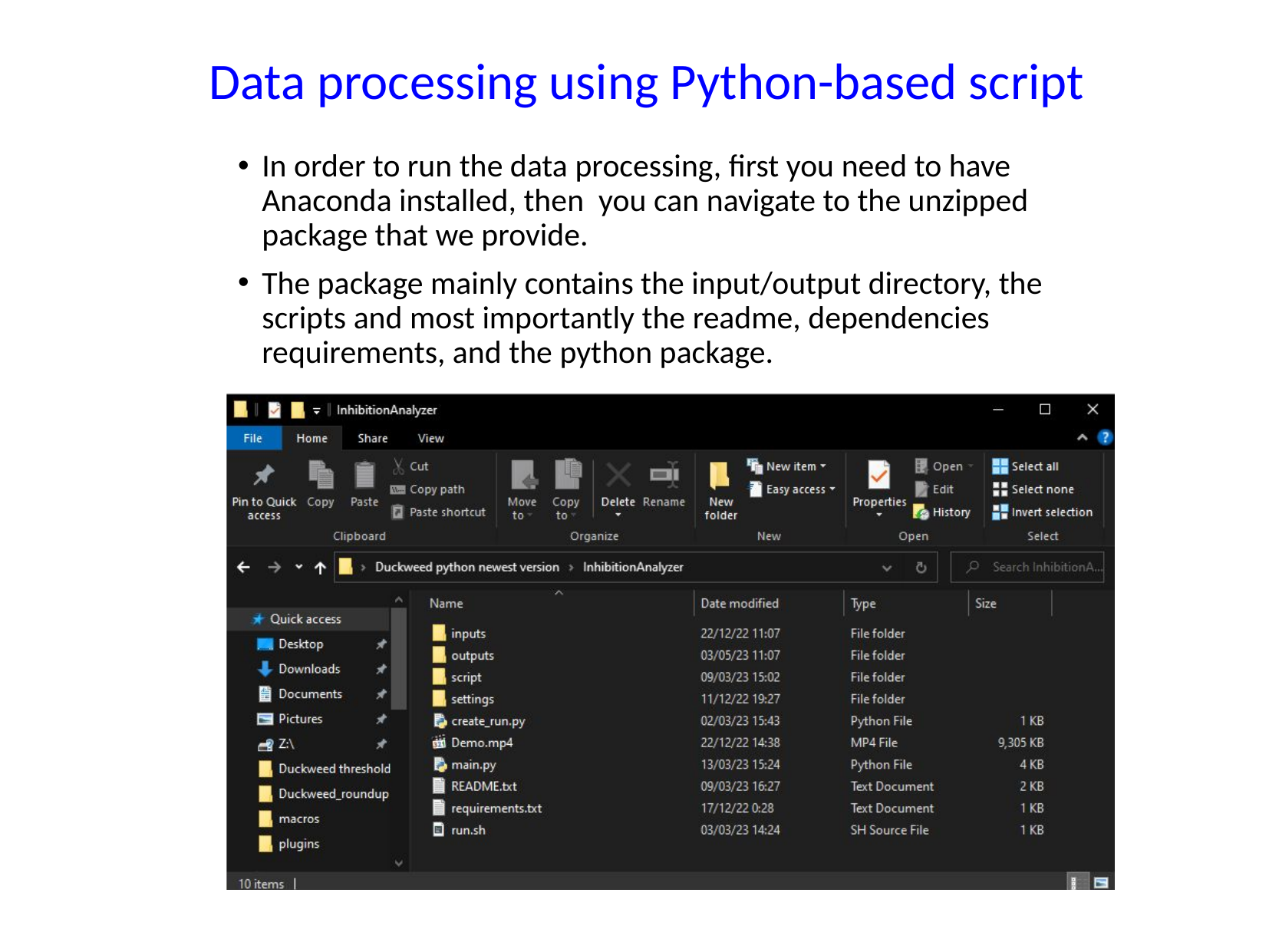

# Data processing using Python-based script
In order to run the data processing, first you need to have Anaconda installed, then you can navigate to the unzipped package that we provide.
The package mainly contains the input/output directory, the scripts and most importantly the readme, dependencies requirements, and the python package.

## Slide 31
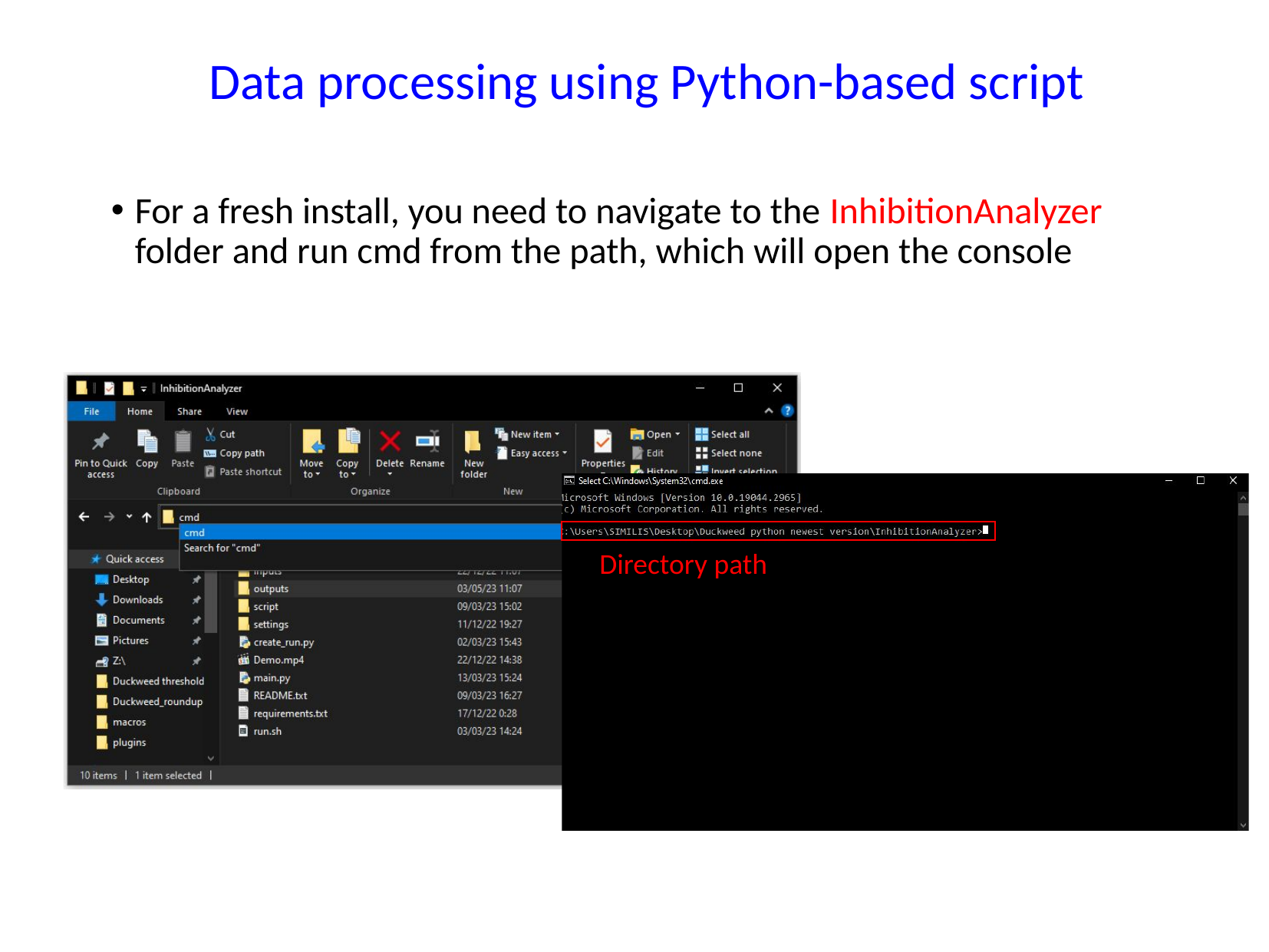

# Data processing using Python-based script
For a fresh install, you need to navigate to the InhibitionAnalyzer folder and run cmd from the path, which will open the console
Directory path

## Slide 32
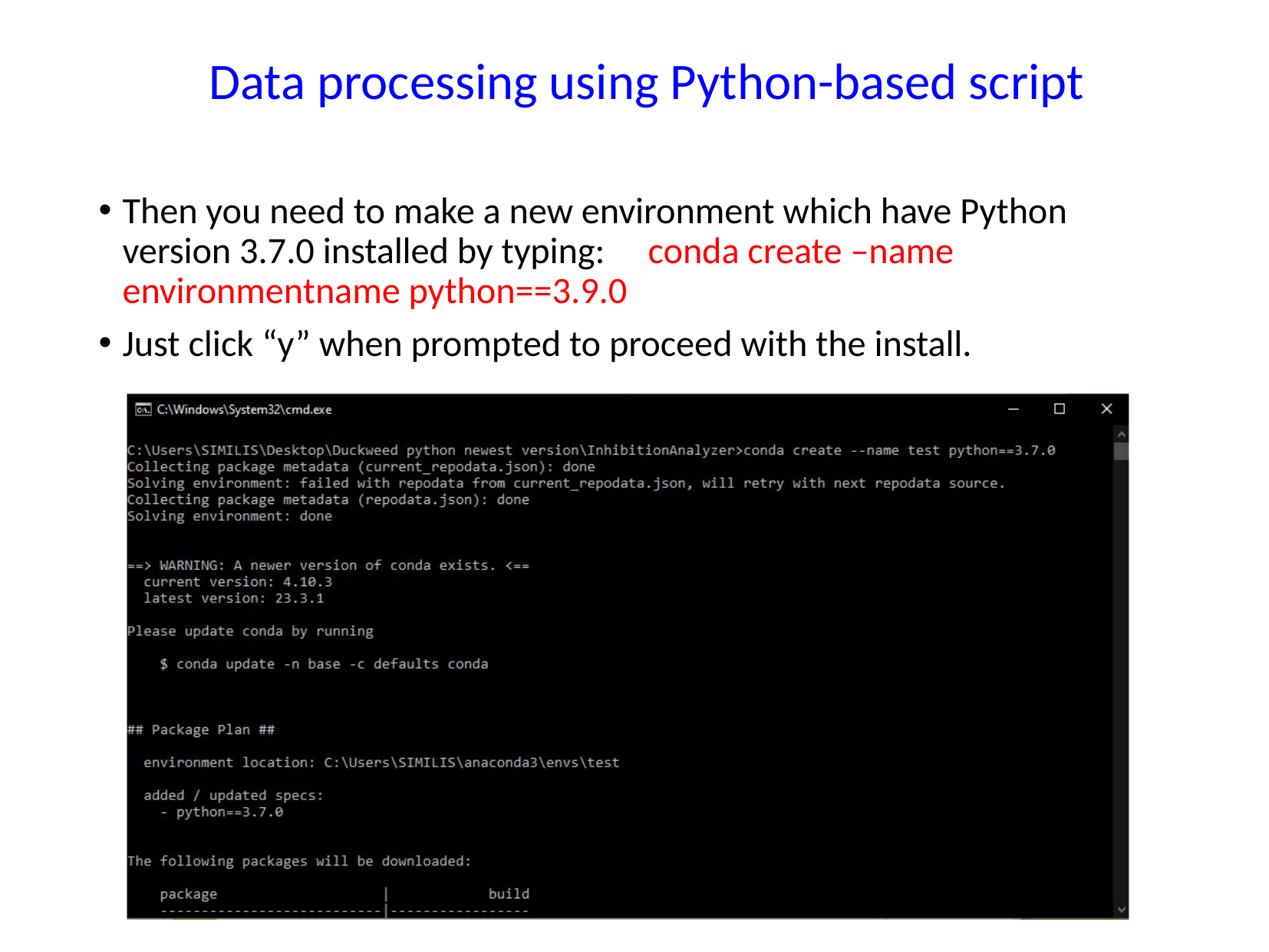

# Data processing using Python-based script
Then you need to make a new environment which have Python version 3.7.0 installed by typing: conda create –name environmentname python==3.9.0
Just click “y” when prompted to proceed with the install.

## Slide 33
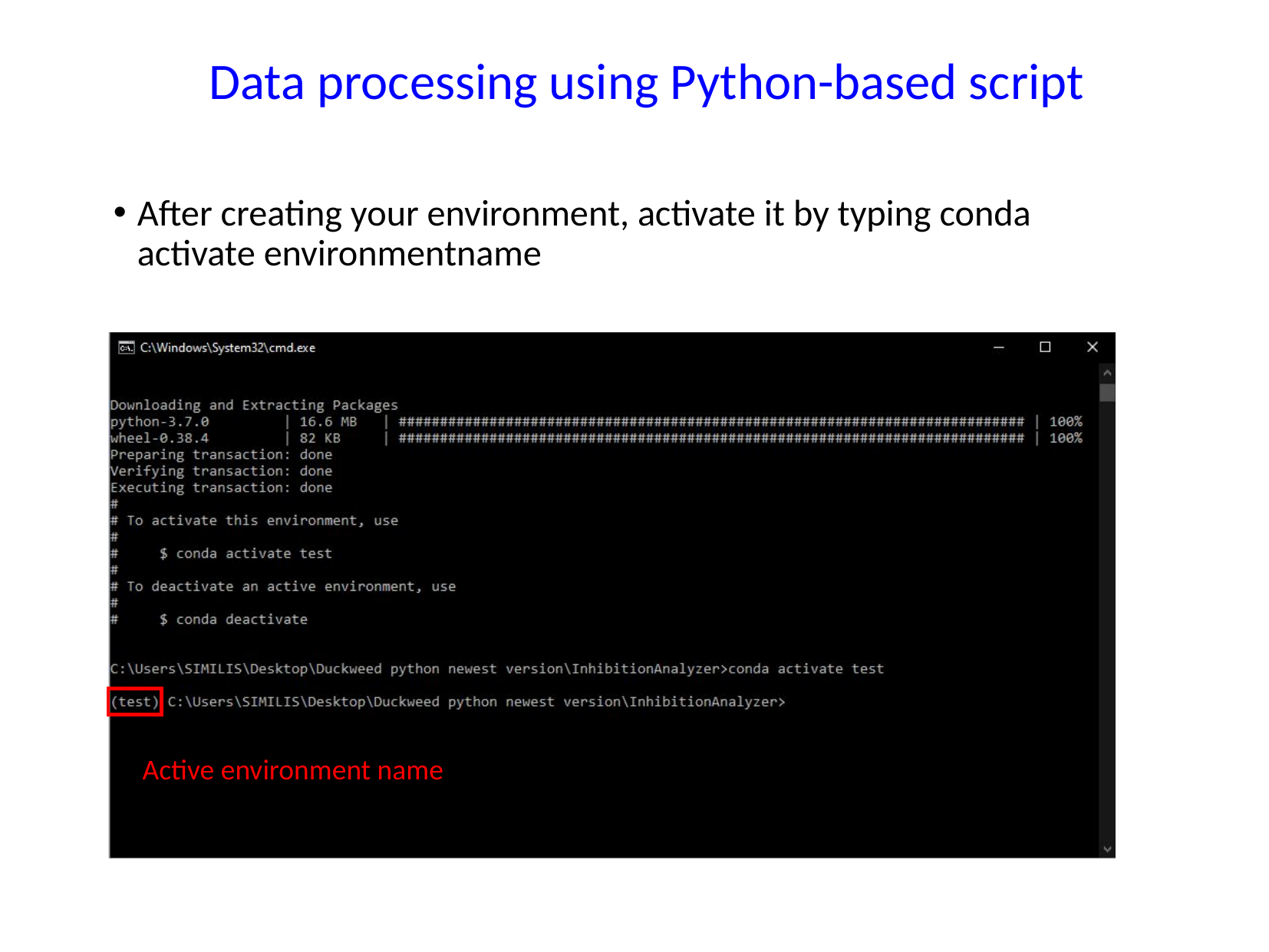

# Data processing using Python-based script
After creating your environment, activate it by typing conda activate environmentname
Active environment name

## Slide 34
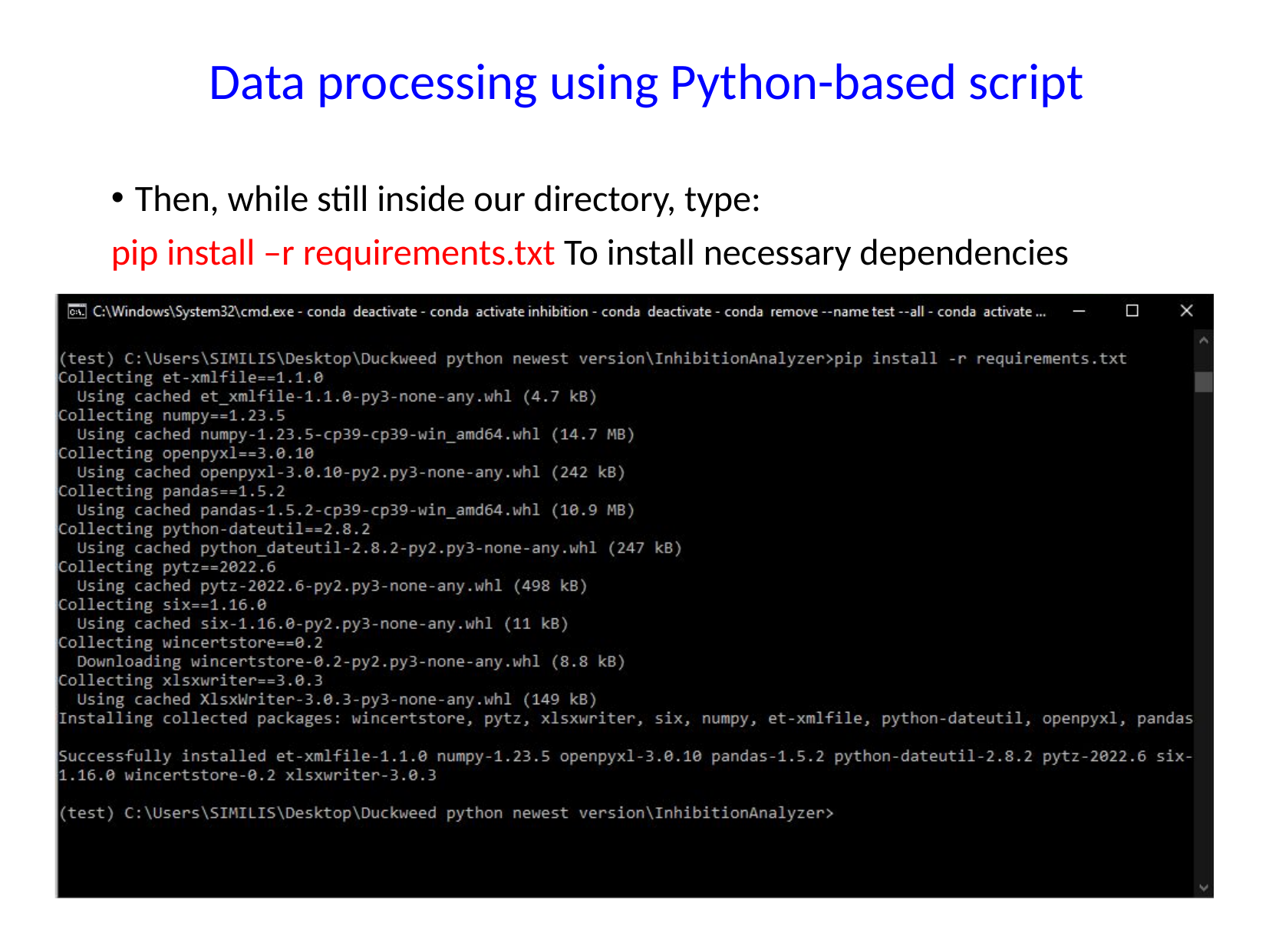

Data processing using Python-based script
Then, while still inside our directory, type:
pip install –r requirements.txt To install necessary dependencies

## Slide 35
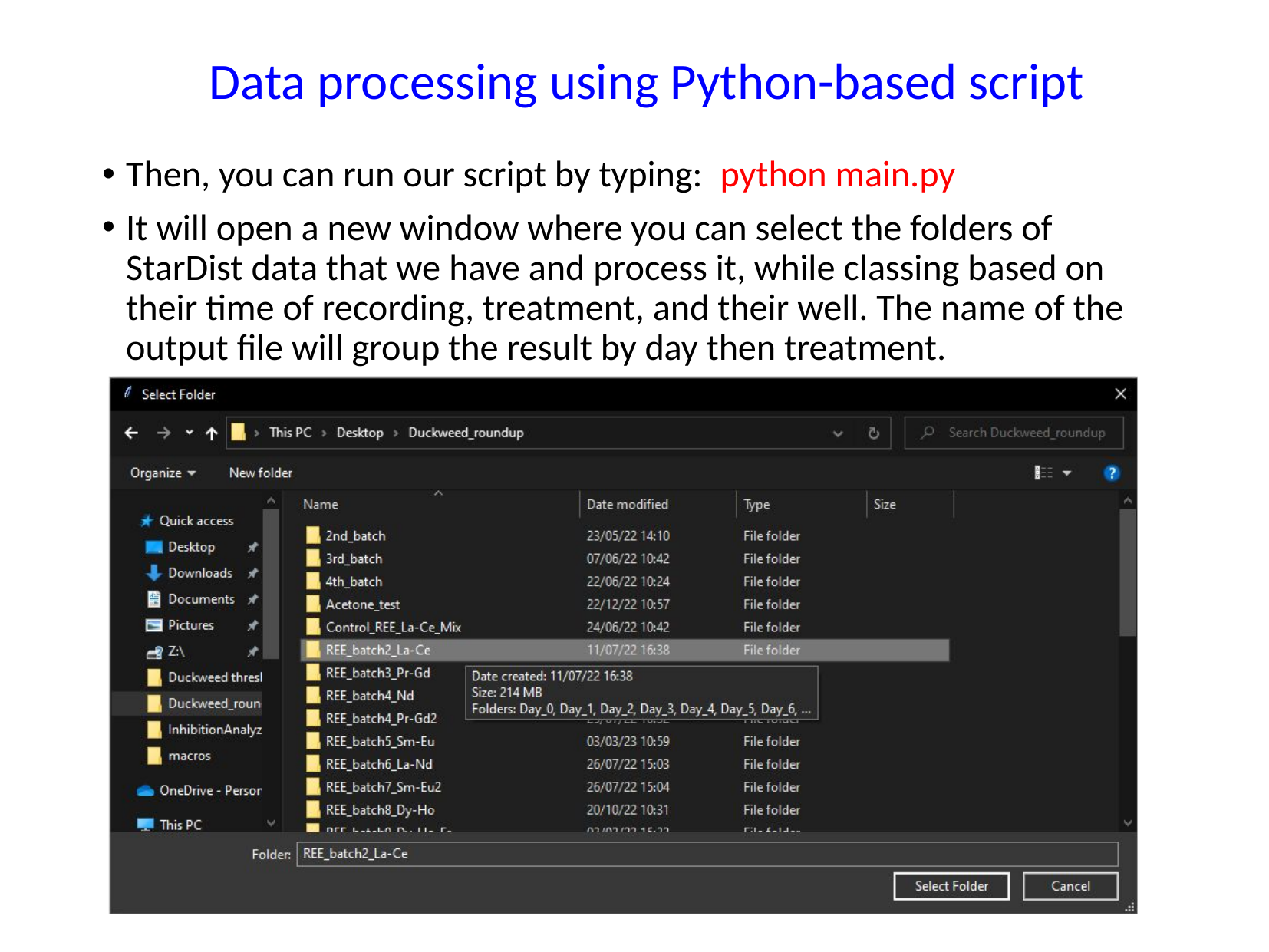

Data processing using Python-based script
Then, you can run our script by typing: python main.py
It will open a new window where you can select the folders of StarDist data that we have and process it, while classing based on their time of recording, treatment, and their well. The name of the output file will group the result by day then treatment.

## Slide 36
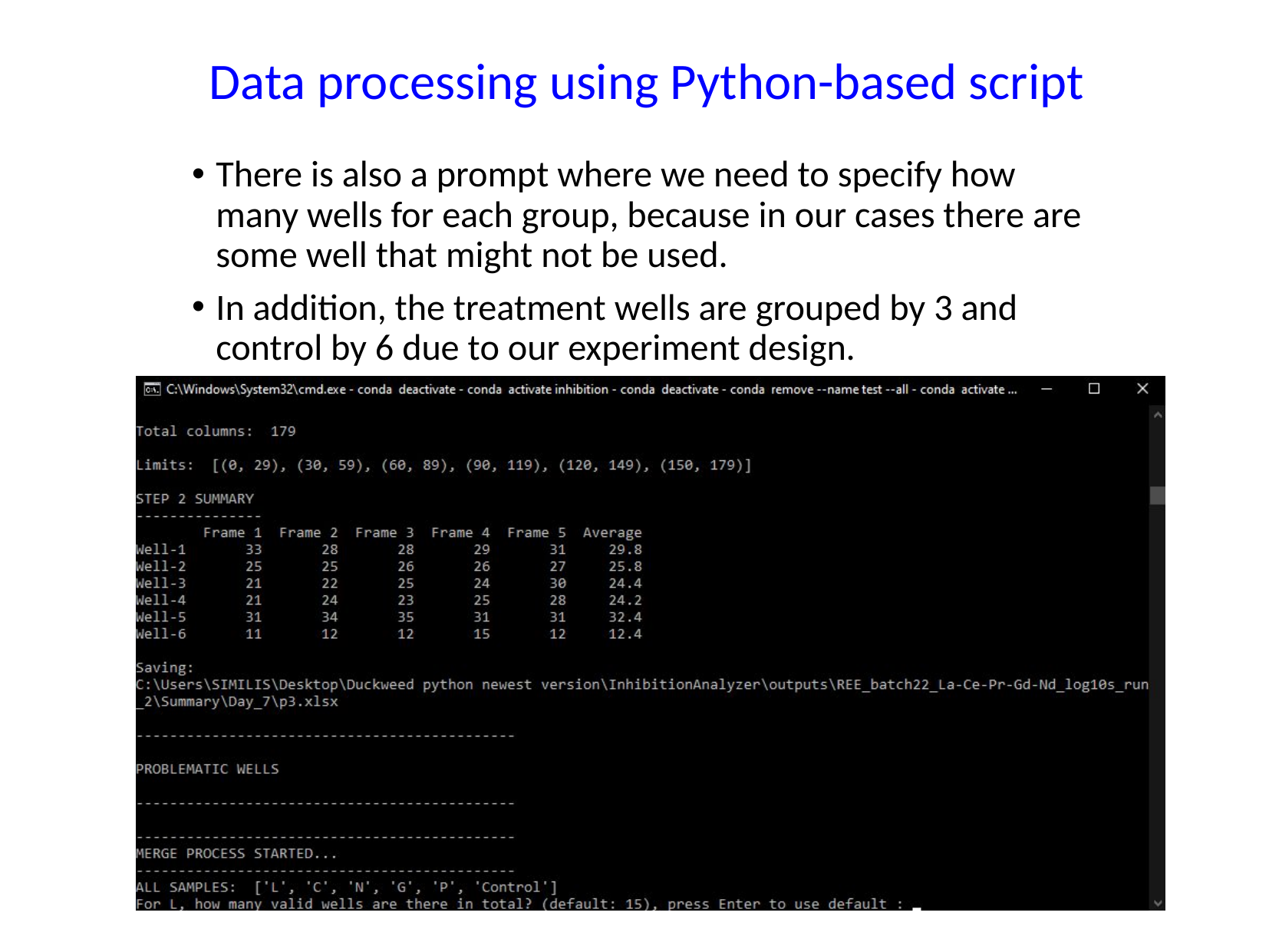

Data processing using Python-based script
There is also a prompt where we need to specify how many wells for each group, because in our cases there are some well that might not be used.
In addition, the treatment wells are grouped by 3 and control by 6 due to our experiment design.

## Slide 37
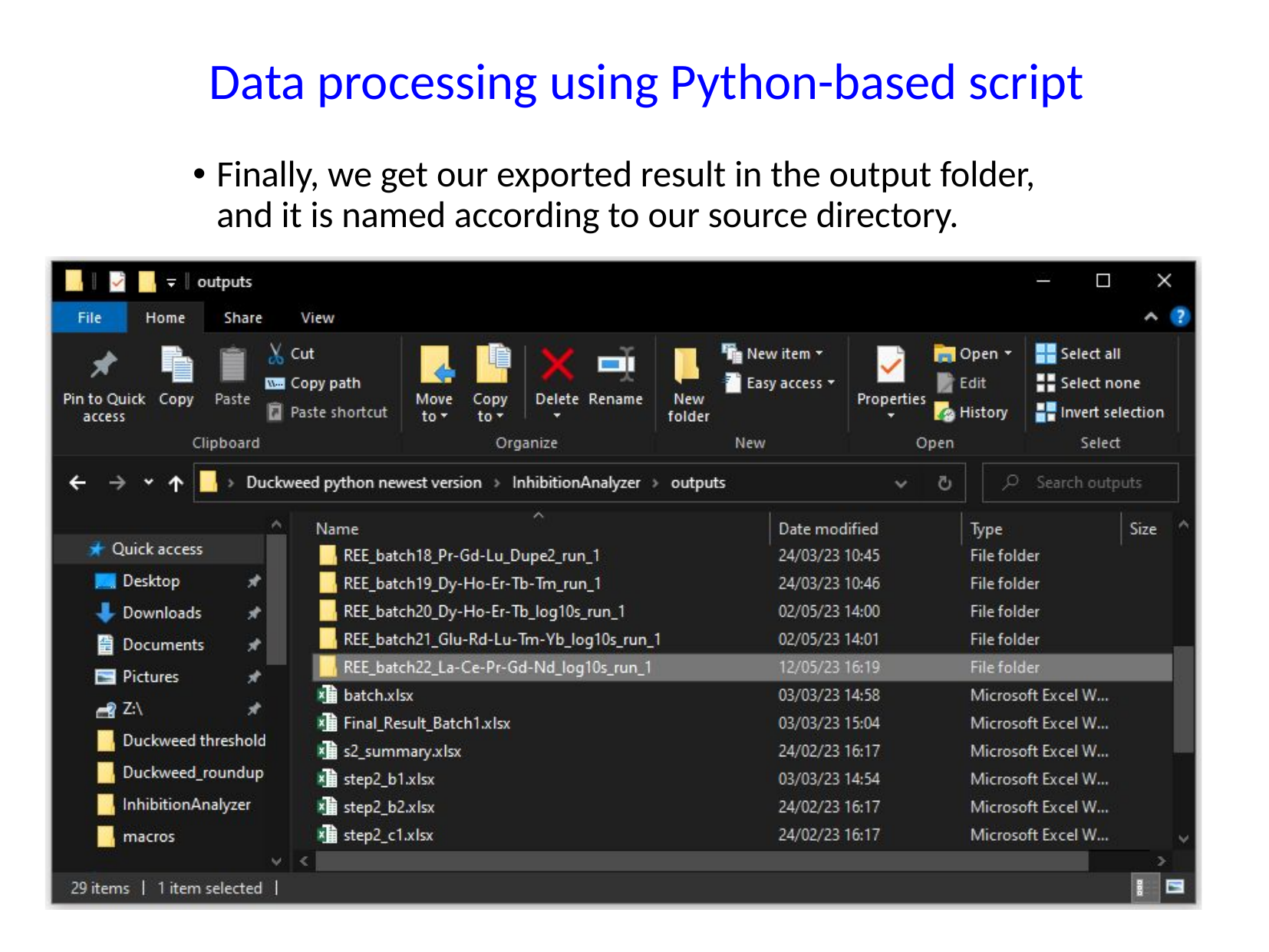

Data processing using Python-based script
Finally, we get our exported result in the output folder, and it is named according to our source directory.

## Slide 38
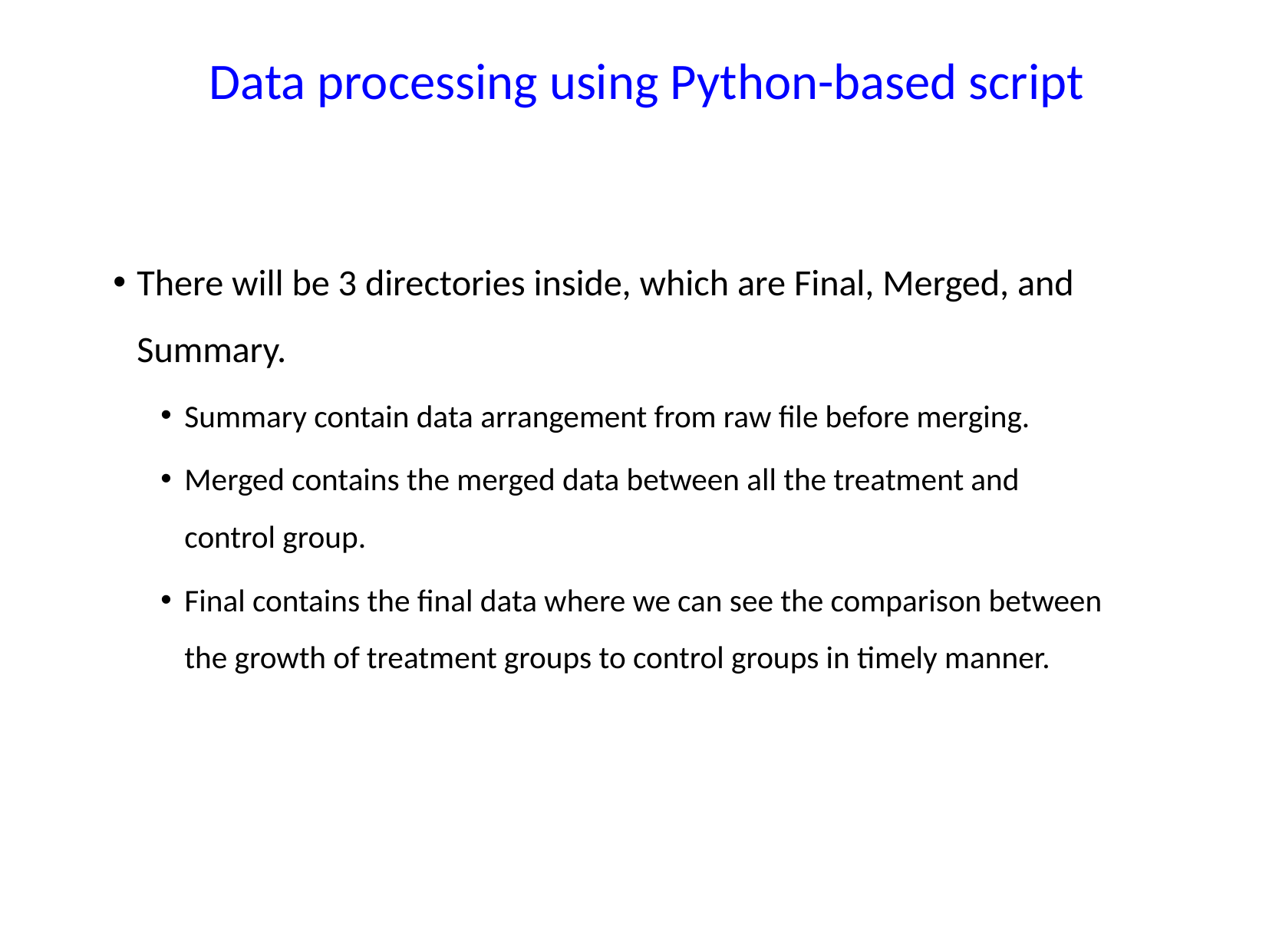

Data processing using Python-based script
There will be 3 directories inside, which are Final, Merged, and Summary.
Summary contain data arrangement from raw file before merging.
Merged contains the merged data between all the treatment and control group.
Final contains the final data where we can see the comparison between the growth of treatment groups to control groups in timely manner.

## Slide 39
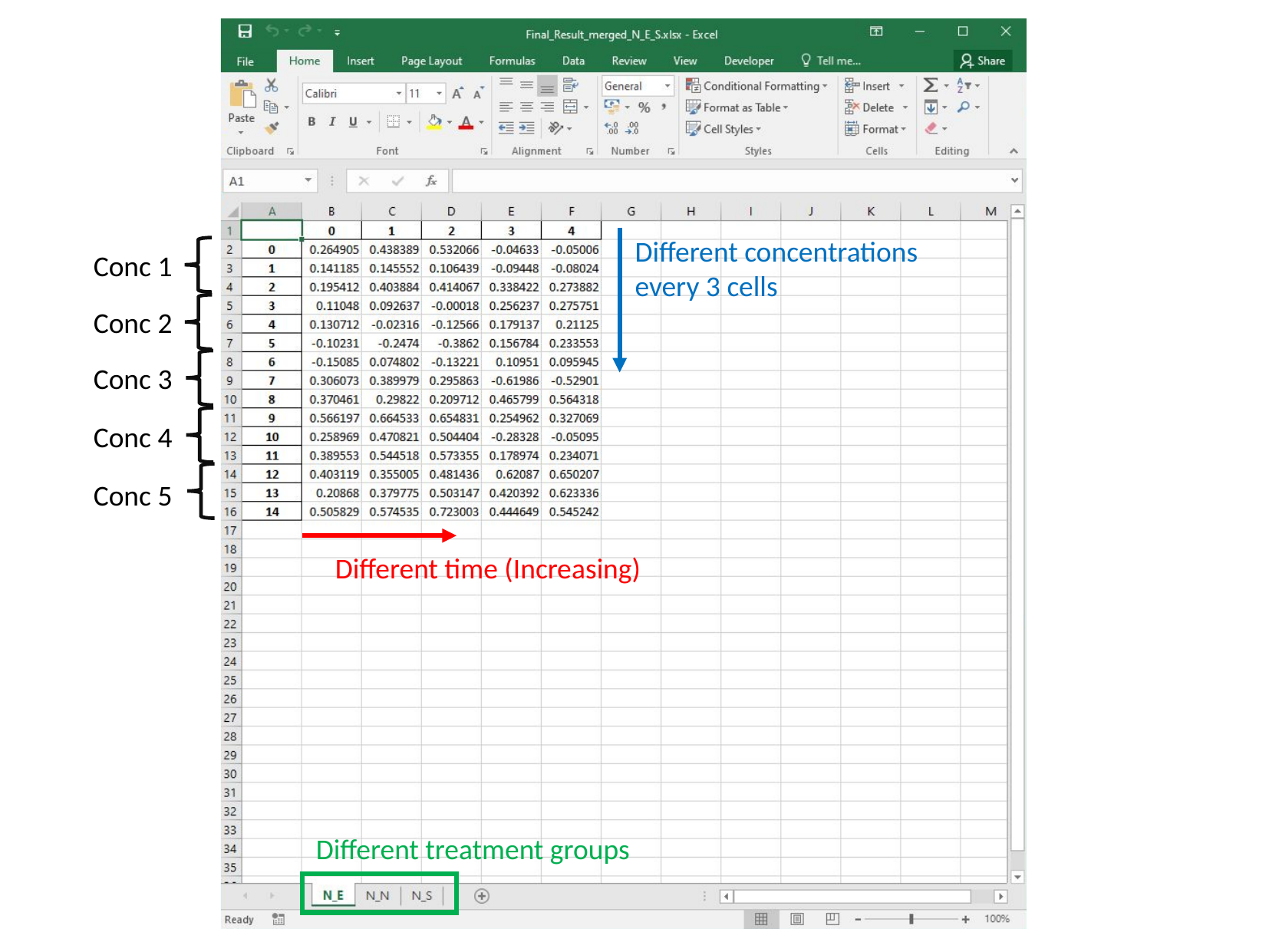

Different concentrations every 3 cells
Conc 1
Conc 2
Conc 3
Conc 4
Conc 5
Different time (Increasing)
Different treatment groups

## Slide 40
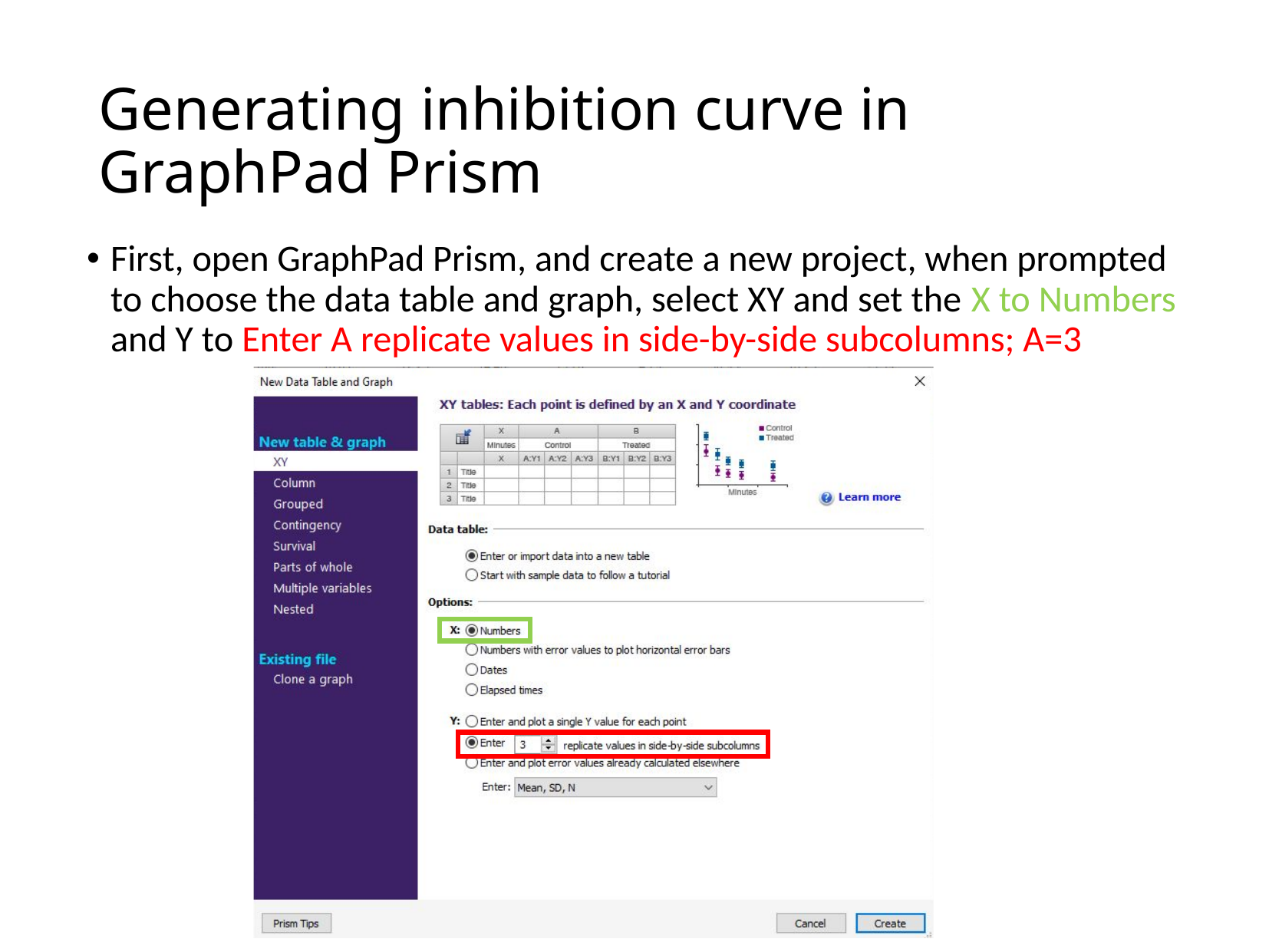

# Generating inhibition curve in GraphPad Prism
First, open GraphPad Prism, and create a new project, when prompted to choose the data table and graph, select XY and set the X to Numbers and Y to Enter A replicate values in side-by-side subcolumns; A=3

## Slide 41
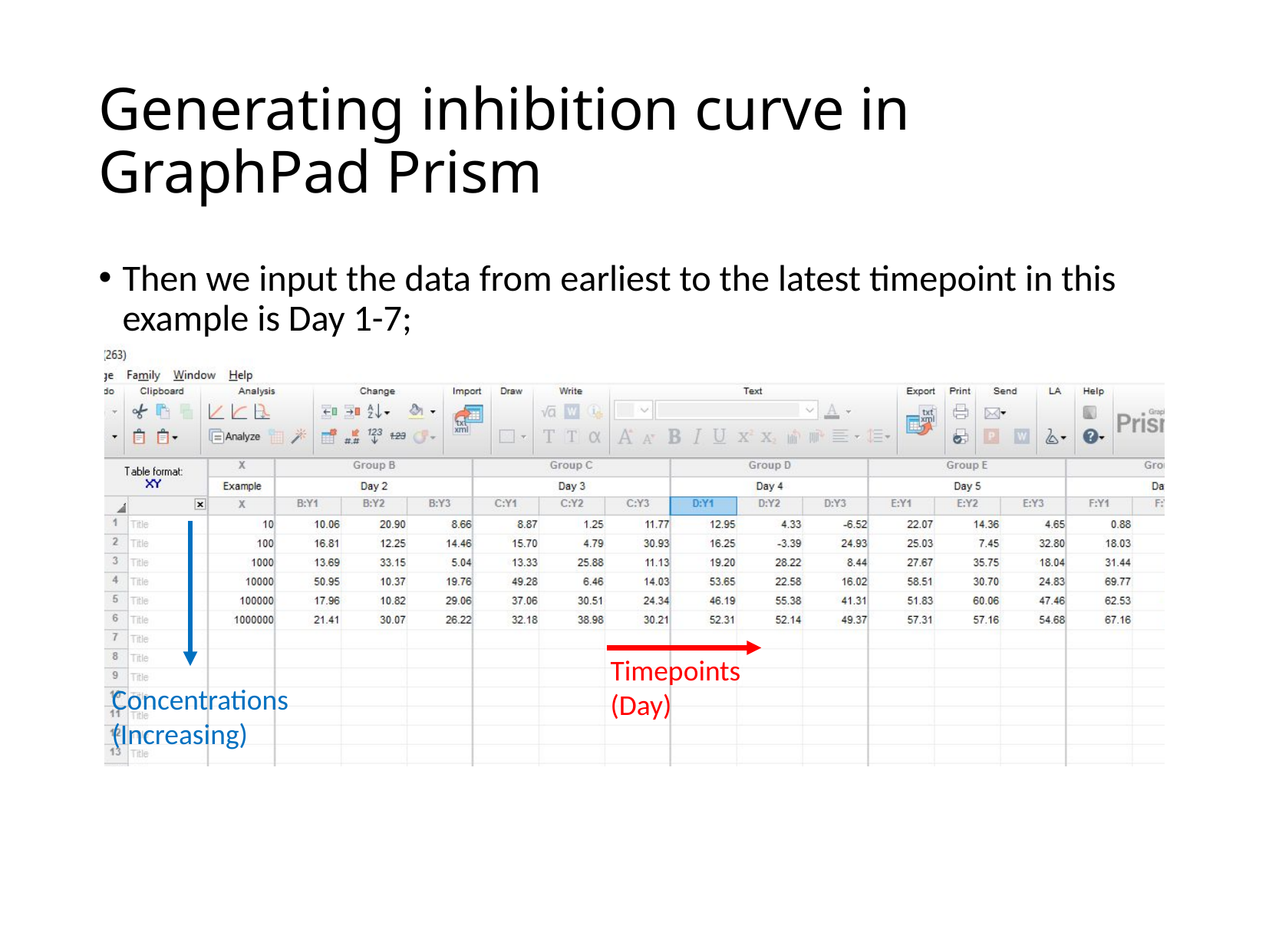

# Generating inhibition curve in GraphPad Prism
Then we input the data from earliest to the latest timepoint in this example is Day 1-7;
Timepoints
(Day)
Concentrations (Increasing)

## Slide 42
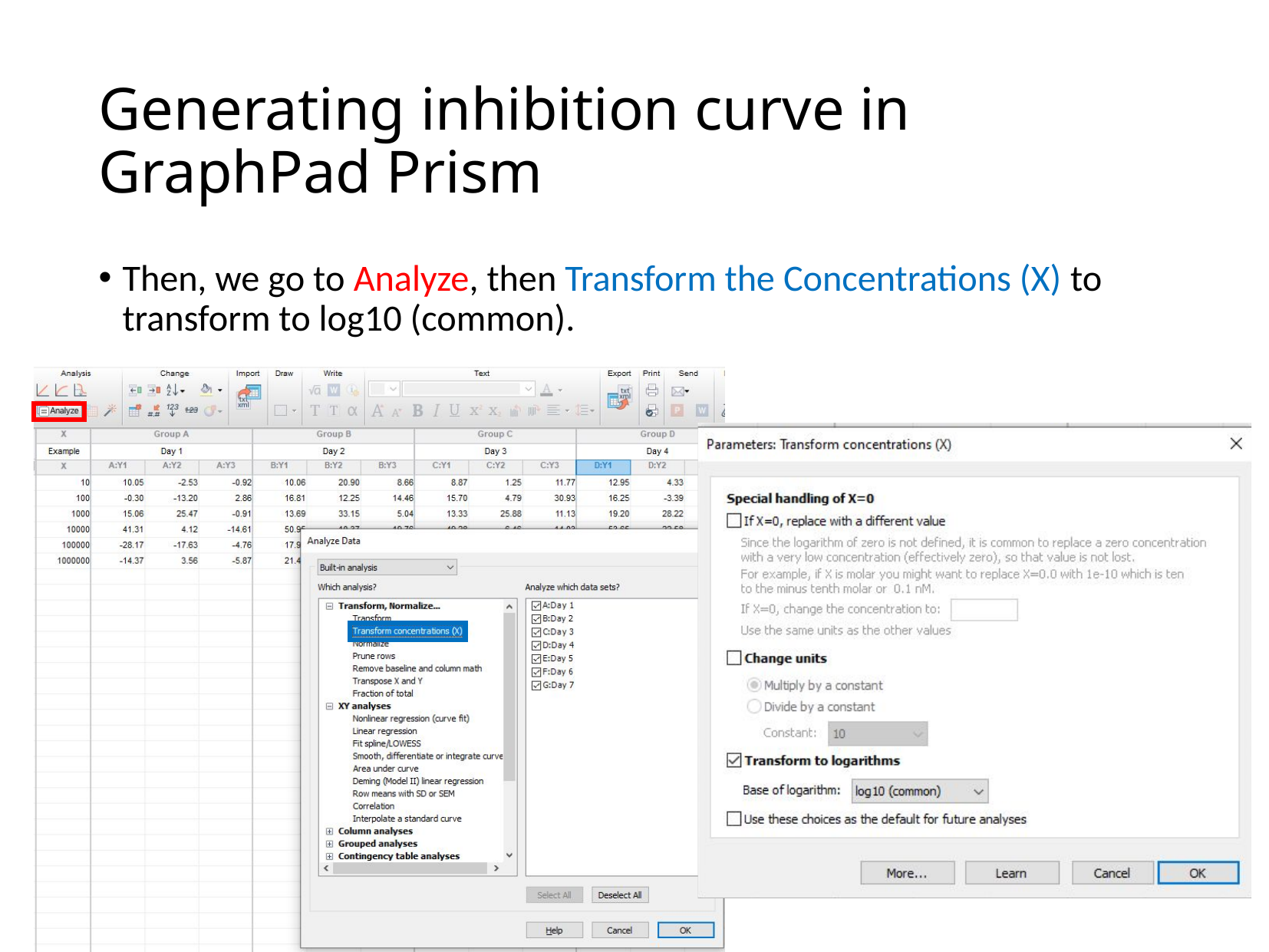

# Generating inhibition curve in GraphPad Prism
Then, we go to Analyze, then Transform the Concentrations (X) to transform to log10 (common).

## Slide 43
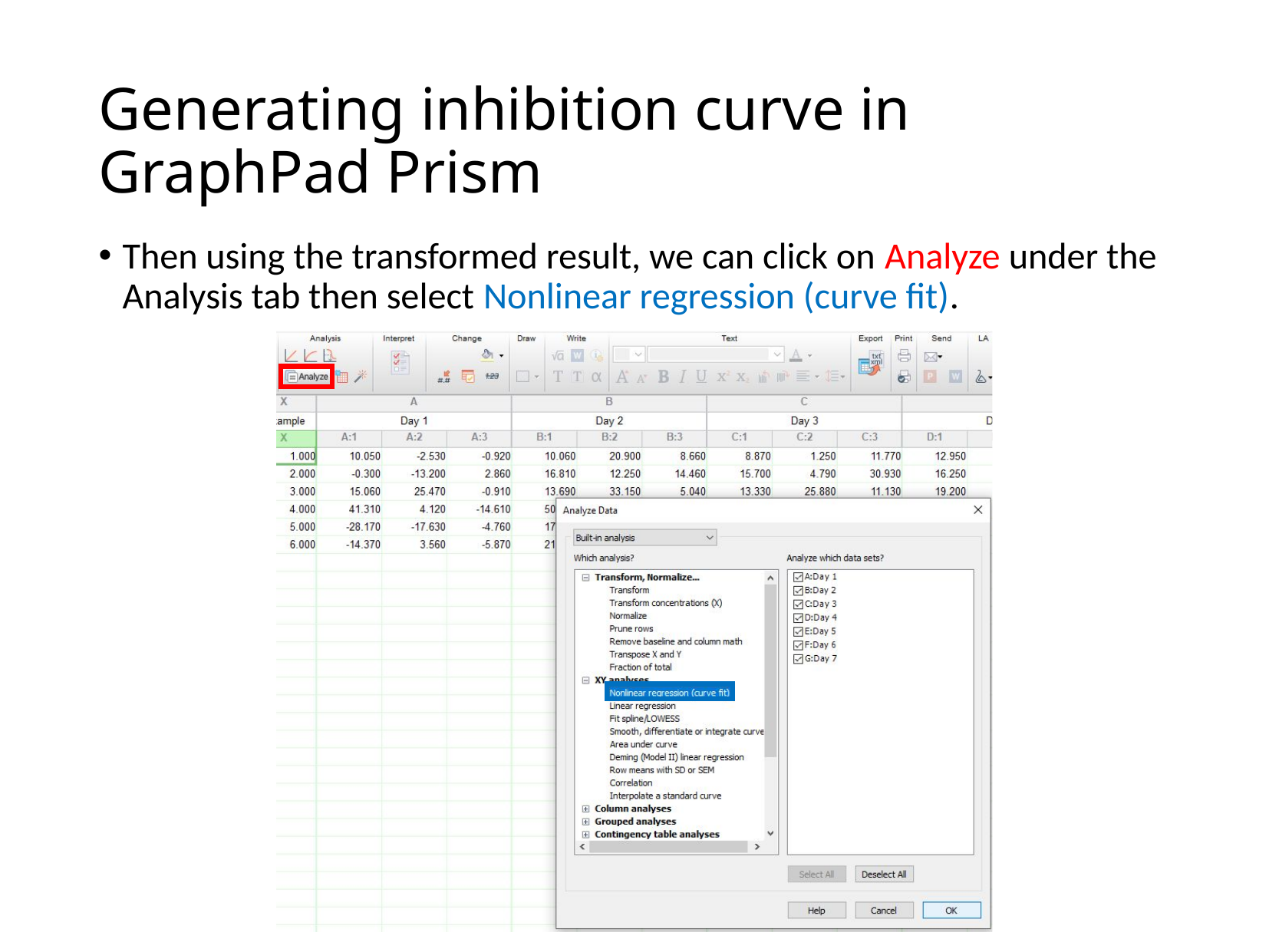

# Generating inhibition curve in GraphPad Prism
Then using the transformed result, we can click on Analyze under the Analysis tab then select Nonlinear regression (curve fit).

## Slide 44
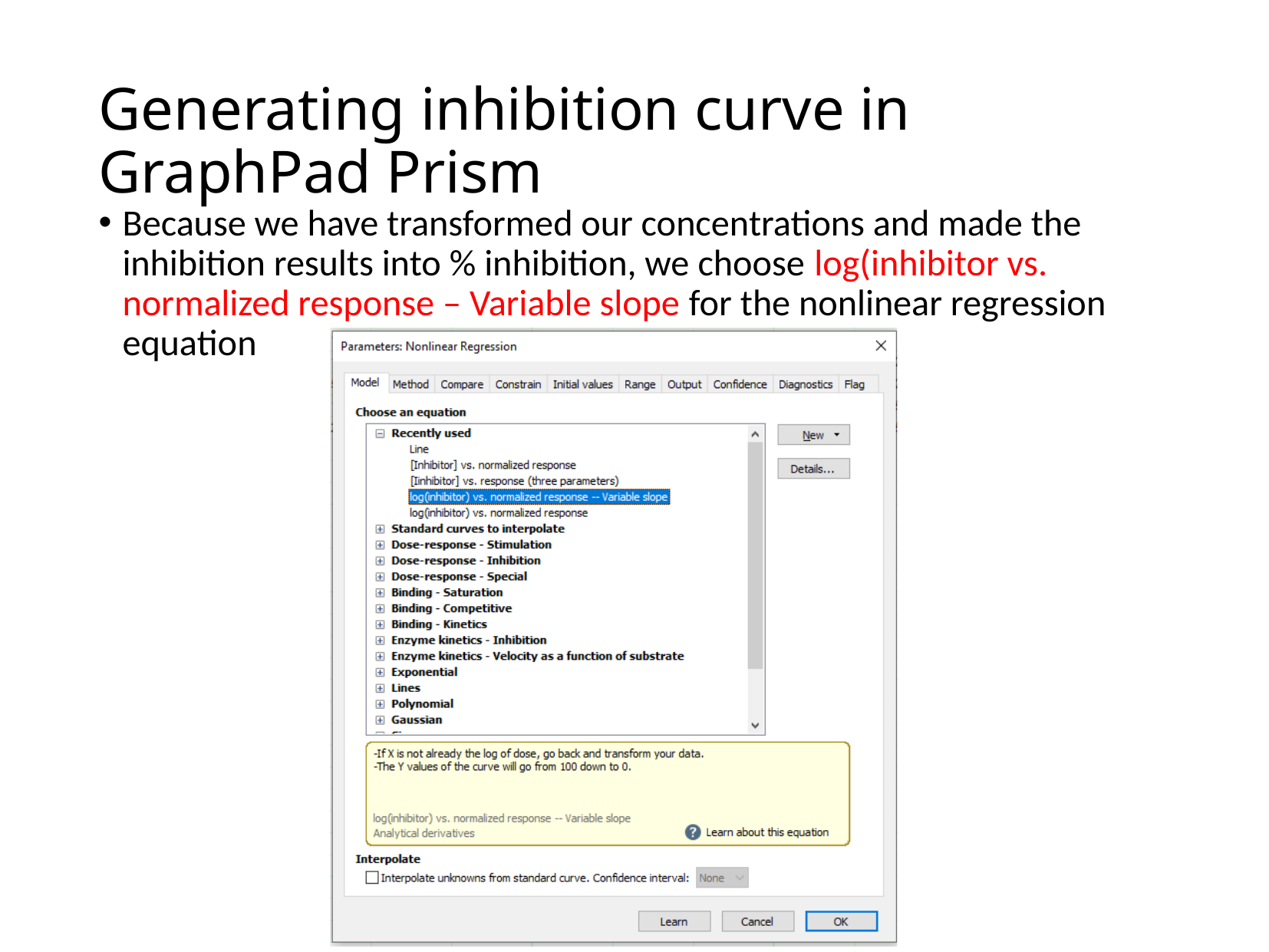

# Generating inhibition curve in GraphPad Prism
Because we have transformed our concentrations and made the inhibition results into % inhibition, we choose log(inhibitor vs. normalized response – Variable slope for the nonlinear regression equation

## Slide 45
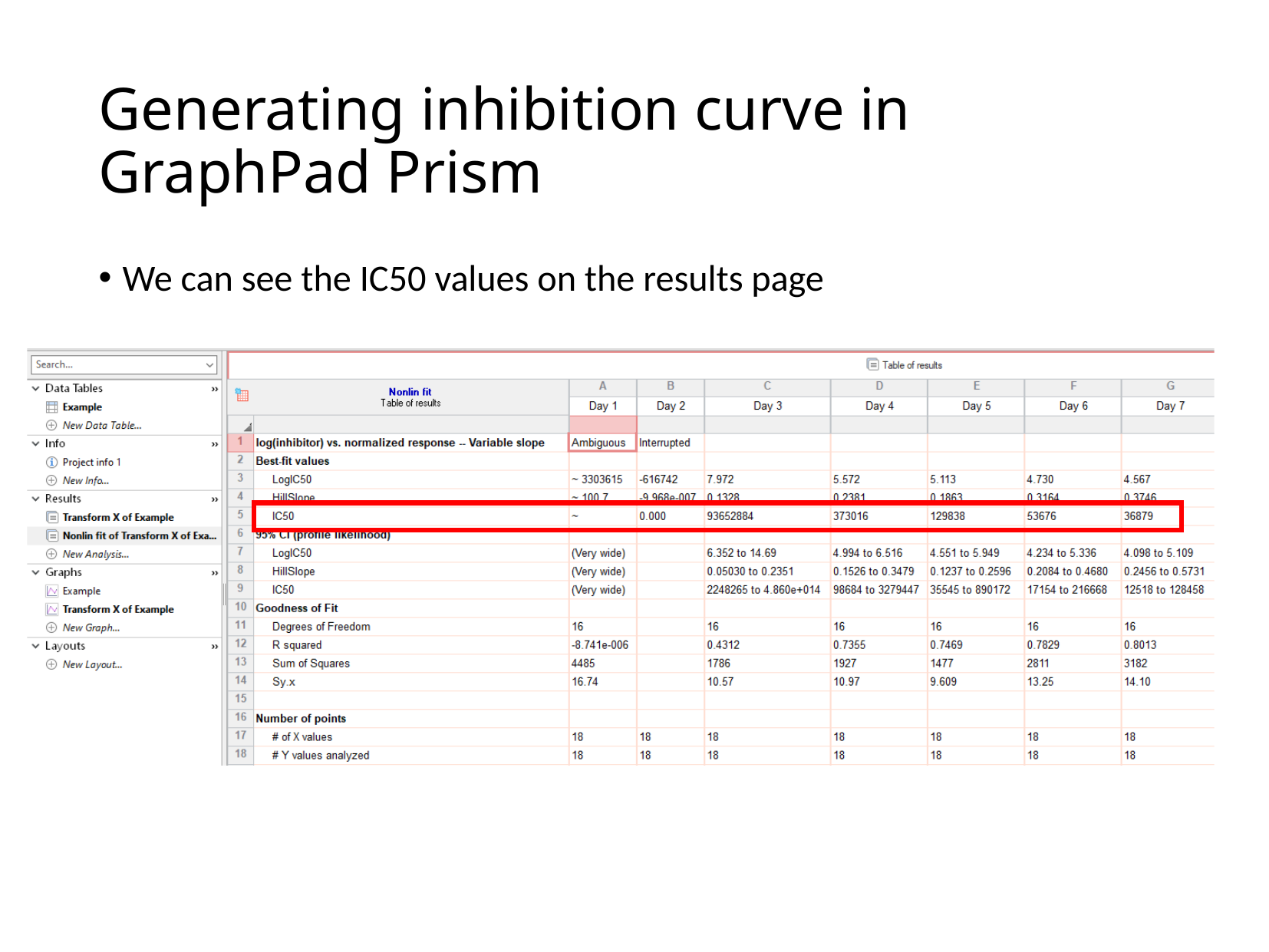

# Generating inhibition curve in GraphPad Prism
We can see the IC50 values on the results page

## Slide 46
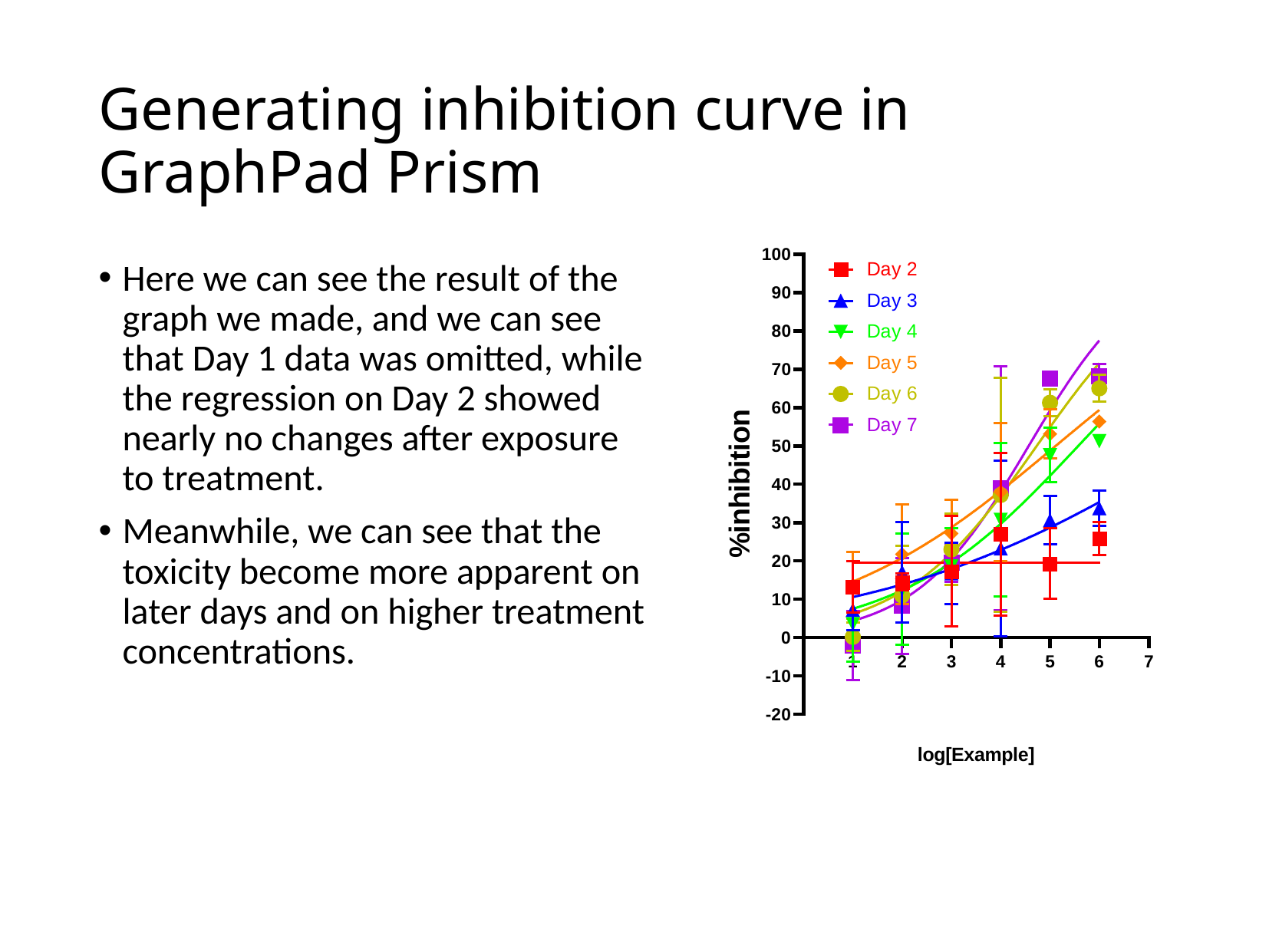

# Generating inhibition curve in GraphPad Prism
Here we can see the result of the graph we made, and we can see that Day 1 data was omitted, while the regression on Day 2 showed nearly no changes after exposure to treatment.
Meanwhile, we can see that the toxicity become more apparent on later days and on higher treatment concentrations.

## Slide 47
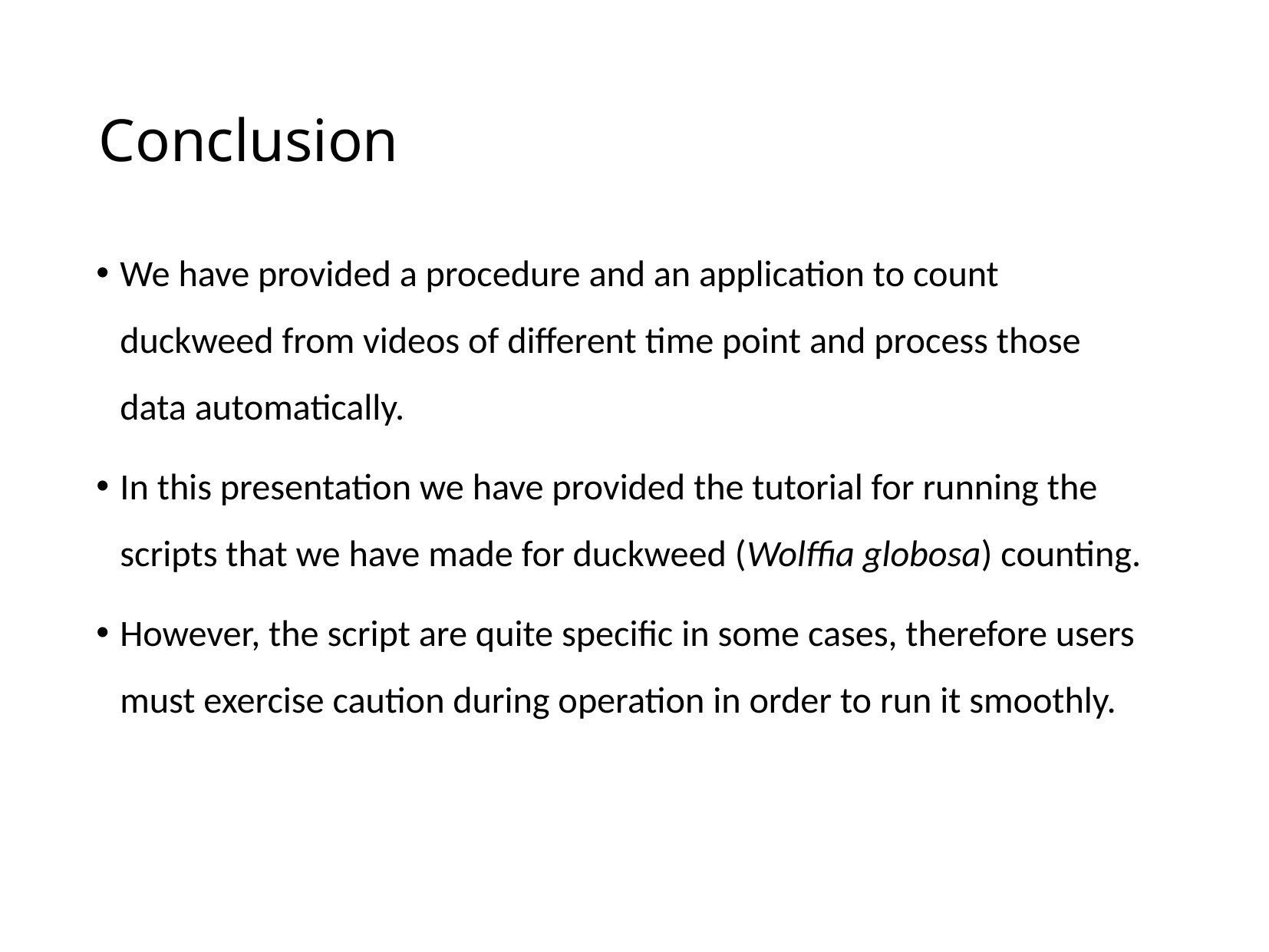

# Conclusion
We have provided a procedure and an application to count duckweed from videos of different time point and process those data automatically.
In this presentation we have provided the tutorial for running the scripts that we have made for duckweed (Wolffia globosa) counting.
However, the script are quite specific in some cases, therefore users must exercise caution during operation in order to run it smoothly.
